# Variations in the Risk of New‐Onset Diabetes Following COVID‐19 Infection Across Body Mass Index, Deprivation, Ethnicity and Geographic Regions: Population‐Based Cohort Study in 42 Million People in England

**DOI:** 10.1111/dom.70856

**Published:** 2026-05-10

**Authors:** Sharmin Shabnam, Cameron Razieh, John Nolan, Nazrul Islam, Genevieve Cezard, Yogini V. Chudasama, Clare L. Gillies, Amitava Banerjee, Angela Wood, Kamlesh Khunti, Francesco Zaccardi, the CVD‐COVID‐UK/COVID‐IMPACT Consortium

**Affiliations:** ^1^ Leicester Real World Evidence Unit, Leicester Diabetes Centre Leicester UK; ^2^ Data Analysis for Social Care and Health Office for National Statistics Newport UK; ^3^ British Heart Foundation Data Science Centre, Health Data Research UK London UK; ^4^ Primary Care Research Centre University of Southampton Southampton UK; ^5^ Department of Public Health and Primary Care University of Cambridge Cambridge UK; ^6^ Institute of Health Informatics University College London London UK; ^7^ British Heart Foundation Cardiovascular Epidemiology Unit, Department of Public Health and Primary Care, and Cambridge Centre for AI in Medicine University of Cambridge Cambridge UK

**Keywords:** BMI, cohort study, COVID‐19, deprivation, diabetes, electronic health records, England, ethnicity, region

## Abstract

**Aims:**

Evidence suggests that COVID‐19 may be associated with an increased risk of diabetes. We aimed to examine this association by investigating the role of socioeconomic and metabolic factors on the risk of new‐onset type 2 (T2D) and type 1 (T1D) diabetes after COVID‐19 diagnosis.

**Materials and Methods:**

We conducted a retrospective, population‐based cohort study using linked electronic health records from NHS England's Secure Data Environment for England via the CVD‐COVID‐UK/COVID‐IMPACT consortium. Adults (≥ 18 years), alive, registered with a general practice within 1 January 2020 and 28 May 2024 were included. Exposed individuals with confirmed COVID‐19 diagnosis and no prior diabetes were matched to up to three unexposed individuals without COVID‐19 and diabetes on age, sex, region and deprivation. Flexible parametric survival models were used to estimate associations between COVID‐19 and incident diabetes by sex and across age, BMI, deprivation, ethnicity, and region.

**Results:**

Of 50 156 810 eligible individuals, 12 859 545 with a COVID‐19 diagnosis and no prior diabetes were matched to 29 221 285 without COVID‐19; the median follow‐up was 2.4 years. Although BMI was strongly and positively associated with the risk of T2D, differences between exposed and unexposed individuals were little to none, with the excess risk concentrated in the first year (e.g., in 70‐year‐old men with BMI 35 kg/m^2^, rates were 44.2 [95% CI: 43.6–44.8] and 44.2 [43.7–44.8] per 1000 person‐years in the exposed and unexposed group, respectively, at 1 year; corresponding figures in women were 28.0 [27.6–28.5] and 29.1 [28.7–29.5]). These rate differences by COVID‐19 exposure were considerably smaller than those across BMI levels: for example, 22 more T2D cases per 1000 person‐years at 1 year for those with a BMI of 35 versus 30 kg/m^2^ in unexposed 70‐year‐old men. Similarly, higher deprivation and Asian ethnicity were also more strongly associated with the risk of T2D than COVID‐19 exposure. There was no evidence of an association between COVID‐19 and T1D across all analyses.

**Conclusions:**

In this cohort, COVID‐19 was associated with a modest, short‐term increase in T2D risk and showed no meaningful association with T1D. Established metabolic, demographic and socioeconomic factors—including age, BMI, deprivation and ethnicity—were more strongly associated with T2D incidence than COVID‐19 exposure.

## 1 Introduction

The COVID‐19 pandemic, caused by the SARS‐CoV‐2 virus, has had far‐reaching consequences beyond acute respiratory illness, including long‐term health complications that continue to emerge [1]. Diabetes was identified early as a major risk factor for severe COVID‐19 outcomes, including critical care admission and mortality [2]. In people with pre‐existing diabetes, poor glycaemic control has been shown to worsen COVID‐19 outcomes [3]; additionally, elevated blood glucose levels, regardless of prior diabetes, have been linked to increased mortality among those with COVID‐19 [4, 5].

Recent studies have revealed a bi‐directional relationship between COVID‐19 and diabetes [4, 6]. A growing body of evidence has highlighted a concerning link between SARS‐CoV‐2 infection and metabolic dysfunction, particularly new onset or worsening of diabetes [7]. However, many of these studies have been limited by short follow‐up periods or selective cohorts [8]. The underlying mechanism also remains under debate [7]. Furthermore, long‐term, population‐level studies investigating new onset diabetes (both type 1 and type 2) following COVID‐19 and comparing this potential excess risk with other well‐established risk factors, namely body mass index (BMI), socioeconomic deprivation, geographic region and ethnicity, remain scarce. These factors are known to contribute to disparities in both COVID‐19 outcomes and diabetes prevalence [9, 10, 11], but their intersection remains underexplored.

This study aimed to address these gaps by assessing the risk of new onset diabetes in individuals without prior diabetes following SARS‐CoV‐2 infection. Using a large, national‐level cohort with extended follow‐up, we examined how BMI, deprivation, ethnicity, and geographical region modify this risk. This research has the potential to further clarify if, and to what extent, COVID‐19 is associated with the risk of new‐onset diabetes and contribute further insights into a potential causal link, while informing clinical guidance and public health policies for diabetes screening strategies following COVID‐19.

## 2 Methods

### 2.1 Data Sources

In this retrospective cohort study, we used the General Practice Extraction Service (GPES) Data for Pandemic Planning and Research (GDPPR) database [12], linked to Hospital Episode Statistics Admitted Patient Care (HES APC) and Office for National Statistics (ONS) death registrations. The datasets were further linked to the Second Generation Surveillance System (SGSS) [13] and the COVID‐19 Hospitalisation in England Surveillance System (CHESS) [14] database. GDPPR includes pseudo‐anonymised individual‐level electronic health records from almost the whole population of England who are registered to a general practice. All datasets were accessed through NHS England's Secure Data Environment (SDE) for England, via the CVD‐COVID‐UK/COVID‐IMPACT Consortium [15]. This consortium is supported by the British Heart Foundation (BHF) Data Science Centre (DSC) and Health Data Research UK (HDR UK) [16]. Additional information on the consortium and the datasets has been previously published [17].

### 2.2 Study Design

The baseline cohort was defined as all individuals who met the following criteria during the study period of 01 January 2020 (study start date) to 28 May 2024 (end date): (1) alive and registered with a general practice in England; (2) aged ≥ 18 years at the study start; and (3) with complete data on age, sex, region and socioeconomic deprivation. Age (estimated from year of birth), sex, region and ethnicity (classified as White, Asian or Asian British, Black or Black British, Mixed or Other and Unknown) were obtained using the most recent non‐missing entry recorded in GDPPR or HES (if missing in GDPPR). Socioeconomic deprivation was derived using the 2019 Index of Multiple Deprivation (IMD) [18], which ranks most recent patient Lower Layer Super Output Areas (LSOA) records linked from primary and secondary care sources in England using seven weighted domains (income, employment, health, education, crime, barriers to housing and services and environment). IMD was categorised into quintiles; quintile 1 represents the most deprived areas and quintile 5 the least deprived. BMI and smoking status were derived using SNOMED‐CT codes from the most recent values in GDPPR within 2 years prior to the index date (defined below).

Within the study period, COVID‐19 diagnosis was defined using multiple data sources: (1) a positive SARS‐CoV‐2 polymerase chain reaction (PCR) or antigen test recorded in the SGSS; (2) a confirmed COVID‐19 diagnosis (using SNOMED‐CT codes) in GDPPR primary care records; (3) a hospital admission record in HES APC with COVID‐19 ICD‐10 codes (U07.1 and U07.2) in any position; (4) any hospital admission recorded in CHESS; or (5) death recorded in ONS data with COVID‐19 ICD‐10 codes in the causes of death field. The index date was defined as the earliest date of a recorded COVID‐19 diagnosis from any of these sources.

The exposed group included individuals diagnosed with COVID‐19 during the study period and with no prior record of diabetes (type 1 diabetes [T1D], type 2 diabetes [T2D], gestational or unspecified diabetes) before the index date. Each exposed individual was randomly matched to up to three unexposed individuals from the baseline cohort based on exact year of birth, sex, region and IMD quintile. Unexposed individuals were assigned the same index date as their matched exposed individual and were required to be registered and alive with no prior COVID‐19 or diabetes at that date. All individuals were followed from the index date until the earliest date of diabetes diagnosis, death, COVID‐19 diagnosis (for unexposed) or end of study. For patients censored on the index date, follow up time was set to 0.5 days (half the time unit in the dataset).

The primary outcomes were the incidence of (a) T2D or (b) T1D after the index date. Diabetes phenotypes were defined based on SNOMED‐CT and ICD‐10 codes using GDPPR, ONS and HES APC data. The first record of diabetes phenotype during the follow‐up period was used to classify cases into T2D and T1D. We also conducted a sensitivity analysis where we applied the Diabetes Data Science Catalyst (i.e., DDSC, a collaboration between the BHF DSC, Diabetes UK and HDR UK) diabetes phenotype algorithm to exclude prevalent diabetes and define incident diabetes as the outcome [19]. This clinician‐verified system combines diagnostic codes with prescribing records, age and glycated haemoglobin measurements to assign individuals to one of several phenotypes (T1D, T2D, gestational diabetes, unspecified or diabetes unlikely). Further details of the algorithm can be found elsewhere [19]. To assess the impact of COVID‐19 severity, we conducted a further sensitivity analysis restricted to individuals with a COVID‐19 diagnosis in hospital records (HES APC or CHESS) and their matched controls.

### 2.3 Statistical Analysis

Baseline characteristics (overall and stratified by sex) were summarised for the unexposed (no COVID‐19) and the exposed (COVID‐19) groups using descriptive statistics: categorical variables using counts and percentages, and continuous variables using mean with standard deviation (SD) or median with interquartile range (IQR) as appropriate.

For both groups, we estimated the total number of incident diabetes events, total person‐years of follow‐up and crude and age‐standardised (using the age distribution of the unexposed group as the standard population) incidence rates per 100 000 person‐years, overall and stratified by sex.

We used flexible parametric Royston‐Parmar survival models [20] to estimate associations between COVID‐19 exposure status and incident diabetes (separately T2D and T1D), with time‐to‐event from the index date; all models were stratified by sex and included a natural cubic spline (with 4 degrees of freedom) transformation of age to allow for a non‐linear effect. In four separate models, we included interactions with the exposure status to investigate the effect modification of BMI (main effect modifier, modelled with a natural cubic spline with 4 degrees of freedom), deprivation (quintiles), ethnicity or region; each of the four analyses was repeated including a time‐varying exposure (TVE) effect. For the analyses with BMI, individuals with missing BMI values were excluded; all other analyses had no missing data and used the full cohort. From the final models, outcome‐specific hazard rates were estimated over follow‐up time conditional on the exposure status and age (40, 50, 60 and 70 years), BMI, deprivation, ethnicity and region. In a sensitivity analysis, all models were repeated using the DDSC algorithm diabetes definition to assess the robustness of findings.

Data curation were performed using Python (version 3.9.21); statistical analysis was conducted in R (version 4.1.3) using the *rstpm2* package. Results of the study were reported following the RECORD guidelines for the reporting of studies conducted using observational routinely collected health data (checklist provided in the Supporting Information). This analysis was performed according to a prespecified plan published on GitHub, along with the phenotyping codes and analysis code (https://github.com/BHFDSC/CCU043_01).

## 3 Results

### 3.1 Characteristics of the Cohort

Of the 50 156 810 eligible individuals, 12 859 545 with a diagnosis of COVID‐19 and no prior diabetes were included in the exposed group; they were matched with 29 221 285 unexposed individuals without COVID‐19 and diabetes at the matched index date, resulting in a final cohort of 42 080 830 (Figure S1).

Women comprised a slightly higher proportion in both groups (7 163 970 [55.7%] in the exposed group; 15 608 720 [53.4%] in the unexposed) (Table 1). Mean age was 44.4 [SD 16.4] years in the exposed group and 43.6 [16.0] in the unexposed. The exposed group had a higher proportion of White (10 884 555 [84.6%] vs. 22 128 830 [75.7%]) and a lower proportion of Asian (1 015 500 [7.9%] vs. 3 413 360 [11.7%]) and Black (385 580 [3.0%] vs. 1 254 915 [4.3%]) individuals compared to those unexposed. Distributions across deprivation and regions were similar. The exposed group had fewer smokers and a slightly higher mean BMI (28.0 [SD 6.7] vs. 27.6 [6.5] kg/m^2^) than the unexposed (Table 1). The cohort of individuals with complete BMI (used in BMI analysis) had comparable characteristics to the main cohort aside from being slightly older and a higher proportion of women and smokers (Table S1).

**TABLE 1:** Characteristics of the cohort, stratified by exposure status and sex.

Category	Unexposed (no COVID‐19)			Exposed (COVID‐19)		
	Overall *N* = 29 221 285	Women *N* = 15 608 720 (53.4%)	Men *N* = 13 612 565 (46.6%)	Overall *N* = 12 859 545	Women *N* = 7 163 970 (55.7%)	Men *N* = 5 695 575 (44.3%)
Age (years), mean (SD)	43.6 (16.0)	44.0 (16.4)	43.1 (15.5)	44.4 (16.4)	44.4 (16.5)	44.4 (16.2)
Age (years), *n* (%)						
18–29	6 428 265 (22.0)	3 469 590 (22.2)	2 958 675 (21.7)	2 631 565 (20.5)	1 483 715 (20.7)	1 147 850 (20.2)
30–39	6 679 750 (22.9)	3 468 755 (22.2)	3 210 995 (23.6)	2 853 570 (22.2)	1 596 335 (22.3)	1 257 235 (22.1)
40–49	5 565 760 (19.0)	2 827 760 (18.1)	2 737 995 (20.1)	2 533 640 (19.7)	1 411 970 (19.7)	1 121 670 (19.7)
50–59	5 024 480 (17.2)	2 750 290 (17.6)	2 274 195 (16.7)	2 208 815 (17.2)	1 236 785 (17.3)	972 030 (17.1)
60–69	2 891 660 (9.9)	1 600 250 (10.3)	1 291 415 (9.5)	1 293 390 (10.1)	703 485 (9.8)	589 905 (10.4)
70–79	1 620 360 (5.5)	875 425 (5.6)	744 935 (5.5)	758 290 (5.9)	390 645 (5.5)	367 650 (6.5)
80+	1 011 010 (3.5)	616 655 (4.0)	394 355 (2.9)	580 270 (4.5)	341 035 (4.8)	239 235 (4.2)
Ethnicity, *n* (%)						
White	22 128 830 (75.7)	11 958 005 (76.6)	10 170 820 (74.7)	10 884 555 (84.6)	6 108 040 (85.3)	4 776 515 (83.9)
Asian or Asian British	3 413 360 (11.7)	1 831 830 (11.7)	1 581 530 (11.6)	1 015 500 (7.9)	547 420 (7.6)	468 075 (8.2)
Black or Black British	1 254 915 (4.3)	695 850 (4.5)	559 065 (4.1)	385 580 (3.0)	219 960 (3.1)	165 620 (2.9)
Mixed/Other	1 361 525 (4.7)	723 975 (4.6)	637 545 (4.7)	436 960 (3.4)	242 610 (3.4)	194 350 (3.4)
Unknown	1 062 660 (3.6)	399 060 (2.6)	663 600 (4.9)	136 950 (1.1)	45 935 (0.6)	91 015 (1.6)
IMD (quintiles), *n* (%)						
1 (Most deprived)	5 555 915 (19.0)	3 018 880 (19.3)	2 537 040 (18.6)	2 408 750 (18.7)	1 356 360 (18.9)	1 052 390 (18.5)
2	6 006 340 (20.6)	3 229 230 (20.7)	2 777 110 (20.4)	2 598 660 (20.2)	1 449 485 (20.2)	1 149 175 (20.2)
3	5 967 785 (20.4)	3 193 220 (20.5)	2 774 565 (20.4)	2 620 290 (20.4)	1 462 175 (20.4)	1 158 120 (20.3)
4	5 889 230 (20.2)	3 118 650 (20.0)	2 770 575 (20.4)	2 628 780 (20.4)	1 461 805 (20.4)	1 166 975 (20.5)
5 (Least deprived)	5 802 020 (19.9)	3 048 745 (19.5)	2 753 275 (20.2)	2 603 065 (20.2)	1 434 150 (20.0)	1 168 915 (20.5)
Region, *n* (%)						
East Midlands	2 465 465 (8.4)	1 309 435 (8.4)	1 156 030 (8.5)	1 101 595 (8.6)	613 855 (8.6)	487 740 (8.6)
East of England	3 189 125 (10.9)	1 711 700 (11.0)	1 477 420 (10.9)	1 400 175 (10.9)	782 200 (10.9)	617 975 (10.9)
London	4 798 535 (16.4)	2 580 080 (16.5)	2 218 455 (16.3)	1 971 520 (15.3)	1 079 645 (15.1)	891 875 (15.7)
North East	1 403 675 (4.8)	729 915 (4.7)	673 760 (4.9)	663 255 (5.2)	370 850 (5.2)	292 405 (5.1)
North West	3 882 045 (13.3)	2 040 170 (13.1)	1 841 875 (13.5)	1 761 010 (13.7)	977 665 (13.6)	783 345 (13.8)
South East	4 816 590 (16.5)	2 601 630 (16.7)	2 214 955 (16.3)	2 088 160 (16.2)	1 164 870 (16.3)	923 290 (16.2)
South West	2 794 105 (9.6)	1 517 490 (9.7)	1 276 615 (9.4)	1 228 180 (9.6)	695 495 (9.7)	532 685 (9.4)
West Midlands	3 040 160 (10.4)	1 612 355 (10.3)	1 427 805 (10.5)	1 371 715 (10.7)	765 555 (10.7)	606 160 (10.6)
Yorkshire and The Humber	2 831 585 (9.7)	1 505 940 (9.6)	1 325 645 (9.7)	1 273 930 (9.9)	713 835 (10.0)	560 100 (9.8)
Smoking status, *n* (%)						
No	9 107 180 (31.2)	5 975 820 (38.3)	3 131 360 (23.0)	4 258 130 (33.1)	2 763 985 (38.6)	1 494 145 (26.2)
Yes	3 076 985 (10.5)	1 527 760 (9.8)	1 549 220 (11.4)	1 064 180 (8.3)	582 010 (8.1)	482 170 (8.5)
Ex	3 672 540 (12.6)	1 979 930 (12.7)	1 692 610 (12.4)	2 003 005 (15.6)	1 096 815 (15.3)	906 195 (15.9)
Missing	13 364 580 (45.7)	6 125 210 (39.2)	7 239 370 (53.2)	5 534 230 (43.0)	2 721 160 (38.0)	2 813 070 (49.4)
BMI categories (kg/m^2^), *n* (%)						
Underweight [< 18.5]	400 085 (2.8)	305 935 (3.4)	94 145 (1.7)	187 700 (2.5)	143 110 (3.0)	44 590 (1.6)
Normal [18.5–24.9]	5 150 670 (35.9)	3 451 385 (38.7)	1 699 280 (31.2)	2 524 825 (33.9)	1 735 645 (36.9)	789 180 (28.8)
Overweight [25–29.9]	4 684 455 (32.6)	2 551 740 (28.6)	2 132 715 (39.2)	2 429 885 (32.6)	1 348 050 (28.6)	1 081 835 (39.5)
Obesity [30–39.9]	3 455 755 (24.1)	2 115 430 (23.7)	1 340 330 (24.6)	1 911 405 (25.7)	1 182 555 (25.1)	728 850 (26.6)
Severe obesity [≥ 40]	669 940 (4.7)	496 725 (5.6)	173 220 (3.2)	394 130 (5.3)	296 840 (6.3)	97 290 (3.5)
BMI values (kg/m^2^)						
Mean (SD)	27.6 (6.5)	27.5 (7.0)	27.7 (5.8)	28.0 (6.7)	27.9 (7.1)	28.1 (5.9)
Median [IQR]	26.5 [23.1, 30.8]	26.1 [22.6, 31.0]	26.9 [24.0, 30.4]	26.8 [23.4, 31.2]	26.5 [22.9, 31.5]	27.2 [24.3, 30.8]
Missing, *n* (%)	14 860 385 (50.9)	6 687 510 (42.8)	8 172 875 (60.0)	5 411 595 (42.1)	2 457 765 (34.3)	2 953 830 (51.9)

*Note:* Numbers are rounded to the nearest five as per NHS Secure Data Environment (SDE) safe output guidelines.

Abbreviations: BMI, body mass index; IMD, index of multiple deprivation; SD, standard deviation.

### 3.2 Incidence of Diabetes

The incidence rates of T2D were consistently higher in the exposed (median follow‐up of 2.4 years; IQR: 2.2–2.9 for both sexes) compared to the unexposed (median follow‐up of 2.4 years; IQR: 2.1–2.8 for men, 1.8–2.7 for women) group (Table 2). Among men, the age‐standardised incidence rate was 666 versus 546 per 100 000 person‐years in the exposed and unexposed groups, respectively; corresponding rates for women were 443 versus 405 per 100 000 person‐years. The age‐standardised incidence rates of T1D among men were 12 in both groups, while for women rates were 8 (unexposed) and 7 (exposed) per 100 000 person‐years (Table 2). Individuals with complete BMI data (Table S2) had markedly higher incidence rates than the main cohort.

**TABLE 2:** Number of events, total follow‐up time and incidence rates of diabetes outcomes, stratified by sex and COVID‐19 exposure status.

Sex	Exposure	Events	Person‐years	Median [IQR] follow up (years)	Crude incidence rate (per 100 000 person‐years)	Age‐standardised incidence rate (per 100 000 person‐years)
Type 2 diabetes						
Men	Unexposed	165 920	31 137 905	2.4 [2.1, 2.8]	533	546
	Exposed	95 630	14 348 975	2.4 [2.2, 2.9]	666	666
Women	Unexposed	139 885	33 811 950	2.4 [1.8, 2.7]	414	405
	Exposed	79 020	17 990 575	2.4 [2.2, 2.9]	439	443
Type 1 diabetes						
Men	Unexposed	3725	31 348 355	2.4 [2.1, 2.8]	12	12
	Exposed	1780	14 481 375	2.4 [2.3, 2.9]	12	12
Women	Unexposed	2750	33 990 220	2.4 [1.8, 2.7]	8	8
	Exposed	1265	18 100 095	2.4 [2.2, 2.9]	7	7

*Note:* ‘Exposed’ group consisted of individuals who had at least one recorded diagnosis of COVID‐19 during the study period and did not have any record of prevalent diabetes. ‘Unexposed’ refers to individuals with no record of diabetes or COVID‐19 diagnosis on or before the matched index date (earliest date of diagnosis of COVID‐19). Numbers are rounded to the nearest five as per NHS Secure Data Environment (SDE) safe output guidelines.

Abbreviation: IQR, interquartile range.

### 3.3 Type 2 Diabetes

**FIGURE 1:**
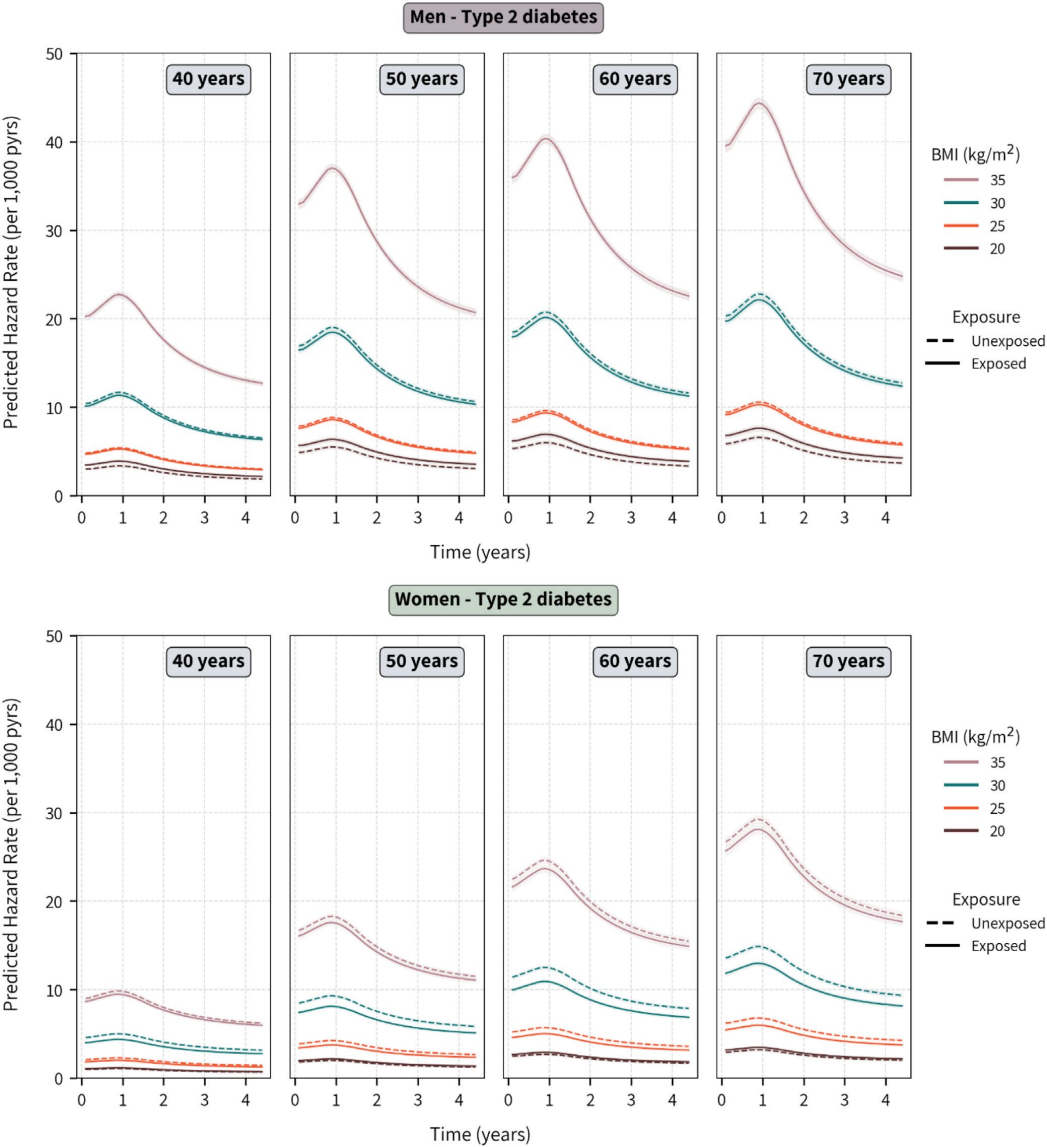
Hazard rates of type 2 diabetes over time by body mass index, age and exposure status, stratified by sex. Sex‐stratified hazard rates obtained from flexible parametric survival models including natural cubic splines (4 degrees of freedom) of age and BMI and an interaction between BMI and exposure status. Time represents follow‐up time from the index date (date of COVID‐19 diagnosis for exposed individuals and the matched index date for unexposed individuals). Solid lines represent exposed individuals (COVID‐19), and dashed lines represent unexposed individuals (no COVID‐19). Shaded areas represent 95% confidence intervals. BMI, body mass index; pyrs, person‐years.

Figure 1 shows the hazard rates of T2D (later referred to as rates) over time by age, sex, BMI and COVID‐19 exposure status. Differences between exposed and unexposed individuals were negligible among men and minimal among women, with the overall rates lower in women. Rates were high at baseline, peaked around 1 year after the index date, and then gradually declined. Among 70‐year‐olds with a BMI 35 kg/m^2^ the 1‐year rate was 44.2 [95% CI: 43.6–44.8] and 44.2 [43.7–44.8] per 1000 person‐years in the exposed and unexposed men, respectively; corresponding rates for women were 28.0 [27.6–28.5] and 29.1 [28.7–29.5]. The absolute differences between exposed and unexposed groups were much smaller than those across levels of BMI or age; for example, among unexposed 70‐year‐old men, the rate difference between a BMI of 30 kg/m^2^ and 35 kg/m^2^ was 21.5 (22.7 vs. 44.2, respectively), compared to a difference of only 0.01 between exposed and unexposed 70‐year‐old men with BMI 35 kg/m^2^ (Figure 1). The pattern of the results was similar in models with a TVE effect (Figure S2).

Figures S3, S5 and S7 present analyses by deprivation, ethnicity and region; corresponding plots from models with a TVE effect are reported in Figures S4, S6 and S8. Exposed individuals had higher hazard rates for T2D than unexposed individuals across these analyses. The highest rate was observed among those exposed and in the most deprived quintile, particularly for older men (e.g., in 70‐year‐old men at 1.2 years: 23.7 [95% CI: 23.4–24.1] and 18.8 [18.5–19.0] per 1000 person‐years in the exposed and unexposed group, respectively; corresponding rates for women were 16.7 [16.5–17.0] and 15.7 [15.5–15.9]) (Figure S3). However, corresponding rates at 1.2 years in the unexposed men and women in the least deprived decile were 9.7 [9.6–9.9] and 6.2 [6.1–6.3], respectively.

Asian or Asian British individuals showed the highest risks across all ethnicities (in 70‐year‐old men at 1.2 years: 42.0 [41.2–42.8] and 33.2 [32.7–33.7] per 1000 person‐years in the exposed and unexposed group, respectively; corresponding rates in women were 26.7 [26.1–27.3] and 23.8 [23.4–24.2]) (Figure S6). On the other hand, corresponding rates at 1.2 years in the unexposed White men and women were 12.0 [11.9–12.1] and 8.3 [8.2–8.4], respectively. Regional variation was modest, although somewhat higher rates were observed with the West Midlands and London (Figure S7). Differences in rates were (considerably) larger across levels of the modifier (i.e., levels of BMI or deprivation) than between exposed and unexposed individuals. Patterns were consistent in TVE models, though rate differences between exposed and unexposed were slightly higher during the first year (Figures S4, S6 and S8).

### 3.4 Type 1 Diabetes

**FIGURE 2:**
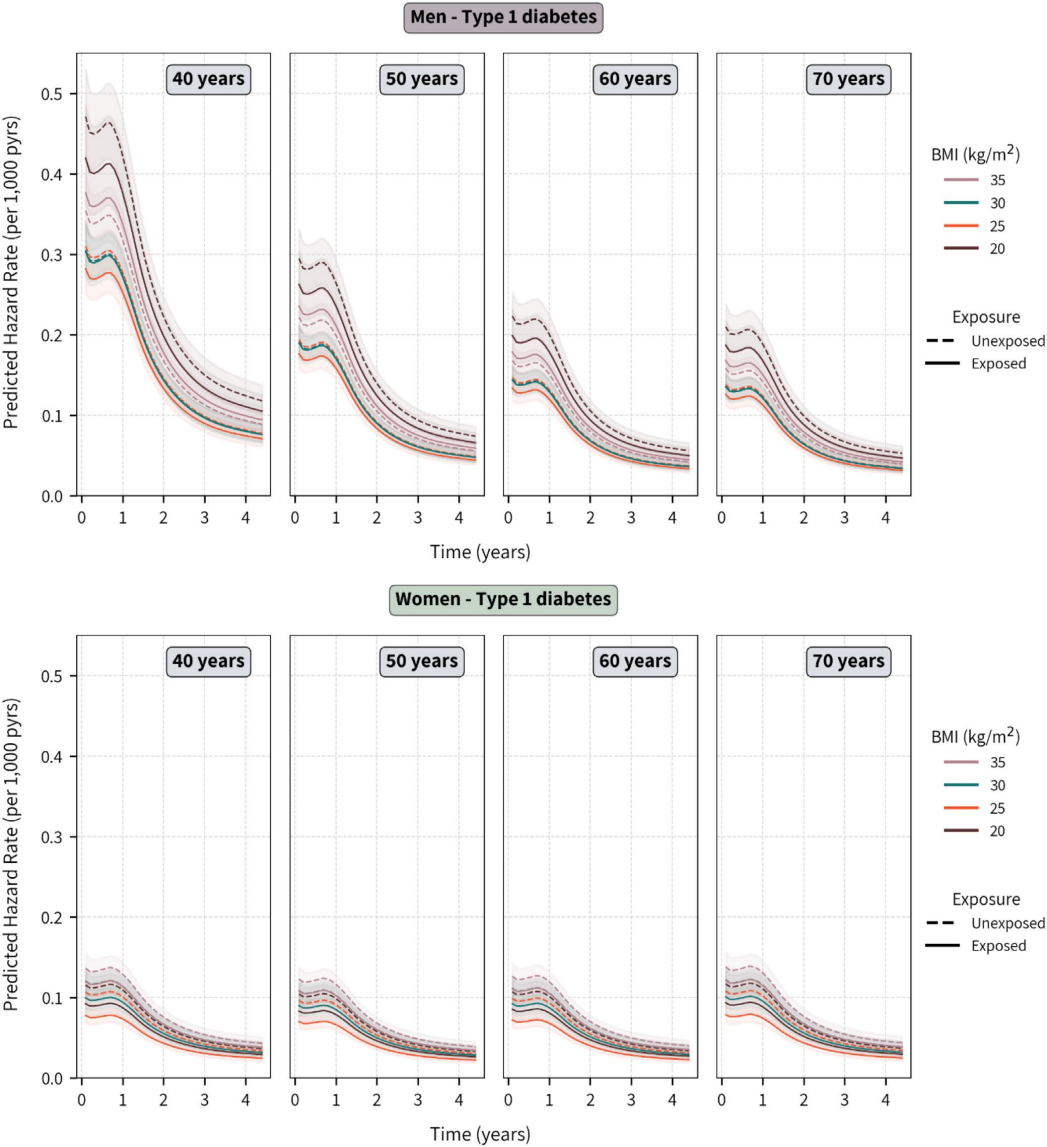
Hazard rates of type 1 diabetes over time by body mass index, age and exposure status, stratified by sex. Sex‐stratified hazard rates obtained from flexible parametric survival models including natural cubic splines (4 degrees of freedom) of age and BMI and an interaction between BMI and exposure status. Solid lines represent exposed individuals (COVID‐19), and dashed lines represent unexposed individuals (no COVID‐19). Shaded areas represent 95% confidence intervals. BMI, body mass index; pyrs, person‐years.

Hazard rates of incident T1D showed no meaningful difference between exposed and unexposed individuals. Across BMI levels, rates were marginally higher (with overlapping 95% CIs) in unexposed individuals (Figure 2) but converged after 2 years. The highest risk occurred at lower BMI, particularly for younger adults (in 40‐year‐old men with BMI 20 kg/m^2^, rates at 0.1 years were 0.42 [0.36–0.49] and 0.47 [0.42–0.53] per 1000 person‐years in the exposed and unexposed group, respectively; corresponding rates among women were 0.09 [0.08–0.11] and 0.12 [0.10–0.13]) (Figure 2). The inclusion of TVE effect resulted in very similar results (Figure S9).

The models with deprivation (Figure S10), ethnicity (Figure S12) and region (Figure S14) followed similar patterns. There was no clear distinction within deprivation quintiles and regions, with CIs frequently overlapping between subgroups. The strongest disparities appeared for younger Black or Black British men, who reported higher rates compared to other ethnic groups; yet, rates were not different between exposed and unexposed (Figure S12). The inclusion of a TVE effect rate for the exposed individuals showed steeper declines during the first year (Figures S11, S13, S15).

### 3.5 Sensitivity Analysis

The sensitivity analysis cohort using the DDSC algorithm to define diabetes included 42 062 890 individuals (17 940 [0.04%] excluded for having pre‐existing diabetes defined by the algorithm) (Figure S16); baseline characteristics were similar to the main cohort (Table S3). The incidence rates of T2D were slightly lower (Table S4) but the hazard rates followed patterns similar to those observed in the main analysis, with rates slightly higher around 1 year, followed by a sharper decline (Figures S17–S32).

Similar patterns were observed when analyses were restricted to hospitalised COVID‐19 cases (Figures S33–S40). Although hospitalised individuals showed higher absolute risks of T2D, the excess risk remained largely confined to the early period. Differences in T2D risk across BMI were larger than those between exposed and unexposed individuals, supporting the main findings. For T1D, risks remained low with no sustained excess following COVID‐19 hospitalisation.

## 4 Discussion

In this nationwide, population‐based cohort study of over 42 million individuals, we examined the association between COVID‐19 and T1D and T2D, separately, accounting for demographic, socioeconomic and metabolic factors. COVID‐19 was associated with an increased risk of T2D; however, the excess risk was not consistently observed and, more importantly, it was of small magnitude and restricted to the first year after infection. Differences in T2D risk across traditional (causal) risk factors were substantially larger than those between people with and without COVID‐19. Indeed, while higher levels of BMI were strongly associated with the risk of T2D (i.e., in 70‐year‐old men without COVID‐19, a BMI from 30 kg/m^2^ to 35 kg/m^2^ was associated with 22 more cases of T2D per 1000 person‐years), across all ages and BMI levels the excess rates comparing those exposed and unexposed to COVID‐19 were close to zero. Higher deprivation and Asian ethnicity were also associated with increased incidence of T2D. For T1D, COVID‐19 exposure had no association with the risk. Alternative modelling strategies (time‐varying effect of COVID‐19), diabetes definitions (DDSC algorithm), or analyses restricted to hospitalised COVID‐19 cases largely confirmed and strengthened the robustness of our main results.

### 4.1 Findings in Context

Our study addresses an important gap by examining the association between COVID‐19 and diabetes within the context of metabolic and sociodemographic risk factors. Prior studies have reported varying degrees of elevated diabetes risk following COVID‐19, depending on methodology, follow up time, control selection, infection severity and population demographics [8, 21, 22, 23]. Several have explored subgroup effects with inconsistent findings. One US Veterans Affairs study reported 37% and 28% higher risk of diabetes (any type) for men and women with COVID‐19 [22], whereas another using the same database found an increase only in men [23]. Risk was also higher among older adults, Black individuals, and those with higher BMI [22]. In contrast, our work utilises a large, nationally representative population with detailed phenotyping and over 4 years of follow‐up, analysing T1D and T2D separately, which have been overlooked by most of the previous studies. Our results further highlight the excess risk was concentrated in the first year post‐infection, within all subgroups, and a slightly increased risk in urban regions such as London and the West Midlands.

Only a few studies have explicitly examined changes in risk of T2D or T1D associated with COVID‐19 over time, but most report a decline [21, 23, 24, 25]. The English cohort study found that the risk of T2D was about four times higher in the first 4 weeks before the vaccine rollout, which diminished but persisted into the second year [21]. Another UK cohort reported a sharp rise in the first month (Rate Ratio [RR] for T2D and T1D combined: 1.81; 95% CI: 1.51–2.19) but a return to baseline within a year (1.07 [0.99–1.16]) [24]. A meta‐analysis found the highest risk (of any type of diabetes) was within the first 3 months which declined but did not disappear at more than 6 months [26]. We also observed this time‐limited effect in our analysis, with rates largely converging between exposed and unexposed groups beyond the first year.

Previous literature on the relationship between COVID‐19 and T1D remains mixed, with no consistent evidence of a sustained increase associated with the infection [21, 27, 28, 29, 30, 31]. Although a recent meta‐analysis of children and adolescents reported 14%–27% higher incidence rates during the first 2 years of the pandemic compared to before [28], several studies found no association between SARS‐CoV‐2 infection and incident T1D [29, 30, 31]. The English cohort study among adults found a short‐term elevation in incidence during the first 4 weeks post‐infection, with no sustained risk thereafter [21]. Our findings are consistent with these reports, suggesting that any excess risk of T1D following COVID‐19 was not sustained within the observed follow‐up period.

While previous studies reported diabetes risk by sociodemographic characteristics, our study adds novelty by examining these factors as effect modifiers of the association between COVID‐19 and diabetes. The lack of a clear COVID‐19‐related excess risk across BMI levels and the much stronger positive association between BMI and risk of T2D highlight the comparative role of COVID‐19 and BMI, with BMI being a stronger risk factor [32]. However, this finding does not exclude the possibility that COVID‐19 may contribute to diabetes risk through multifactorial pathways. The impact of COVID‐19 became more visible across levels of deprivation, ethnic groups or English regions (likely because these factors capture broader social and structural inequalities that shape both diabetes susceptibility and healthcare access [33]), however, differences in T2D rates were larger across deprivation or ethnicity than between individuals with and without COVID‐19.

Several biological mechanisms have been proposed to explain the slightly higher diabetes risk following COVID‐19, including beta‐cell dysfunction and systemic inflammation [4]. If SARS‐CoV‐2 directly caused diabetes through these mechanisms, we would expect a stronger and more sustained association. However, when we modelled a time‐varying effect of COVID‐19, we observed a peak in the rates just after COVID‐19 diagnosis followed by a decline, as also seen in numerous other investigations relying on electronic health records. This may reflect a detection bias [34], as individuals with COVID‐19, particularly those hospitalised or with pre‐existing conditions, likely had increased healthcare contact, creating more opportunities for diabetes testing and earlier diagnosis. Since an estimated 30% of people with T2D are undiagnosed in the UK [35], those already at risk or in prediabetic stages may have been diagnosed sooner due to increased healthcare interaction. Furthermore, the absence of a population‐level rise in diabetes incidence after the pandemic may also be consistent with a modest association. Based on prior estimates of the strength of the association [22], one would expect approximately three times as many new T2D cases in England compared to the pre‐pandemic period [6]; however, despite the need for more robust and up‐to‐date data on recent trends in diabetes incidence, no such increase has been reported to date. However, the absence of a strong or sustained association does not necessarily exclude the role of COVID‐19, and differences in infection severity, antiviral medications, vaccination coverage and healthcare utilisation across populations may also contribute to the variation in observed incidence trends.

Regardless of whether the association between COVID‐19 and T2D is causal, our findings carry important clinical and public health implications. They demonstrate that BMI, a well‐established causal risk factor for T2D [36], has a substantially stronger association with the risk of T2D than COVID‐19. Moreover, the pronounced variations in the risk of T2D across levels of deprivation and ethnicity further emphasise that strategies focusing on T2D prevention should still prioritise addressing intersectional inequalities and reducing obesity. Although our findings do not support broad population‐level additional or alternative screening strategies specifically for individuals with a history of COVID‐19, further research is needed to determine whether specific subgroups of individuals following COVID‐19 may benefit from targeted screening or monitoring.

### 4.2 Strengths and Limitations

A major strength of our study is its large, representative population, providing sufficient power to make robust comparisons across key risk factors. The use of a time‐varying modelling strategy captured the dynamic effect of COVID‐19 over time, without assuming constant effect. The 4‐year follow‐up, longer than most of the previous studies, offered important insight into both short‐ and long‐term diabetes risk following COVID‐19, clarifying the temporal dynamics of risk. However, this period also coincided with the rollout of vaccination programmes and the introduction of antiviral treatments, which may have affected infection severity and subsequent diabetes risk.

Several other limitations should also be noted. First, reliance on electronic health records may introduce potential misclassification of both exposure and outcomes. Early in the pandemic, limited PCR testing likely missed mild or asymptomatic cases; later, widespread lateral flow tests were introduced but these had lower sensitivity and were inconsistently reported [37]. After the end of free testing in 2022, many infections likely went undocumented. These factors may have led to underestimation of COVID‐19 exposure and its associated risk. For diabetes, although coding quality in UK primary care is high, some misclassification, particularly between T1D and T2D, is possible. The sensitivity analysis using the DDSC algorithm supports the main findings but highlights how outcome definitions can influence estimates. Residual confounding remains a concern: although we matched on key variables, unmeasured factors such as family history of diabetes, history of autoimmune disease, medication use, lifestyle and physical activity, history of other viral infections and repeated infections, vaccination status and COVID‐19 infection severity may have influenced the results. Separate analyses by BMI, deprivation, ethnicity and region were informative, but complex interactions among these variables could not be fully assessed due to computational constraints. Additionally, our study spans multiple pandemic phases, including different viral variants, vaccination rollouts and public health measures, which could not be separately assessed. Earlier waves were associated with more severe infection and increased healthcare contact, whereas later phases with vaccination and antiviral treatments may have impacted disease severity and exposure, potentially contributing to variation in observed associations over time. Lastly, BMI data were available for only around half the sample. Although complete case analysis still offered sufficient power, missing BMI data may introduce selection bias if BMI recording was associated with healthcare utilisation or underlying risk factors.

In conclusion, the effect of COVID‐19 was small in magnitude compared with the variation observed across established risk factors of T2D and may partly reflect detection bias. Diabetes prevention should continue to prioritise its well‐established metabolic and socioeconomic determinants, while further research is needed to identify potential high‐risk subgroups following COVID‐19.

## Author Contributions

**Sharmin Shabnam:** study design, data curation and cleaning, statistical analysis, writing manuscript. **Cameron Razieh:** study design, writing manuscript and critical revision for important intellectual content. **John Nolan:** study design, data curation and cleaning. **Francesco Zaccardi** and **Kamlesh Khunti:** conception, study design, statistical analysis, writing manuscript and critical revision for important intellectual content. All authors: interpretation of the data, critical review and final approval of the manuscript. **Kamlesh Khunti** and **Francesco Zaccardi** are the guarantors of this work and, as such, had full access to all the data in the study and take responsibility for the integrity of the data and the accuracy of the data analysis.

## Funding

The study was supported by the National Institute for Health Research (NIHR) Applied Research Collaboration East Midlands (ARC EM). The BHF DSC (grant No SP/19/3/34678, awarded to HDR UK) funded co‐development (with NHS England) of the Secure Data Environment (SDE) service for England, provision of linked datasets, data access, user software licences, computational usage and data management and wrangling support to coordinate national COVID‐19 priority research. Consortium partner organisations funded the time of contributing data analysts, biostatisticians, epidemiologists and clinicians.

## Ethics Statement

The North East—Newcastle and North Tyneside 2 research ethics committee provided ethical approval for the CVD‐COVID‐UK/COVID‐IMPACT research programme (REC No 20/NE/0161) to access, within secure trusted research environments, unconsented, whole‐population, de‐identified data from electronic health records collected as part of patients' routine healthcare.

## Conflicts of Interest

K.K. has been a consultant for Abbott, Amgen, AstraZeneca, Bayer, Boehringer Ingelheim, Lilly, Novo Nordisk, Sanofi, Servier, Pfizer, Roche, Daiichi‐Sankyo, Embecta and Nestle Health Science; has received research support from Abbott, AstraZeneca, Boehringer Ingelheim, Lilly, Merk, Novo Nordisk, Roche, Sanofi, Servier, Oramed Pharmaceuticals, Roche, Daiichi‐Sankyo and Applied Therapeutics; and was in a speaker's bureau for AstraZeneca, Bayer, Boehringer Ingelheim, Lilly, Merk, Novo Nordisk, Sanofi, Servier and Roche. The other authors declare no conflicts of interest.

## Supporting information


**Table S1:** Characteristics of the cohort in the BMI analyses, stratified by exposure status and sex.
**Table S2:** Number of events, total follow‐up time and incidence rates of diabetes outcomes in the BMI analyses, stratified by sex and COVID‐19 exposure status.
**Table S3:** Characteristics of the cohort in the sensitivity analysis (with outcomes defined by BHF DDSC diabetes phenotyping algorithm), stratified by exposure status and sex.
**Table S4:** Number of events, total follow‐up time and incidence rates from the cohort in the sensitivity analysis (with outcomes defined by BHF DDSC diabetes phenotyping algorithm), stratified by sex and COVID‐19 exposure status.
**Figure S1:** Study flow diagram.
**Figure S2:** Hazard rates of type 2 diabetes over time by body mass index, age and exposure status, with time‐varying effects of exposure and stratified by sex.
**Figure S3:** Hazard rates of type 2 diabetes over time by deprivation, age and exposure status, stratified by sex.
**Figure S4:** Hazard rates of type 2 diabetes over time by deprivation, age and exposure status, with time‐varying effects of exposure and stratified by sex.
**Figure S5:** Hazard rates of type 2 diabetes over time by ethnicity, age and exposure status, and stratified by sex.
**Figure S6:** Hazard rates of type 2 diabetes over time by ethnicity, age and exposure status, with time‐varying effects of exposure and stratified by sex.
**Figure S7:** Hazard rates of type 2 diabetes over time by region, age and exposure status, and stratified by sex.
**Figure S8:** Hazard rates of type 2 diabetes over time by region, age and exposure status, with time‐varying effects of exposure and stratified by sex.
**Figure S9:** Hazard rates of type 1 diabetes over time by body mass index, age and exposure status, with time‐varying effects of exposure and stratified by sex.
**Figure S10:** Hazard rates of type 1 diabetes over time by deprivation, age and exposure status, stratified by sex.
**Figure S11:** Hazard rates of type 1 diabetes over time by deprivation, age and exposure status, with time‐varying effects of exposure and stratified by sex.
**Figure S12:** Hazard rates of type 1 diabetes over time by ethnicity, age and exposure status, and stratified by sex.
**Figure S13:** Hazard rates of type 1 diabetes over time by ethnicity, age and exposure status, with time‐varying effects of exposure and stratified by sex.
**Figure S14:** Hazard rates of type 1 diabetes over time by region, age and exposure status, and stratified by sex.
**Figure S15:** Hazard rates of type 1 diabetes over time by region, age and exposure status, with time‐varying effects of exposure and stratified by sex.
**Figure S16:** Study flow diagram for the cohort with outcomes defined by BHF DDSC diabetes phenotyping algorithm.
**Figure S17:** Hazard rates of type 2 diabetes (defined by BHF DDSC diabetes phenotyping algorithm) over time by body mass index, age and exposure status, stratified by sex.
**Figure S18:** Hazard rates of type 2 diabetes (defined by BHF DDSC diabetes phenotyping algorithm) over time by body mass index, age and exposure status, with time‐varying effects of exposure and stratified by sex.
**Figure S19:** Hazard rates of type 2 diabetes (defined by BHF DDSC diabetes phenotyping algorithm) over time by deprivation, age and exposure status, stratified by sex.
**Figure S20:** Hazard rates of type 2 diabetes (defined by BHF DDSC diabetes phenotyping algorithm) over time by deprivation, age and exposure status, with time‐varying effects of exposure and stratified by sex.
**Figure S21:** Hazard rates of type 2 diabetes (defined by BHF DDSC diabetes phenotyping algorithm) over time by ethnicity, age and exposure status, and stratified by sex.
**Figure S22:** Hazard rates of type 2 diabetes (defined by BHF DDSC diabetes phenotyping algorithm) over time by ethnicity, age and exposure status, with time‐varying effects of exposure and stratified by sex.
**Figure S23:** Hazard rates of type 2 diabetes (defined by BHF DDSC diabetes phenotyping algorithm) over time by region, age and exposure status, and stratified by sex.
**Figure S24:** Hazard rates of type 2 diabetes (defined by BHF DDSC diabetes phenotyping algorithm) over time by region, age and exposure status, with time‐varying effects of exposure and stratified by sex.
**Figure S25:** Hazard rates of type 1 diabetes (defined by BHF DDSC diabetes phenotyping algorithm) over time by body mass index, age and exposure status, stratified by sex.
**Figure S26:** Hazard rates of type 1 diabetes (defined by BHF DDSC diabetes phenotyping algorithm) over time by body mass index, age and exposure status, with time‐varying effects of exposure and stratified by sex.
**Figure S27:** Hazard rates of type 1 diabetes (defined by BHF DDSC diabetes phenotyping algorithm) over time by deprivation, age and exposure status, stratified by sex.
**Figure S28:** Hazard rates of type 1 diabetes (defined by BHF DDSC diabetes phenotyping algorithm) over time by deprivation, age and exposure status, with time‐varying effects of exposure and stratified by sex.
**Figure S29:** Hazard rates of type 1 diabetes (defined by BHF DDSC diabetes phenotyping algorithm) over time by ethnicity, age and exposure status, and stratified by sex.
**Figure S30:** Hazard rates of type 1 diabetes (defined by BHF DDSC diabetes phenotyping algorithm) over time by ethnicity, age and exposure status, with time‐varying effects of exposure and stratified by sex.
**Figure S31:** Hazard rates of type 1 diabetes (defined by BHF DDSC diabetes phenotyping algorithm) over time by region, age and exposure status, and stratified by sex.
**Figure S32:** Hazard rates of type 1 diabetes (defined by BHF DDSC diabetes phenotyping algorithm) over time by region, age and exposure status, with time‐varying effects of exposure and stratified by sex.
**Figure S33:** Hazard rates of type 2 diabetes over time among hospitalised COVID‐19 patients and their matched controls by body mass index, age and exposure status, and stratified by sex.
**Figure S34:** Hazard rates of type 2 diabetes over time among hospitalised COVID‐19 patients and their matched controls by deprivation, age and exposure status, and stratified by sex.
**Figure S35:** Hazard rates of type 2 diabetes over time among hospitalised COVID‐19 patients and their matched controls by ethnicity, age and exposure status, and stratified by sex.
**Figure S36:** Hazard rates of type 2 diabetes over time among hospitalised COVID‐19 patients and their matched controls by region, age and exposure status, and stratified by sex.
**Figure S37:** Hazard rates of type 1 diabetes over time among hospitalised COVID‐19 patients and their matched controls by body mass index, age and exposure status, and stratified by sex.
**Figure S38:** Hazard rates of type 1 diabetes over time among hospitalised COVID‐19 patients and their matched controls by deprivation, age and exposure status, and stratified by sex.
**Figure S39:** Hazard rates of type 1 diabetes over time among hospitalised COVID‐19 patients and their matched controls by ethnicity, age and exposure status, and stratified by sex.
**Figure S40:** Hazard rates of type 1 diabetes over time among hospitalised COVID‐19 patients and their matched controls by region, age and exposure status, and stratified by sex.

## Data Availability

The analytical codes and phenotypes used within the NHS England SDE are available in the following GitHub repository: https://github.com/BHFDSC/CCU043_01. The data used in this study are available in NHS England's SDE service for England, but as restrictions apply, they are not publicly available (https://digital.nhs.uk/services/secure‐data‐environment‐service). The CVD‐COVID‐UK/COVID‐IMPACT programme, led by the BHF DSC (https://bhfdatasciencecentre.org/), received approval to access data in NHS England's SDE service for England from the Advisory Group for Data (AGD) (https://digital.nhs.uk/about‐nhs‐digital/corporate‐information‐and‐documents/advisory‐group‐for‐data) – formerly the Independent Group Advising on the Release of Data (IGARD) – via an application made in the Data Access Request Service (DARS) Online system (ref. DARS‐NIC‐381078‐Y9C5K) (https://digital.nhs.uk/services/data‐access‐request‐service‐dars/dars‐products‐and‐services). The CVD‐COVID‐UK/COVID‐IMPACT Approvals & Oversight Board (https://bhfdatasciencecentre.org/areas/cvd‐covid‐uk‐covid‐impact/) subsequently granted approval to this project (CCU043) to access the data within NHS England's SDE service for England. The de‐identified data used in this study were made available to accredited researchers only. Those wishing to gain access to the data should contact bhfdsc@hdruk.ac.uk in the first instance.
