# Supplementary material for: Variations in the Risk of New‐Onset Diabetes Following COVID‐19 Infection Across Body Mass Index, Deprivation, Ethnicity and Geographic Regions: Population‐Based Cohort Study in 42 Million People in England
Source: Diabetes Obes Metab. 2026 May 10;28(8):6661–73. doi: 10.1111/dom.70856 (PMC13341393; doi:10.1111/dom.70856)
Supplement: Supplementary file 1 — Table S1: Characteristics of the cohort in the BMI analyses, stratified by exposure status and sex. Table S2: Number of events, total follow‐up time and incidence rates of diabetes outcomes in the BMI analyses, stratified by sex and COVID‐19 exposure status. Table S3: Characteristics of the cohort in the sensitivity analysis (with outcomes defined by BHF DDSC diabetes phenotyping algorithm), stratified by exposure status and sex. Table S4: Number of events, total follow‐up time and incidence rates from the cohort in the sensitivity analysis (with outcomes defined by BHF DDSC diabetes phenotyping algorithm), stratified by sex and COVID‐19 exposure status. Figure S1: Study flow diagram. Figure S2: Hazard rates of type 2 diabetes over time by body mass index, age and exposure status, with time‐varying effects of exposure and stratified by sex. Figure S3: Hazard rates of type 2 diabetes over time by deprivation, age and exposure status, stratified by sex. Figure S4: Hazard rates of type 2 diabetes over time by deprivation, age and exposure status, with time‐varying effects of exposure and stratified by sex. Figure S5: Hazard rates of type 2 diabetes over time by ethnicity, age and exposure status, and stratified by sex. Figure S6: Hazard rates of type 2 diabetes over time by ethnicity, age and exposure status, with time‐varying effects of exposure and stratified by sex. Figure S7: Hazard rates of type 2 diabetes over time by region, age and exposure status, and stratified by sex. Figure S8: Hazard rates of type 2 diabetes over time by region, age and exposure status, with time‐varying effects of exposure and stratified by sex. Figure S9: Hazard rates of type 1 diabetes over time by body mass index, age and exposure status, with time‐varying effects of exposure and stratified by sex. Figure S10: Hazard rates of type 1 diabetes over time by deprivation, age and exposure status, stratified by sex. Figure S11: Hazard rates of type 1 diabetes over time by deprivation, age an [file DOM-28-6661-s001.docx]

**SUPPLEMENTARY MATERIAL**

**Variations across body mass index, deprivation, ethnicity, and region in the risk of new-onset diabetes following COVID-19 infection: Population-based cohort study in 42 million people in England**

Sharmin Shabnam, Cameron Razieh, John Nolan, Nazrul Islam, Genevieve Cezard, Yogini V Chudasama, Clare L Gillies, Amitava Banerjee, Angela Wood, Kamlesh Khunti, Francesco Zaccardi

On behalf of the CVD-COVID-UK/COVID-IMPACT Consortium

Contents

[Table S1: Summary of results 4](#_Toc228440177)

[Table S2: Characteristics of the cohort in the BMI analyses, stratified by exposure status and sex 5](#_Toc228440178)

[Table S3: Number of events, total follow-up time, and incidence rates of diabetes outcomes in the BMI analyses, stratified by sex and COVID-19 exposure status 7](#_Toc228440179)

[Table S4: Characteristics of the cohort in the sensitivity analysis (with outcomes defined by BHF DDSC diabetes phenotyping algorithm), stratified by exposure status and sex 8](#_Toc228440180)

[Table S5: Number of events, total follow-up time, and incidence rates from the cohort in the sensitivity analysis (with outcomes defined by BHF DDSC diabetes phenotyping algorithm), stratified by sex and COVID-19 exposure status 10](#_Toc228440181)

[Figure S1: Study flow diagram 11](#_Toc228440182)

[Figure S2: Hazard rates of type 2 diabetes over time by body mass index, age, and exposure status, with time-varying effects of exposure and stratified by sex 12](#_Toc228440183)

[Figure S3: Hazard rates of type 2 diabetes over time by deprivation, age, and exposure status, stratified by sex 13](#_Toc228440184)

[Figure S4: Hazard rates of type 2 diabetes over time by deprivation, age, and exposure status, with time-varying effects of exposure and stratified by sex 14](#_Toc228440185)

[Figure S5: Hazard rates of type 2 diabetes over time by ethnicity, age, and exposure status, and stratified by sex 15](#_Toc228440186)

[Figure S6: Hazard rates of type 2 diabetes over time by ethnicity, age, and exposure status, with time-varying effects of exposure and stratified by sex 16](#_Toc228440187)

[Figure S7: Hazard rates of type 2 diabetes over time by region, age, and exposure status, and stratified by sex 17](#_Toc228440188)

[Figure S8: Hazard rates of type 2 diabetes over time by region, age, and exposure status, with time-varying effects of exposure and stratified by sex 18](#_Toc228440189)

[Figure S9: Hazard rates of type 1 diabetes over time by body mass index, age, and exposure status, with time-varying effects of exposure and stratified by sex 19](#_Toc228440190)

[Figure S10: Hazard rates of type 1 diabetes over time by deprivation, age, and exposure status, stratified by sex 20](#_Toc228440191)

[Figure S11: Hazard rates of type 1 diabetes over time by deprivation, age, and exposure status, with time-varying effects of exposure and stratified by sex 21](#_Toc228440192)

[Figure S12: Hazard rates of type 1 diabetes over time by ethnicity, age, and exposure status, and stratified by sex 22](#_Toc228440193)

[Figure S13: Hazard rates of type 1 diabetes over time by ethnicity, age, and exposure status, with time-varying effects of exposure and stratified by sex 23](#_Toc228440194)

[Figure S14: Hazard rates of type 1 diabetes over time by region, age, and exposure status, and stratified by sex 24](#_Toc228440195)

[Figure S15: Hazard rates of type 1 diabetes over time by region, age, and exposure status, with time-varying effects of exposure and stratified by sex 25](#_Toc228440196)

[Figure S16: Study flow diagram for the cohort with outcomes defined by BHF DDSC diabetes phenotyping algorithm 26](#_Toc228440197)

[Figure S17: Hazard rates of type 2 diabetes (defined by BHF DDSC diabetes phenotyping algorithm) over time by body mass index, age, and exposure status, stratified by sex 27](#_Toc228440198)

[Figure S18: Hazard rates of type 2 diabetes (defined by BHF DDSC diabetes phenotyping algorithm) over time by body mass index, age, and exposure status, with time-varying effects of exposure and stratified by sex 28](#_Toc228440199)

[Figure S19: Hazard rates of type 2 diabetes (defined by BHF DDSC diabetes phenotyping algorithm) over time by deprivation, age, and exposure status, stratified by sex 29](#_Toc228440200)

[Figure S20: Hazard rates of type 2 diabetes (defined by BHF DDSC diabetes phenotyping algorithm) over time by deprivation, age, and exposure status, with time-varying effects of exposure and stratified by sex 30](#_Toc228440201)

[Figure S21: Hazard rates of type 2 diabetes (defined by BHF DDSC diabetes phenotyping algorithm) over time by ethnicity, age, and exposure status, and stratified by sex 31](#_Toc228440202)

[Figure S22: Hazard rates of type 2 diabetes (defined by BHF DDSC diabetes phenotyping algorithm) over time by ethnicity, age, and exposure status, with time-varying effects of exposure and stratified by sex 32](#_Toc228440203)

[Figure S23: Hazard rates of type 2 diabetes (defined by BHF DDSC diabetes phenotyping algorithm) over time by region, age, and exposure status, and stratified by sex 33](#_Toc228440204)

[Figure S24: Hazard rates of type 2 diabetes (defined by BHF DDSC diabetes phenotyping algorithm) over time by region, age, and exposure status, with time-varying effects of exposure and stratified by sex 34](#_Toc228440205)

[Figure S25: Hazard rates of type 1 diabetes (defined by BHF DDSC diabetes phenotyping algorithm) over time by body mass index, age, and exposure status, stratified by sex 35](#_Toc228440206)

[Figure S26: Hazard rates of type 1 diabetes (defined by BHF DDSC diabetes phenotyping algorithm) over time by body mass index, age, and exposure status, with time-varying effects of exposure and stratified by sex 36](#_Toc228440207)

[Figure S27: Hazard rates of type 1 diabetes (defined by BHF DDSC diabetes phenotyping algorithm) over time by deprivation, age, and exposure status, stratified by sex 37](#_Toc228440208)

[Figure S28: Hazard rates of type 1 diabetes (defined by BHF DDSC diabetes phenotyping algorithm) over time by deprivation, age, and exposure status, with time-varying effects of exposure and stratified by sex 38](#_Toc228440209)

[Figure S29: Hazard rates of type 1 diabetes (defined by BHF DDSC diabetes phenotyping algorithm) over time by ethnicity, age, and exposure status, and stratified by sex 39](#_Toc228440210)

[Figure S30: Hazard rates of type 1 diabetes (defined by BHF DDSC diabetes phenotyping algorithm) over time by ethnicity, age, and exposure status, with time-varying effects of exposure and stratified by sex 40](#_Toc228440211)

[Figure S31: Hazard rates of type 1 diabetes (defined by BHF DDSC diabetes phenotyping algorithm) over time by region, age, and exposure status, and stratified by sex 41](#_Toc228440212)

[Figure S32: Hazard rates of type 1 diabetes (defined by BHF DDSC diabetes phenotyping algorithm) over time by region, age, and exposure status, with time-varying effects of exposure and stratified by sex 42](#_Toc228440213)

[Figure S33: Hazard rates of type 2 diabetes over time among hospitalised COVID-19 patients and their matched controls by body mass index, age, and exposure status, and stratified by sex 43](#_Toc228440214)

[Figure S34: Hazard rates of type 2 diabetes over time among hospitalised COVID-19 patients and their matched controls by deprivation, age, and exposure status, and stratified by sex 44](#_Toc228440215)

[Figure S35: Hazard rates of type 2 diabetes over time among hospitalised COVID-19 patients and their matched controls by ethnicity, age, and exposure status, and stratified by sex 45](#_Toc228440216)

[Figure S36: Hazard rates of type 2 diabetes over time among hospitalised COVID-19 patients and their matched controls by region, age, and exposure status, and stratified by sex 46](#_Toc228440217)

[Figure S37: Hazard rates of type 1 diabetes over time among hospitalised COVID-19 patients and their matched controls by body mass index, age, and exposure status, and stratified by sex 47](#_Toc228440218)

[Figure S38: Hazard rates of type 1 diabetes over time among hospitalised COVID-19 patients and their matched controls by deprivation, age, and exposure status, and stratified by sex 48](#_Toc228440219)

[Figure S39: Hazard rates of type 1 diabetes over time among hospitalised COVID-19 patients and their matched controls by ethnicity, age, and exposure status, and stratified by sex 49](#_Toc228440220)

[Figure S40: Hazard rates of type 1 diabetes over time among hospitalised COVID-19 patients and their matched controls by region, age, and exposure status, and stratified by sex 50](#_Toc228440221)

[The RECORD statement: Checklist of items, extended from the STROBE statement, that should be reported in observational studies using routinely collected health data. 51](#_Toc228440222)

# Table S1: Summary of results

| Diabetes Type | DDSC Algorithm | Cohort | Level | Time-varying | Figure number |
| --- | --- | --- | --- | --- | --- |
| 2 | N | Full | Body Mass Index | N | 2 |
| 2 | N |  | Body Mass Index | Y | S2 |
| 2 | N |  | Deprivation | N | S3 |
| 2 | N |  | Deprivation | Y | S4 |
| 2 | N |  | Ethnicity | N | S5 |
| 2 | N |  | Ethnicity | Y | S6 |
| 2 | N |  | Region | N | S7 |
| 2 | N |  | Region | Y | S8 |
| 2 | Y | Outcomes defined by Diabetes Data Science Catalyst diabetes phenotype algorithm | Body Mass Index | N | S17 |
| 2 | Y |  | Body Mass Index | Y | S18 |
| 2 | Y |  | Deprivation | N | S19 |
| 2 | Y |  | Deprivation | Y | S20 |
| 2 | Y |  | Ethnicity | N | S21 |
| 2 | Y |  | Ethnicity | Y | S22 |
| 2 | Y |  | Region | N | S23 |
| 2 | Y |  | Region | Y | S24 |
| 1 | N | Full | Body Mass Index | N | 3 |
| 1 | N |  | Body Mass Index | Y | S9 |
| 1 | N |  | Deprivation | N | S10 |
| 1 | N |  | Deprivation | Y | S11 |
| 1 | N |  | Ethnicity | N | S12 |
| 1 | N |  | Ethnicity | Y | S13 |
| 1 | N |  | Region | N | S14 |
| 1 | N |  | Region | Y | S15 |
| 1 | Y | Outcomes defined by Diabetes Data Science Catalyst diabetes phenotype algorithm | Body Mass Index | N | S25 |
| 1 | Y |  | Body Mass Index | Y | S26 |
| 1 | Y |  | Deprivation | N | S27 |
| 1 | Y |  | Deprivation | Y | S28 |
| 1 | Y |  | Ethnicity | N | S29 |
| 1 | Y |  | Ethnicity | Y | S30 |
| 1 | Y |  | Region | N | S31 |
| 1 | Y |  | Region | Y | S32 |
| 2 | N | Restricted to hospitalised COVID-19 cases and their matched controls | Body Mass Index | N | S33 |
| 2 | N |  | Deprivation | N | S34 |
| 2 | N |  | Ethnicity | N | S35 |
| 2 | N |  | Region | N | S36 |
| 1 | N |  | Body Mass Index | N | S37 |
| 1 | N |  | Deprivation | N | S38 |
| 1 | N |  | Ethnicity | N | S39 |
| 1 | N |  | Region | N | S40 |

# Table S2: Characteristics of the cohort in the BMI analyses, stratified by exposure status and sex

| Category | Unexposed (no COVID-19) | | | Exposed (COVID-19) | | |
| --- | --- | --- | --- | --- | --- | --- |
|  | **Overall**  **N = 14,360,905** | **Women**  **N = 8,921,215 (62.1%)** | **Men**  **N = 5,439,690 (37.9%)** | **Overall**  **N = 7,447,950** | **Women**  **N = 4,706,200 (63.2%)** | **Men**  **N = 2,741,745 (36.8%)** |
| Age (years), mean (SD) | 47.5 (17.6) | 46.3 (17.7) | 49.5 (17.4) | 47.9 (18.0) | 46.1 (17.9) | 51.0 (17.7) |
| Age (years), n (%) |  |  |  |  |  |  |
| 18-29 | 2,678,380 (18.7) | 1,860,150 (20.9) | 818,230 (15.0) | 1,339,240 (18.0) | 982,895 (20.9) | 356,340 (13.0) |
| 30-39 | 2,710,345 (18.9) | 1,791,595 (20.1) | 918,750 (16.9) | 1,415,195 (19.0) | 973,135 (20.7) | 442,060 (16.1) |
| 40-49 | 2,631,650 (18.3) | 1,585,715 (17.8) | 1,045,935 (19.2) | 1,418,010 (19.0) | 890,910 (18.9) | 527,100 (19.2) |
| 50-59 | 2,766,800 (19.3) | 1,669,030 (18.7) | 1,097,770 (20.2) | 1,381,450 (18.5) | 828,535 (17.6) | 552,910 (20.2) |
| 60-69 | 1,768,845 (12.3) | 996,290 (11.2) | 772,555 (14.2) | 888,445 (11.9) | 482,300 (10.2) | 406,145 (14.8) |
| 70-79 | 1,095,825 (7.6) | 588,510 (6.6) | 507,315 (9.3) | 565,995 (7.6) | 288,700 (6.1) | 277,295 (10.1) |
| 80+ | 709,060 (4.9) | 429,925 (4.8) | 279,135 (5.1) | 439,615 (5.9) | 259,720 (5.5) | 179,895 (6.6) |
| Ethnicity, n (%) |  |  |  |  |  |  |
| White | 11,548,235 (80.4) | 7,212,090 (80.8) | 4,336,145 (79.7) | 6,371,995 (85.6) | 4,052,140 (86.1) | 2,319,855 (84.6) |
| Asian or Asian British | 1,524,930 (10.6) | 918,385 (10.3) | 606,545 (11.2) | 580,265 (7.8) | 341,940 (7.3) | 238,325 (8.7) |
| Black or Black British | 590,170 (4.1) | 368,970 (4.1) | 221,205 (4.1) | 225,620 (3.0) | 143,630 (3.1) | 81,990 (3.0) |
| Mixed/Other | 583,775 (4.1) | 361,235 (4.0) | 222,540 (4.1) | 241,390 (3.2) | 153,395 (3.3) | 87,995 (3.2) |
| Missing | 113,795 (0.8) | 60,540 (0.7) | 53,255 (1.0) | 28,670 (0.4) | 15,095 (0.3) | 13,575 (0.5) |
| IMD (quintiles), n (%) |  |  |  |  |  |  |
| 1 (Most deprived) | 2,697,310 (18.8) | 1,724,395 (19.3) | 972,915 (17.9) | 1,404,765 (18.9) | 905,285 (19.2) | 499,480 (18.2) |
| 2 | 2,886,360 (20.1) | 1,810,860 (20.3) | 1,075,500 (19.8) | 1,511,865 (20.3) | 960,545 (20.4) | 551,320 (20.1) |
| 3 | 2,937,515 (20.5) | 1,823,620 (20.4) | 1,113,895 (20.5) | 1,523,880 (20.5) | 961,975 (20.4) | 561,910 (20.5) |
| 4 | 2,935,035 (20.4) | 1,802,585 (20.2) | 1,132,450 (20.8) | 1,519,730 (20.4) | 954,100 (20.3) | 565,630 (20.6) |
| 5 (Least deprived) | 2,904,680 (20.2) | 1,759,750 (19.7) | 1,144,930 (21.0) | 1,487,705 (20.0) | 924,305 (19.6) | 563,405 (20.5) |
| Region, n (%) |  |  |  |  |  |  |
| East Midlands | 1,203,005 (8.4) | 751,635 (8.4) | 451,370 (8.3) | 625,675 (8.4) | 398,480 (8.5) | 227,200 (8.3) |
| East of England | 1,597,330 (11.1) | 996,380 (11.2) | 600,950 (11.0) | 816,525 (11.0) | 517,210 (11.0) | 299,315 (10.9) |
| London | 2,150,295 (15.0) | 1,333,580 (14.9) | 816,715 (15.0) | 1,129,690 (15.2) | 697,125 (14.8) | 432,565 (15.8) |
| North East | 732,565 (5.1) | 452,645 (5.1) | 279,920 (5.1) | 396,205 (5.3) | 254,830 (5.4) | 141,375 (5.2) |
| North West | 1,971,315 (13.7) | 1,214,620 (13.6) | 756,695 (13.9) | 1,032,480 (13.9) | 653,205 (13.9) | 379,270 (13.8) |
| South East | 2,356,040 (16.4) | 1,469,080 (16.5) | 886,960 (16.3) | 1,189,475 (16.0) | 750,290 (15.9) | 439,180 (16.0) |
| South West | 1,422,590 (9.9) | 883,410 (9.9) | 539,180 (9.9) | 710,735 (9.5) | 451,025 (9.6) | 259,705 (9.5) |
| West Midlands | 1,500,905 (10.5) | 930,805 (10.4) | 570,100 (10.5) | 799,310 (10.7) | 506,755 (10.8) | 292,555 (10.7) |
| Yorkshire and The Humber | 1,426,855 (9.9) | 889,065 (10.0) | 537,795 (9.9) | 747,860 (10.0) | 477,275 (10.1) | 270,585 (9.9) |
| Smoking status, n (%) |  |  |  |  |  |  |
| No | 6,725,455 (46.8) | 4,514,500 (50.6) | 2,210,955 (40.6) | 3,250,080 (43.6) | 2,159,500 (45.9) | 1,090,575 (39.8) |
| Yes | 2,022,525 (14.1) | 1,084,850 (12.2) | 937,675 (17.2) | 722,435 (9.7) | 424,380 (9.0) | 298,055 (10.9) |
| Ex | 2,752,080 (19.2) | 1,519,150 (17.0) | 1,232,930 (22.7) | 1,540,225 (20.7) | 860,750 (18.3) | 679,470 (24.8) |
| Missing | 2,860,845 (19.9) | 1,802,715 (20.2) | 1,058,130 (19.5) | 1,935,210 (26.0) | 1,261,570 (26.8) | 673,640 (24.6) |
| BMI categories (Kg/m^2^), n (%) |  |  |  |  |  |  |
| Underweight [<18.5] | 400,085 (2.8) | 305,935 (3.4) | 94,145 (1.7) | 187,700 (2.5) | 143,110 (3.0) | 44,590 (1.6) |
| Normal [18.5-24.9] | 5,150,670 (35.9) | 3,451,385 (38.7) | 1,699,280 (31.2) | 2,524,825 (33.9) | 1,735,645 (36.9) | 789,180 (28.8) |
| Overweight [25-29.9] | 4,684,455 (32.6) | 2,551,740 (28.6) | 2,132,715 (39.2) | 2,429,885 (32.6) | 1,348,050 (28.6) | 1,081,835 (39.5) |
| Obesity [30-39.9] | 3,455,755 (24.1) | 2,115,430 (23.7) | 1,340,330 (24.6) | 1,911,405 (25.7) | 1,182,555 (25.1) | 728,850 (26.6) |
| Severe obesity [≥40] | 669,940 (4.7) | 496,725 (5.6) | 173,220 (3.2) | 394,130 (5.3) | 296,840 (6.3) | 97,290 (3.5) |
| BMI values (Kg/m^2^) |  |  |  |  |  |  |
| Mean (SD) | 27.6 (6.5) | 27.5 (7.0) | 27.7 (5.8) | 28.0 (6.7) | 27.9 (7.1) | 28.1 (5.9) |
| Median [IQR] | 26.5 [23.1, 30.8] | 26.1 [22.6, 31.0] | 26.9 [24.0, 30.4] | 26.8 [23.4, 31.2] | 26.5 [22.9, 31.5] | 27.2 [24.3, 30.8] |

**Note:** Numbers are rounded to the nearest five as per NHS Data Access Environment (DAE) safe output guidelines.

SD = standard deviation; IQR = interquartile range; IMD = index of multiple deprivation; BMI: Body Mass Index.

# Table S3: Number of events, total follow-up time, and incidence rates of diabetes outcomes in the BMI analyses, stratified by sex and COVID-19 exposure status

| **Sex** | **Exposure** | **Events** | **Person-years** | **Median [IQR] follow up (years)** | **Crude incidence rate (per 100 000 person-years)** | **Age-standardised incidence rate (per 100 000 person-years)** |
| --- | --- | --- | --- | --- | --- | --- |
| **Type 2 Diabetes** | | | | | | |
| Men | Unexposed | 141,245 | 11,906,385 | 2.4 [1.8, 2.8] | 1,186 | 1,120 |
|  | Exposed | 83,545 | 6,674,840 | 2.4 [2.2, 2.8] | 1,252 | 1,166 |
| Women | Unexposed | 121,705 | 18,691,735 | 2.3 [1.5, 2.7] | 651 | 680 |
|  | Exposed | 71,100 | 11,759,200 | 2.4 [2.2, 2.9] | 605 | 677 |
| **Type 1 Diabetes** | | | | | | |
| Men | Unexposed | 2,685 | 12,095,830 | 2.4 [1.9, 2.8] | 22 | 24 |
|  | Exposed | 1,390 | 6,795,760 | 2.4 [2.2, 2.9] | 20 | 22 |
| Women | Unexposed | 2,140 | 18,853,210 | 2.3 [1.6, 2.7] | 11 | 11 |
|  | Exposed | 1,085 | 11,860,680 | 2.4 [2.2, 2.9] | 9 | 9 |

“Exposed” group consisted of individuals who had at least one recorded diagnosis of COVID-19 during the study period and did not have any record of prevalent diabetes. “Unexposed” refers to individuals with no record of diabetes or COVID-19 diagnosis on or before the matched index date. Crude incidence rates were calculated as the number of events divided by total person-years, multiplied by 100,000. Age-standardised incidence rates were calculated using standardisation to the unexposed cohort to account for differences in age distribution between groups.

**Note:** Numbers are rounded to the nearest five as per NHS Data Access Environment (DAE) safe output guidelines.

IQR = interquartile range.

# Table S4: Characteristics of the cohort in the sensitivity analysis (with outcomes defined by BHF DDSC diabetes phenotyping algorithm), stratified by exposure status and sex

| Category | Unexposed (no COVID-19) | | | Exposed (COVID-19) | | |
| --- | --- | --- | --- | --- | --- | --- |
|  | **Overall**  **N = 29,209,400** | **Women**  **N = 15,603,125 (53.4%)** | **Men**  **N = 13,606,275 (46.6%)** | **Overall**  **N = 12,853,490** | **Women**  **N = 7,161,155 (55.7%)** | **Men**  **N = 5,692,335 (44.3%)** |
| Age (years), mean (SD) | 44.5 (16.8) | 44.9 (17.1) | 44.0 (16.3) | 45.5 (17.3) | 45.4 (17.3) | 45.6 (17.1) |
| Age (years), n (%) |  |  |  |  |  |  |
| 18-29 | 6,428,125 (22.0) | 3,469,495 (22.2) | 2,958,630 (21.7) | 2,631,490 (20.5) | 1,483,660 (20.7) | 1,147,830 (20.2) |
| 30-39 | 6,679,140 (22.9) | 3,468,465 (22.2) | 3,210,675 (23.6) | 2,853255 (22.2) | 1,596,190 (22.3) | 1,257,065 (22.1) |
| 40-49 | 5,563,915 (19.0) | 2,826,915 (18.1) | 2,737,000 (20.1) | 2,532,640 (19.7) | 1,411,545 (19.7) | 1,121,095 (19.7) |
| 50-59 | 5,020,990 (17.2) | 2,748,660 (17.6) | 2,272,335 (16.7) | 2,207,080 (17.2) | 1,235,990 (17.3) | 971,090 (17.1) |
| 60-69 | 2,888,835 (9.9) | 1,598,955 (10.2) | 1,289,880 (9.5) | 1,291,920 (10.1) | 702,835 (9.8) | 589,090 (10.3) |
| 70-79 | 1,618,640 (5.5) | 874,665 (5.6) | 743,975 (5.5) | 757,470 (5.9) | 390,265 (5.4) | 367,205 (6.5) |
| 80+ | 1,009,755 (3.5) | 615,970 (3.9) | 393,785 (2.9) | 579,635 (4.5) | 340,675 (4.8) | 238,960 (4.2) |
| Ethnicity, n (%) |  |  |  |  |  |  |
| White | 22,120,315 (75.7) | 11,954,080 (76.6) | 10,166,235 (74.7) | 10,880,160 (84.6) | 6,105,995 (85.3) | 4,774,160 (83.9) |
| Asian or Asian British | 3,411,260 (11.7) | 1,830,830 (11.7) | 1,580,430 (11.6) | 1,014,445 (7.9) | 546,940 (7.6) | 467,505 (8.2) |
| Black or Black British | 1,254,105 (4.3) | 695,405 (4.5) | 558,695 (4.1) | 385,200 (3.0) | 219,770 (3.1) | 165,430 (2.9) |
| Mixed/Other | 1,361,105 (4.7) | 723,760 (4.6) | 637,345 (4.7) | 436,755 (3.4) | 242,515 (3.4) | 194,235 (3.4) |
| Missing | 1,062,615 (3.6) | 399,050 (2.6) | 663,570 (4.9) | 136,935 (1.1) | 45,935 (0.6) | 91,005 (1.6) |
| IMD (quintiles), n (%) |  |  |  |  |  |  |
| 1 (Most deprived) | 5,553,085 (19.0) | 3,017,405 (19.3) | 2,535,685 (18.6) | 2,407,335 (18.7) | 1,355,670 (18.9) | 1,051,670 (18.5) |
| 2 | 6,003,810 (20.6) | 3,228,020 (20.7) | 2,775,790 (20.4) | 2,597,355 (20.2) | 1,448,850 (20.2) | 1,148,500 (20.2) |
| 3 | 5,965,420 (20.4) | 3,192,145 (20.5) | 2,773,275 (20.4) | 2,619,060 (20.4) | 1,461,620 (20.4) | 1,157,440 (20.3) |
| 4 | 5,887,010 (20.2) | 3,117,630 (20.0) | 2,769,380 (20.4) | 2,627,690 (20.4) | 1,461,320 (20.4) | 1,166,370 (20.5) |
| 5 (Least deprived) | 5,800,070 (19.9) | 3,047,920 (19.5) | 2,752,150 (20.2) | 2,602,050 (20.2) | 1,433,695 (20.0) | 1,168,360 (20.5) |
| Region, n (%) |  |  |  |  |  |  |
| East Midlands | 2,464,455 (8.4) | 1,308,980 (8.4) | 1,155,480 (8.5) | 1,101,095 (8.6) | 613,630 (8.6) | 487,460 (8.6) |
| East of England | 3,187,940 (10.9) | 1,711,160 (11.0) | 1,476,780 (10.9) | 1,399,605 (10.9) | 781,945 (10.9) | 617,655 (10.9) |
| London | 4,796,535 (16.4) | 2,579,070 (16.5) | 2,217,460 (16.3) | 1,970,480 (15.3) | 1,079,170 (15.1) | 891,310 (15.7) |
| North East | 1,403,155 (4.8) | 729,680 (4.7) | 673,470 (4.9) | 663,010 (5.2) | 370,725 (5.2) | 292,285 (5.1) |
| North West | 3,880,015 (13.3) | 2,039,205 (13.1) | 1,840,815 (13.5) | 1,760,025 (13.7) | 977,190 (13.6) | 782,835 (13.8) |
| South East | 4,814,930 (16.5) | 2,600,885 (16.7) | 2,214,045 (16.3) | 2,087,240 (16.2) | 1,164,450 (16.3) | 922,790 (16.2) |
| South West | 2,793,220 (9.6) | 1,517,065 (9.7) | 1,276,155 (9.4) | 1,227,720 (9.6) | 695,285 (9.7) | 532,435 (9.4) |
| West Midlands | 3,038,635 (10.4) | 1,611,635 (10.3) | 1,427,000 (10.5) | 1,370,920 (10.7) | 765,180 (10.7) | 605,740 (10.6) |
| Yorkshire and The Humber | 2,830,515 (9.7) | 1,505,440 (9.6) | 1,325,075 (9.7) | 1,273,400 (9.9) | 713,580 (10.0) | 559,815 (9.8) |
| Smoking status, n (%) |  |  |  |  |  |  |
| No | 9,101,630 (31.2) | 5,972,785 (38.3) | 3,128,845 (23.0) | 4,255,270 (33.1) | 2,762,500 (38.6) | 1,492,770 (26.2) |
| Yes | 3,075,165 (10.5) | 1,527,055 (9.8) | 1,548,110 (11.4) | 1,063,600 (8.3) | 581,770 (8.1) | 481,825 (8.5) |
| Ex | 3,669,365 (12.6) | 1,978,760 (12.7) | 1,690,605 (12.4) | 2,001,330 (15.6) | 1,096,190 (15.3) | 905,135 (15.9) |
| Missing | 13,363,235 (45.7) | 6,124,520 (39.3) | 7,238,715 (53.2) | 5,533,295 (43.0) | 2,720,695 (38.0) | 2,812,605 (49.4) |
| BMI categories (Kg/m^2^), n (%) |  |  |  |  |  |  |
| Underweight [<18.5] | 400,030 (1.4) | 305,900 (2.0) | 94,130 (0.7) | 187,675 (1.5) | 143,095 (2.0) | 44,580 (0.8) |
| Normal [18.5-24.9] | 5,149,665 (17.6) | 3,450,935 (22.1) | 1,698,730 (12.5) | 2,524,295 (19.6) | 1,735,410 (24.2) | 788,890 (13.9) |
| Overweight [25-29.9] | 4,681,380 (16.0) | 2,550,535 (16.3) | 2,130,845 (15.7) | 2,428,425 (18.9) | 1,347,495 (18.8) | 1,080,930 (19.0) |
| Obesity [30-39.9] | 3,450,570 (11.8) | 2,113,050 (13.5) | 1,337,520 (9.8) | 1,908,680 (14.8) | 1,181,315 (16.5) | 727,365 (12.8) |
| Severe obesity [≥40] | 668,190 (2.3) | 495,615 (3.2) | 172,575 (1.3) | 393,200 (3.1) | 296,265 (4.1) | 96,935 (1.7) |
| BMI values (Kg/m^2^) |  |  |  |  |  |  |
| Mean (SD) | 27.6 (6.5) | 27.5 (7.0) | 27.7 (5.8) | 28.0 (6.7) | 27.9 (7.1) | 28.1 (5.9) |
| Median [IQR] | 26.5 [23.1, 30.7] | 26.1 [22.6, 31.0] | 26.9 [24.0, 30.4] | 26.8 [23.4, 31.2] | 26.5 [22.9, 31.5] | 27.2 [24.3, 30.8] |
| Missing, n (%) | 14,859,565 (50.9) | 6,687,090 (42.9) | 8,172,475 (60.1) | 5,411,210 (42.1) | 2,457,575 (34.3) | 2,953,635 (51.9) |

**Note:** Numbers are rounded to the nearest five as per NHS Data Access Environment (DAE) safe output guidelines.

SD = standard deviation; IQR = interquartile range; IMD = index of multiple deprivation; BMI: Body Mass Index; BHF: British Heart Foundation; DDSC: Diabetes Data Science Catalyst.

# Table S5: Number of events, total follow-up time, and incidence rates from the cohort in the sensitivity analysis (with outcomes defined by BHF DDSC diabetes phenotyping algorithm), stratified by sex and COVID-19 exposure status

| **Sex** | **Exposure** | **Events** | **Person-years** | **Median [IQR] follow up (years)** | **Crude incidence rate (per 100 000 person-years)** | **Age-standardised incidence rate (per 100 000 person-years)** |  |
| --- | --- | --- | --- | --- | --- | --- | --- |
| **Type 2 Diabetes** | | | | | | | |
| Men | Unexposed | 156,880 | 31,115,840 | 2.4 [2.1, 2.8] | 504 | 517 |  |
|  | Exposed | 90,650 | 14,335,075 | 2.4 [2.2, 2.9] | 632 | 632 |  |
| Women | Unexposed | 133,780 | 33,792,460 | 2.4 [1.8, 2.7] | 396 | 388 |  |
|  | Exposed | 75,205 | 17,979,145 | 2.4 [2.2, 2.9] | 418 | 422 |  |
| **Type 1 Diabetes** | | | | | | | |
| Men | Unexposed | 4,095 | 31,333,115 | 2.4 [2.1, 2.8] | 13 | 13 |  |
|  | Exposed | 1,960 | 14,472,540 | 2.4 [2.3, 2.9] | 14 | 14 |  |
| Women | Unexposed | 3,195 | 33,977,005 | 2.4 [1.8, 2.7] | 9 | 9 |  |
|  | Exposed | 1,595 | 18,092,315 | 2.4 [2.2, 2.9] | 9 | 9 |  |

“Exposed” group consisted of individuals who had at least one recorded diagnosis of COVID-19 during the study period and did not have any record of prevalent diabetes. “Unexposed” refers to individuals with no record of diabetes or COVID-19 diagnosis on or before the matched index date. Crude incidence rates were calculated as the number of events divided by total person-years, multiplied by 100,000. Age-standardised incidence rates were calculated using standardisation to the unexposed cohort to account for differences in age distribution between groups.

**Note:** Numbers are rounded to the nearest five as per NHS Data Access Environment (DAE) safe output guidelines.

IQR = interquartile range; BHF: British Heart Foundation; DDSC: Diabetes Data Science Catalyst.

# Figure S1: Study flow diagram


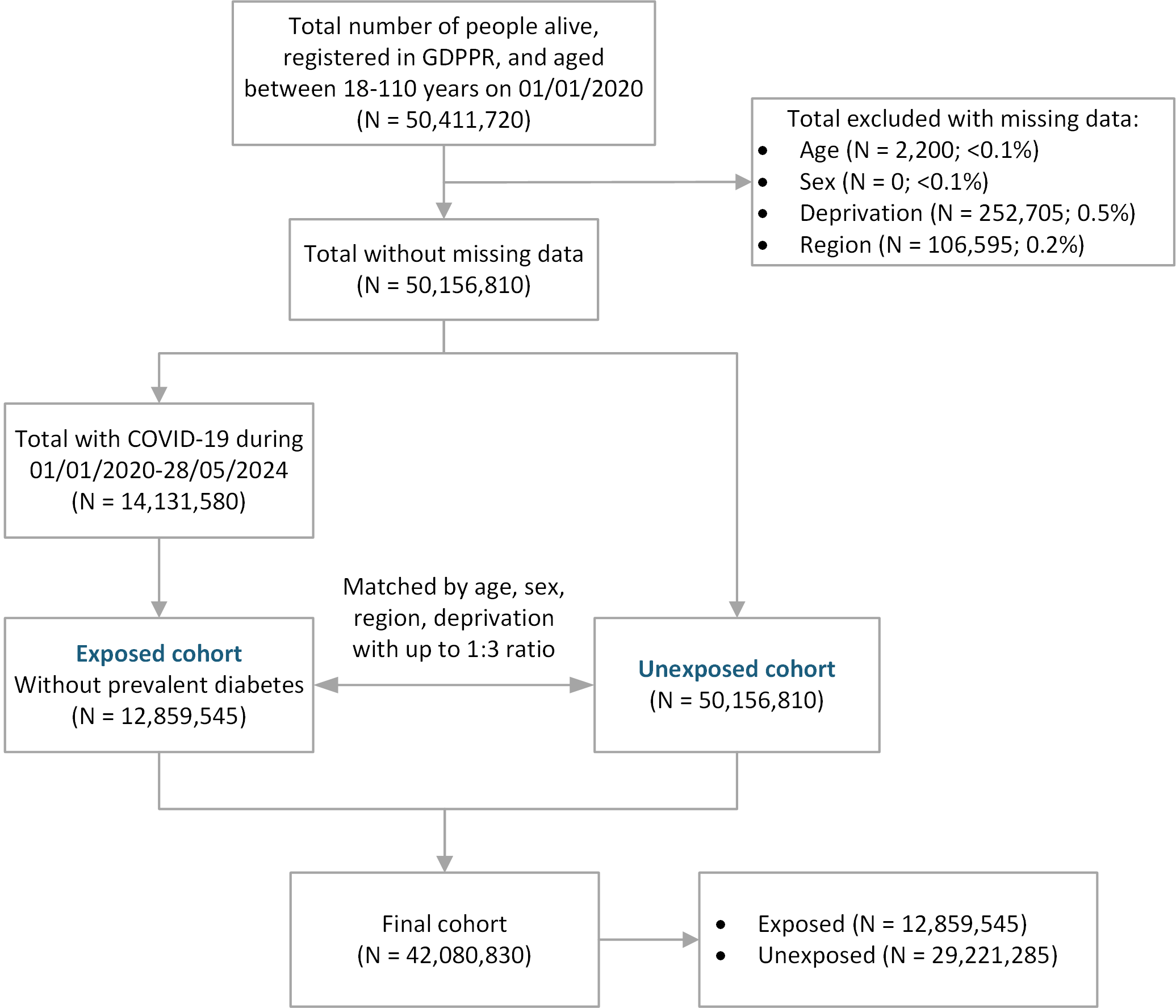


Prevalent diabetes is defined as having any record of diabetes in primary or secondary care before or at the earliest date of COVID-19 (for the exposed) or the matched index date (for the unexposed).

Numbers are rounded to the nearest five as per NHS Data Access Environment (DAE) safe output guidelines.

GDPPR: General Practice Extraction Service (GPES) Data for Pandemic Planning and Research.

# Figure S2: Hazard rates of type 2 diabetes over time by body mass index, age, and exposure status, with time-varying effects of exposure and stratified by sex


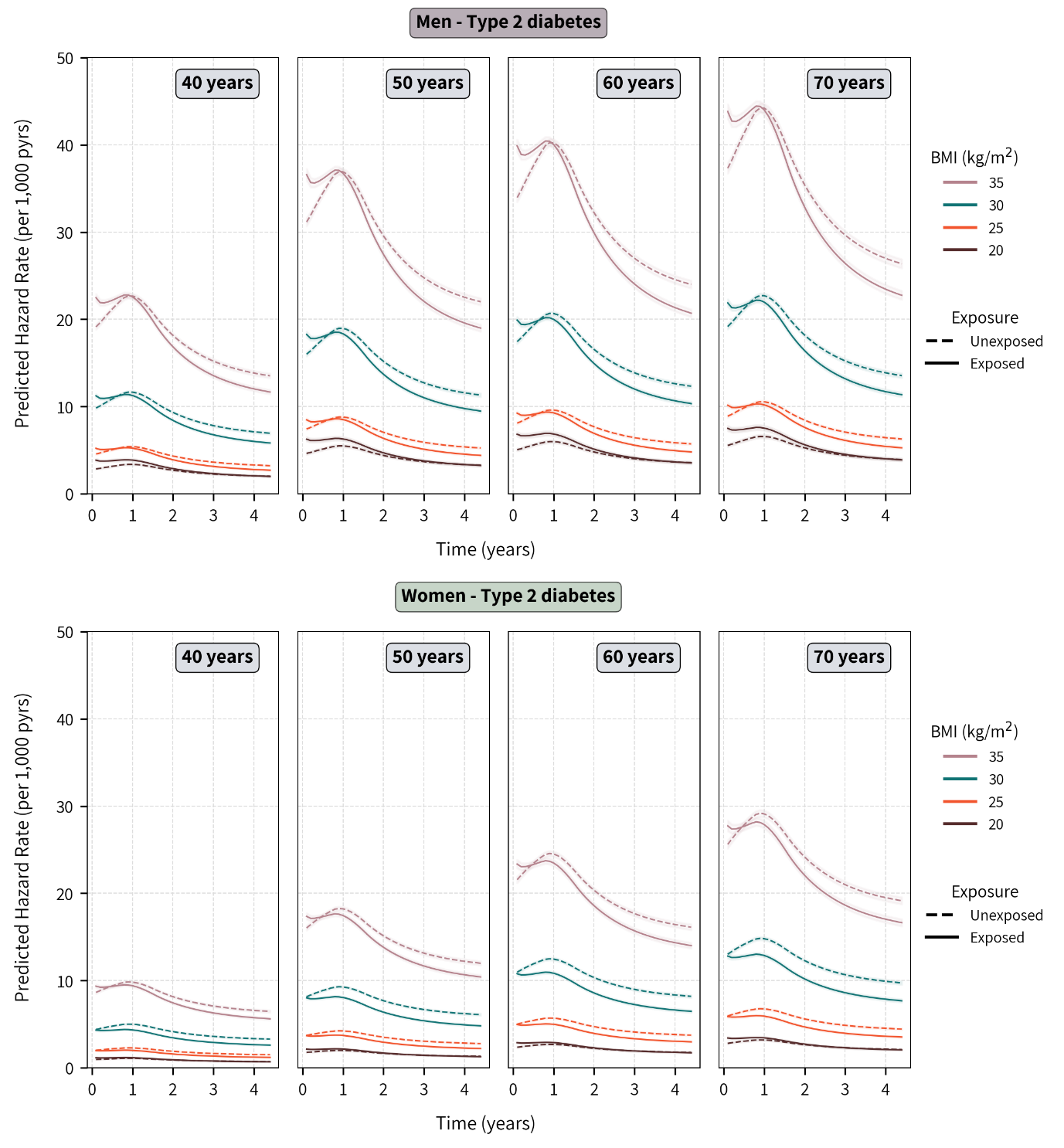


Sex-stratified predicted hazard rates obtained from flexible parametric survival models including natural cubic splines (4 degrees of freedom) of age and BMI, an interaction between BMI and exposure status, and a time-varying effect of the exposure. Time represents follow-up time from the index date (date of COVID-19 diagnosis for exposed individuals and the matched index date for unexposed individuals).

Solid lines represent exposed individuals, and dashed lines represent unexposed individuals. Shaded areas represent 95% confidence intervals. BMI: Body mass index; Pyrs: person-years.

# Figure S3: Hazard rates of type 2 diabetes over time by deprivation, age, and exposure status, stratified by sex


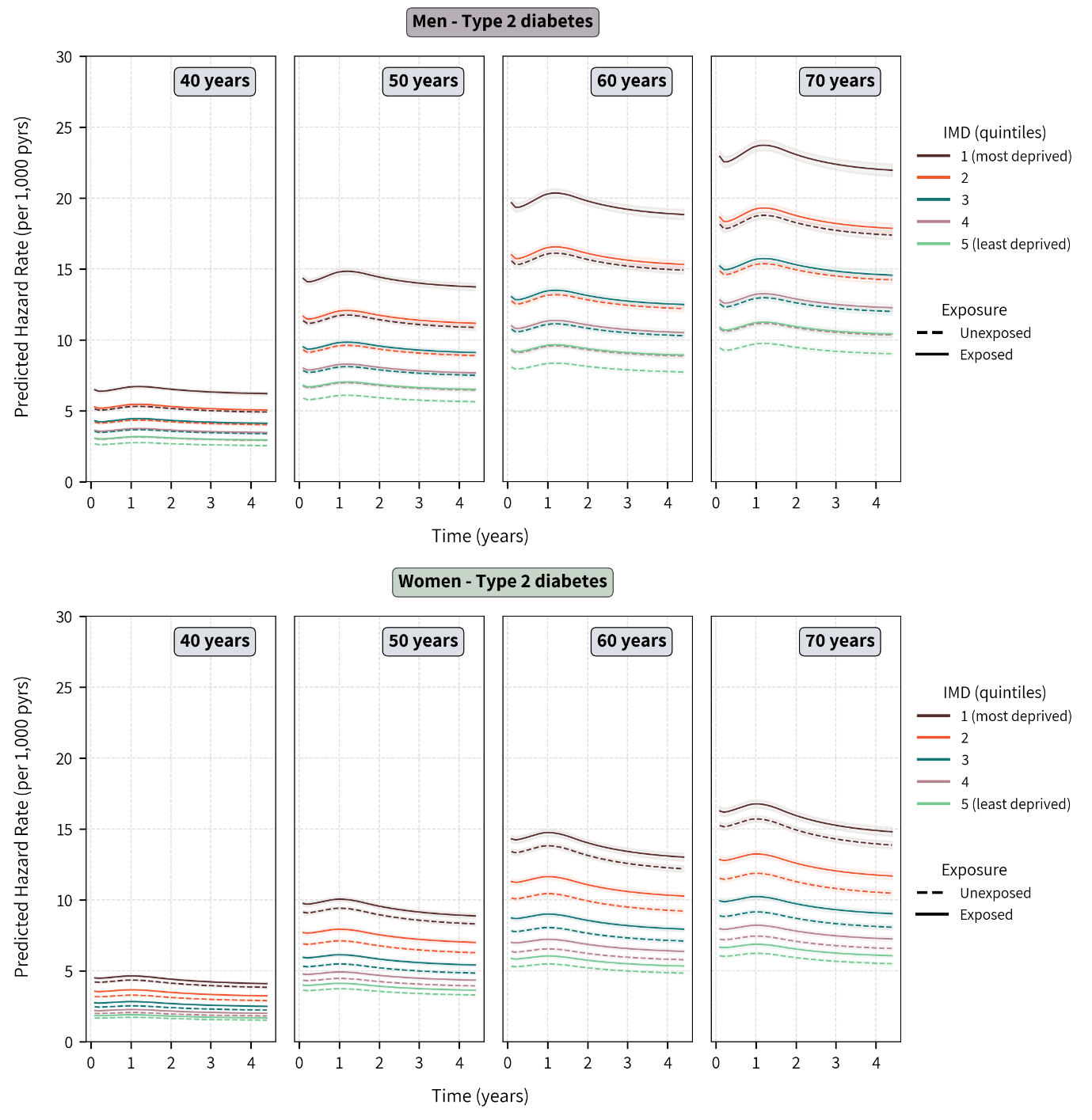


Sex-stratified predicted hazard rates obtained from flexible parametric survival models including natural cubic splines (4 degrees of freedom) of age and deprivation and an interaction between deprivation and exposure status. Time represents follow-up time from the index date (date of COVID-19 diagnosis for exposed individuals and the matched index date for unexposed individuals).

Solid lines represent exposed individuals, and dashed lines represent unexposed individuals. Shaded areas represent 95% confidence intervals. Pyrs: person-years.

# Figure S4: Hazard rates of type 2 diabetes over time by deprivation, age, and exposure status, with time-varying effects of exposure and stratified by sex


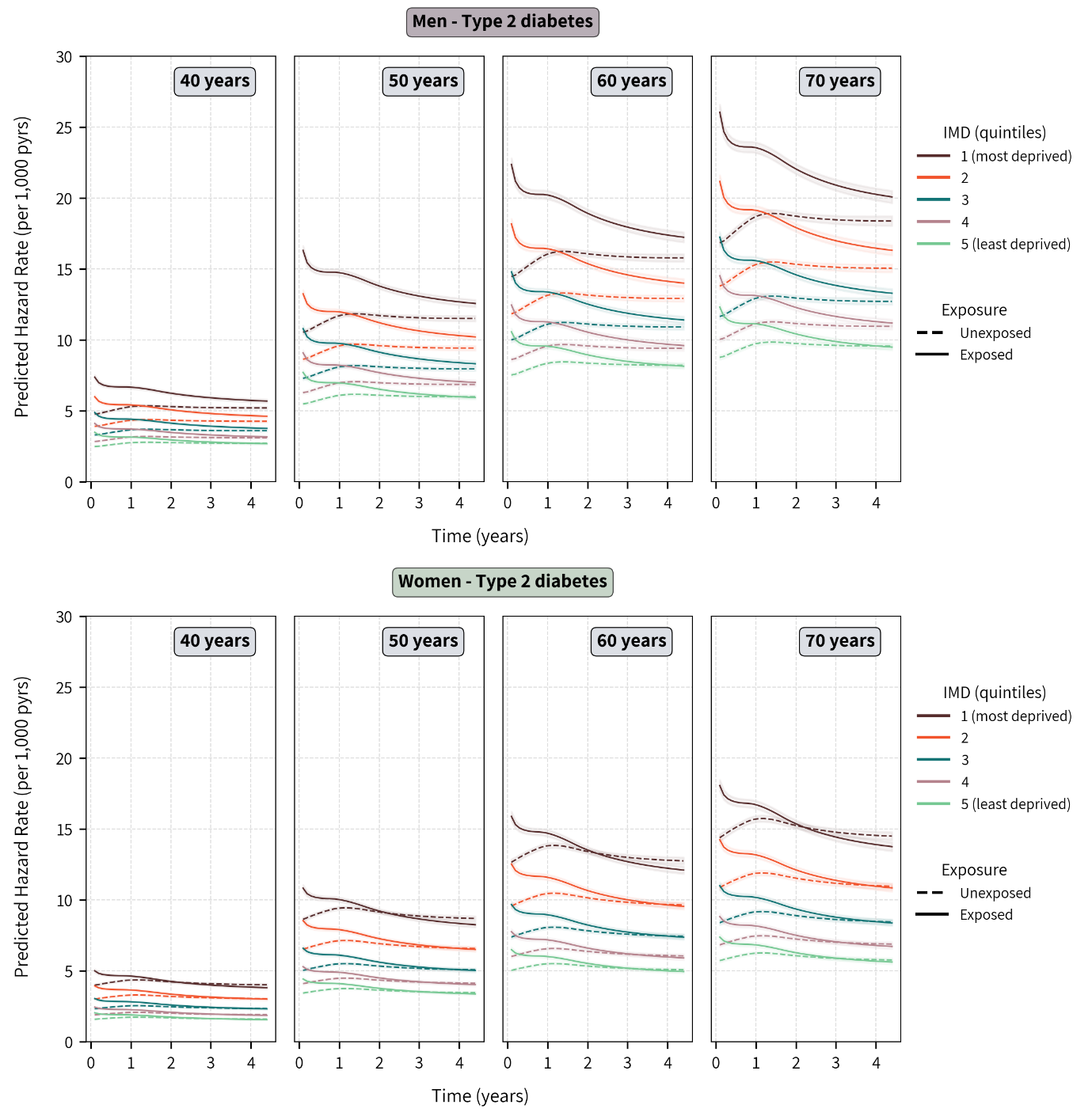


Sex-stratified predicted hazard rates obtained from flexible parametric survival models including natural cubic splines (4 degrees of freedom) of age and deprivation, an interaction between deprivation and exposure status, and a time-varying effect of the exposure. Time represents follow-up time from the index date (date of COVID-19 diagnosis for exposed individuals and the matched index date for unexposed individuals).

Solid lines represent exposed individuals, and dashed lines represent unexposed individuals. Shaded areas represent 95% confidence intervals. Pyrs: person-years.

# Figure S5: Hazard rates of type 2 diabetes over time by ethnicity, age, and exposure status, and stratified by sex


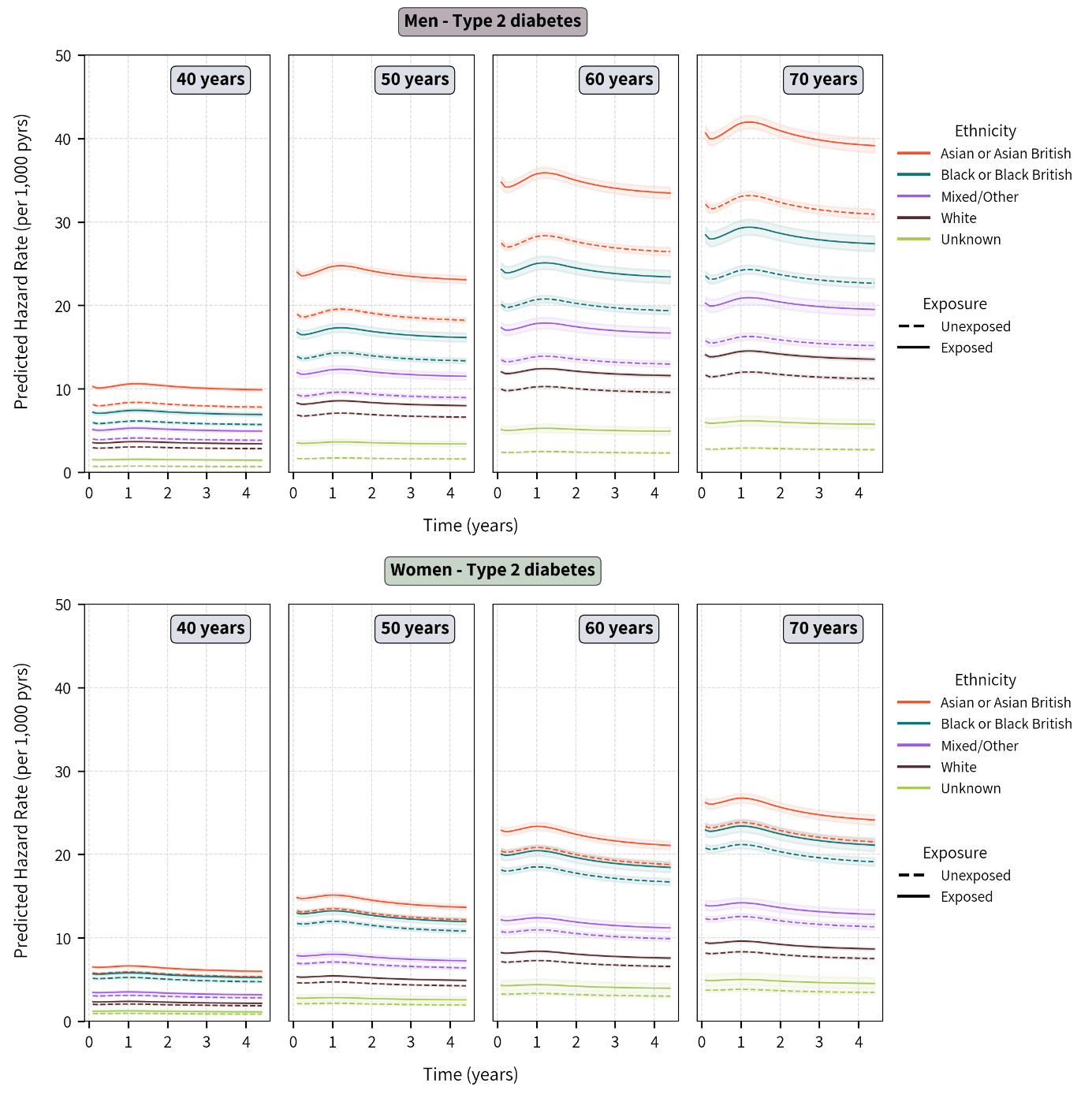


Sex-stratified predicted hazard rates obtained from flexible parametric survival models including natural cubic splines (4 degrees of freedom) of age and ethnicity and an interaction between ethnicity and exposure status. Time represents follow-up time from the index date (date of COVID-19 diagnosis for exposed individuals and the matched index date for unexposed individuals).

Solid lines represent exposed individuals, and dashed lines represent unexposed individuals. Shaded areas represent 95% confidence intervals. Pyrs: person-years.

# Figure S6: Hazard rates of type 2 diabetes over time by ethnicity, age, and exposure status, with time-varying effects of exposure and stratified by sex


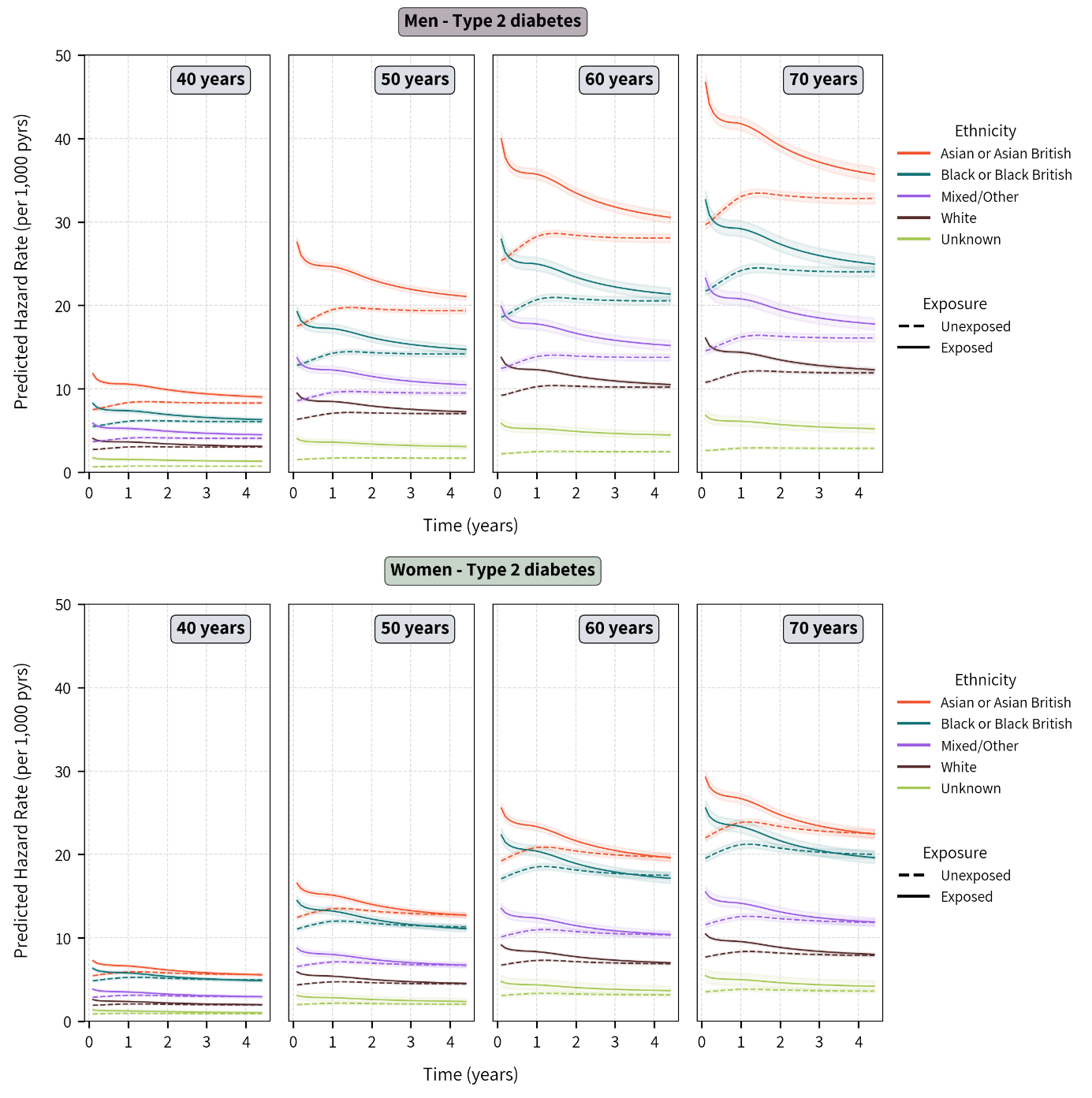


Sex-stratified predicted hazard rates obtained from flexible parametric survival models including natural cubic splines (4 degrees of freedom) of age and ethnicity, an interaction between ethnicity and exposure status, and a time-varying effect of the exposure. Time represents follow-up time from the index date (date of COVID-19 diagnosis for exposed individuals and the matched index date for unexposed individuals).

Solid lines represent exposed individuals, and dashed lines represent unexposed individuals. Shaded areas represent 95% confidence intervals. Pyrs: person-years.

# Figure S7: Hazard rates of type 2 diabetes over time by region, age, and exposure status, and stratified by sex


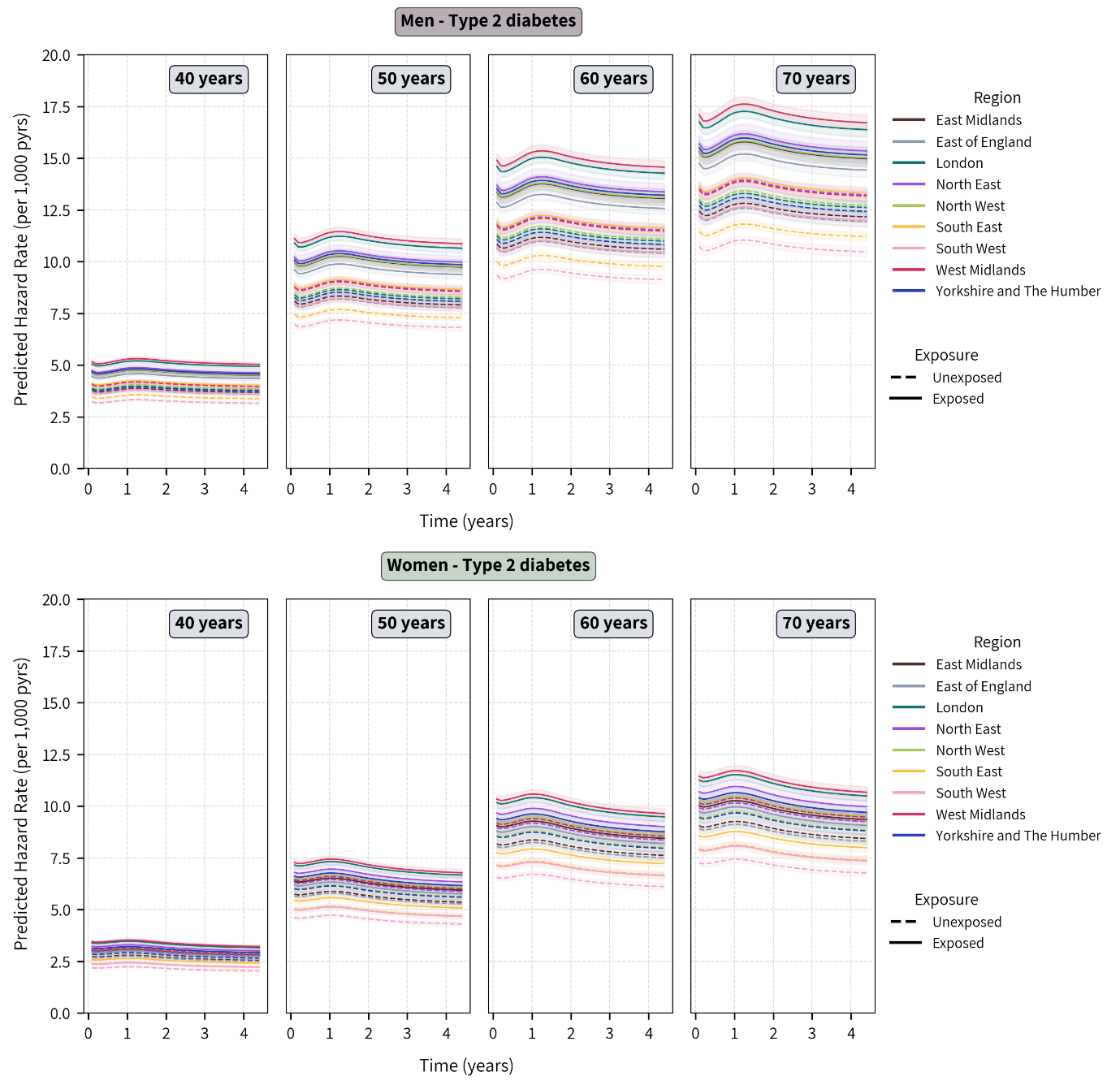


Sex-stratified predicted hazard rates obtained from flexible parametric survival models including natural cubic splines (4 degrees of freedom) of age and region and an interaction between region and exposure status. Time represents follow-up time from the index date (date of COVID-19 diagnosis for exposed individuals and the matched index date for unexposed individuals).

Solid lines represent exposed individuals, and dashed lines represent unexposed individuals. Shaded areas represent 95% confidence intervals. Pyrs: person-years.

# Figure S8: Hazard rates of type 2 diabetes over time by region, age, and exposure status, with time-varying effects of exposure and stratified by sex


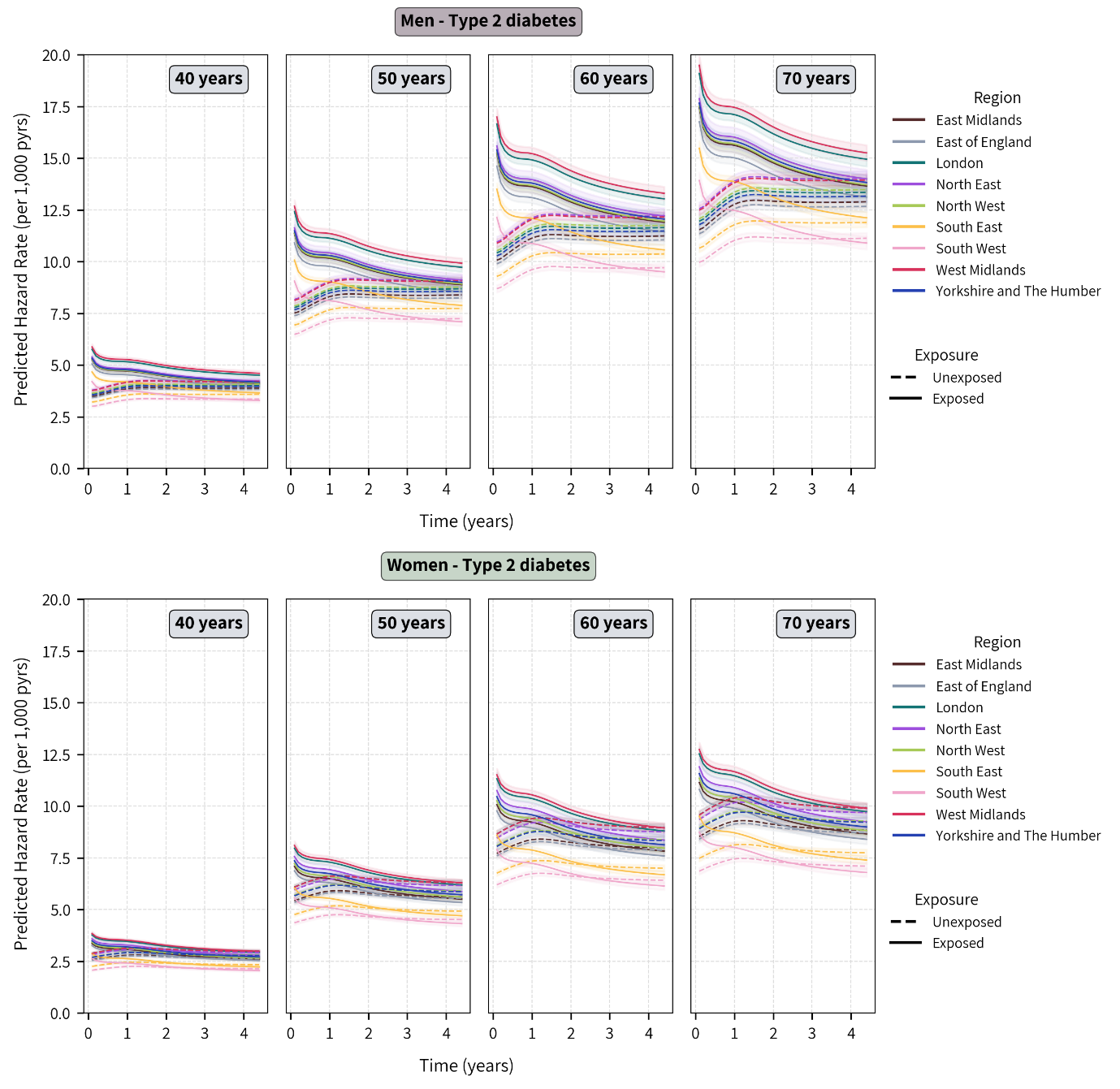


Sex-stratified predicted hazard rates obtained from flexible parametric survival models including natural cubic splines (4 degrees of freedom) of age and region, an interaction between region and exposure status, and a time-varying effect of the exposure. Time represents follow-up time from the index date (date of COVID-19 diagnosis for exposed individuals and the matched index date for unexposed individuals).

Solid lines represent exposed individuals, and dashed lines represent unexposed individuals. Shaded areas represent 95% confidence intervals. Pyrs: person-years.

# Figure S9: Hazard rates of type 1 diabetes over time by body mass index, age, and exposure status, with time-varying effects of exposure and stratified by sex


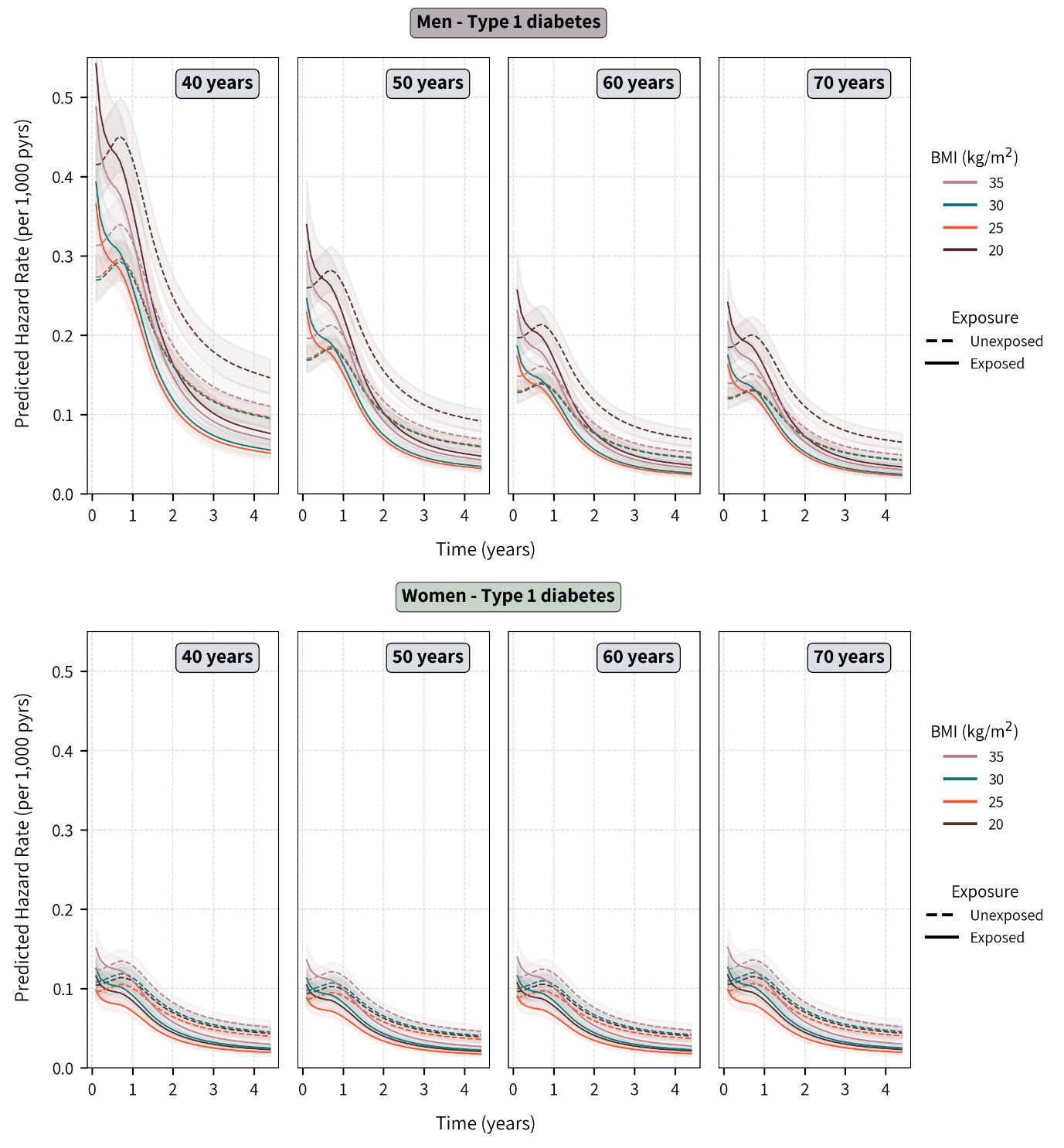


Sex-stratified predicted hazard rates obtained from flexible parametric survival models including natural cubic splines (4 degrees of freedom) of age and BMI, an interaction between BMI and exposure status, and a time-varying effect of the exposure. Time represents follow-up time from the index date (date of COVID-19 diagnosis for exposed individuals and the matched index date for unexposed individuals).

Solid lines represent exposed individuals, and dashed lines represent unexposed individuals. Shaded areas represent 95% confidence intervals. BMI: Body mass index; Pyrs: person-years.

# Figure S10: Hazard rates of type 1 diabetes over time by deprivation, age, and exposure status, stratified by sex


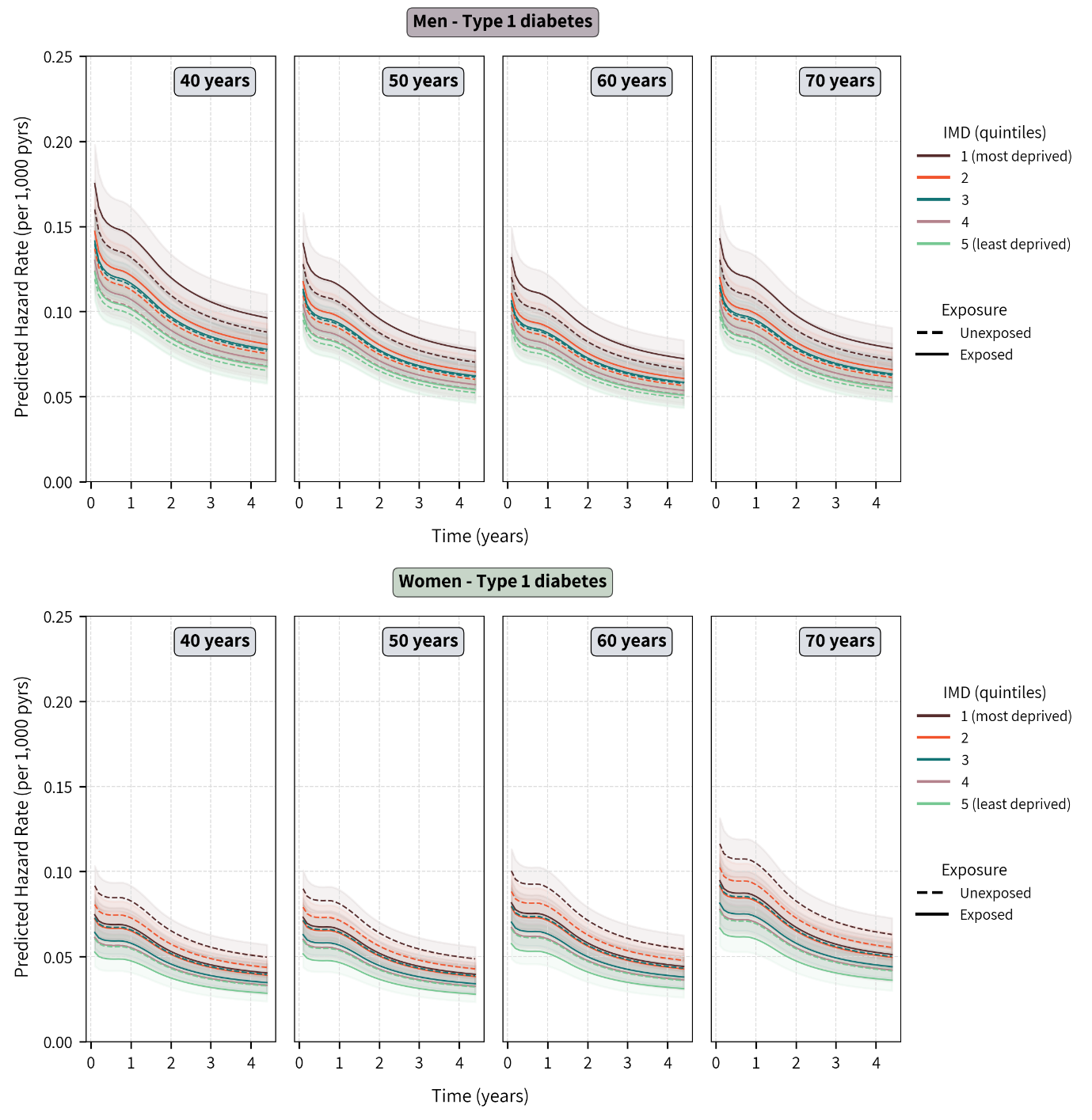


Sex-stratified predicted hazard rates obtained from flexible parametric survival models including natural cubic splines (4 degrees of freedom) of age and deprivation and an interaction between deprivation and exposure status. Time represents follow-up time from the index date (date of COVID-19 diagnosis for exposed individuals and the matched index date for unexposed individuals).

Solid lines represent exposed individuals, and dashed lines represent unexposed individuals. Shaded areas represent 95% confidence intervals. Pyrs: person-years.

# Figure S11: Hazard rates of type 1 diabetes over time by deprivation, age, and exposure status, with time-varying effects of exposure and stratified by sex


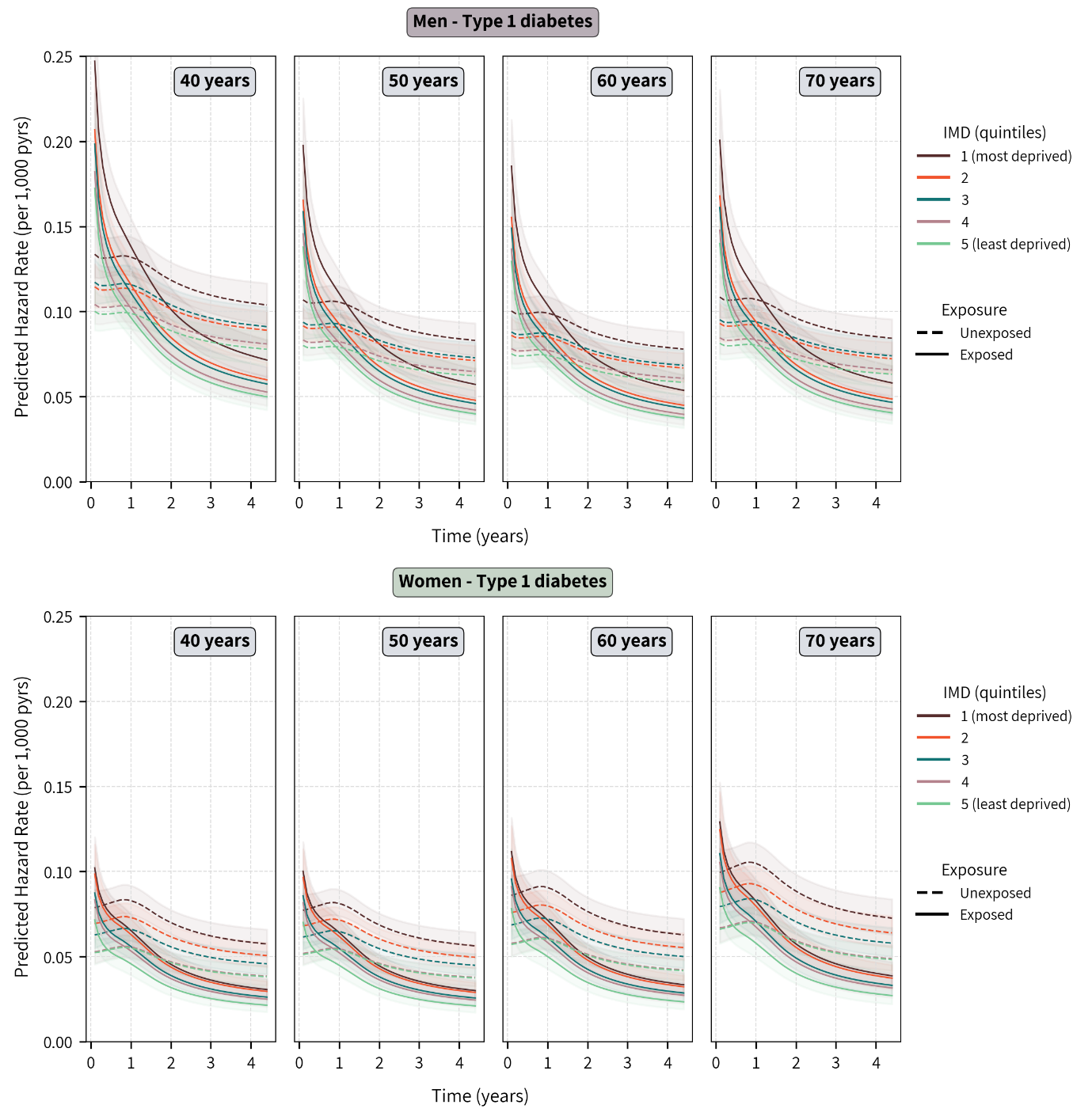


Sex-stratified predicted hazard rates obtained from flexible parametric survival models including natural cubic splines (4 degrees of freedom) of age and deprivation, an interaction between deprivation and exposure status, and a time-varying effect of the exposure. Time represents follow-up time from the index date (date of COVID-19 diagnosis for exposed individuals and the matched index date for unexposed individuals).

Solid lines represent exposed individuals, and dashed lines represent unexposed individuals. Shaded areas represent 95% confidence intervals. Pyrs: person-years.

# Figure S12: Hazard rates of type 1 diabetes over time by ethnicity, age, and exposure status, and stratified by sex


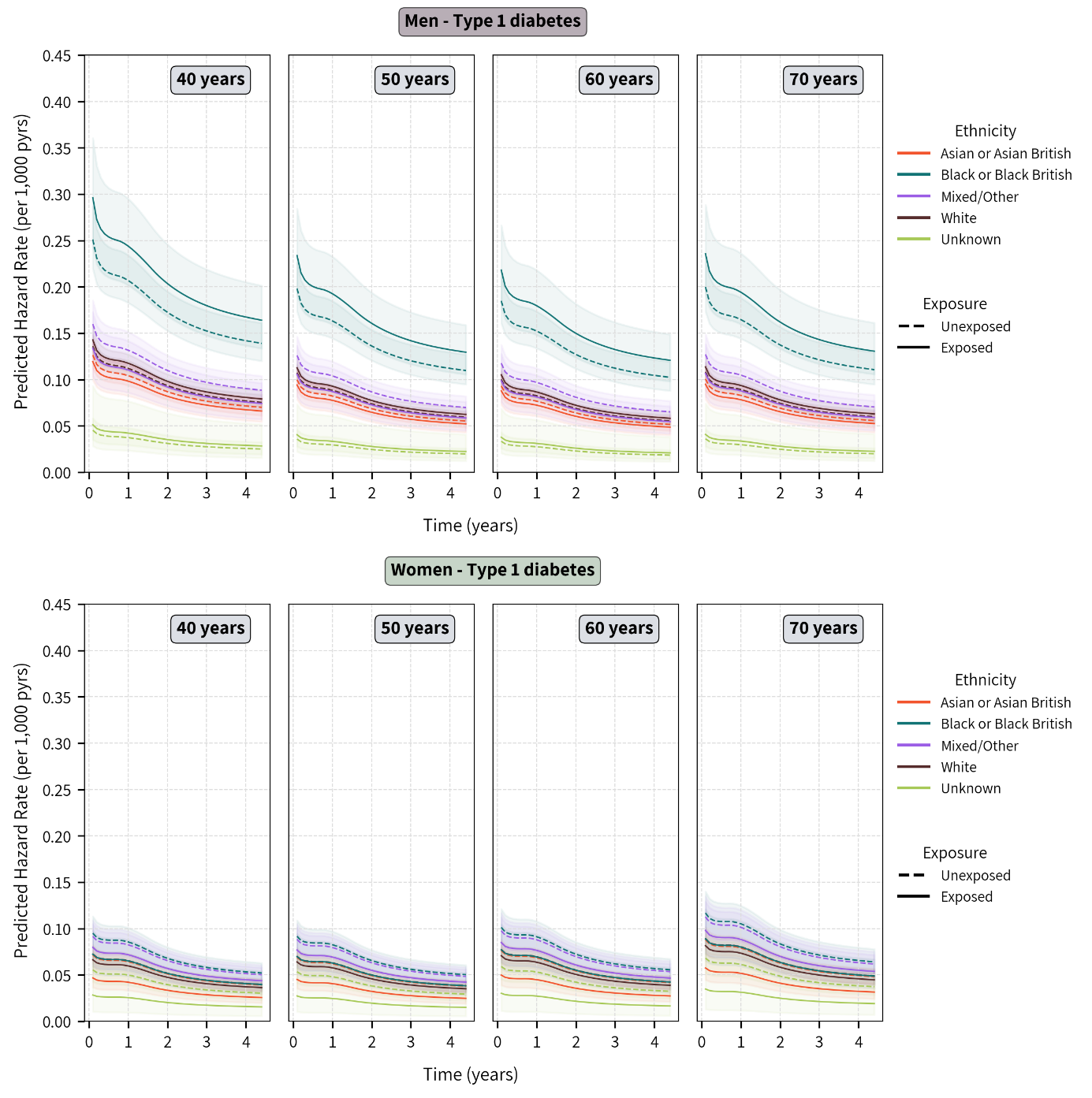


Sex-stratified predicted hazard rates obtained from flexible parametric survival models including natural cubic splines (4 degrees of freedom) of age and ethnicity and an interaction between ethnicity and exposure status. Time represents follow-up time from the index date (date of COVID-19 diagnosis for exposed individuals and the matched index date for unexposed individuals).

Solid lines represent exposed individuals, and dashed lines represent unexposed individuals. Shaded areas represent 95% confidence intervals. Pyrs: person-years.

# Figure S13: Hazard rates of type 1 diabetes over time by ethnicity, age, and exposure status, with time-varying effects of exposure and stratified by sex


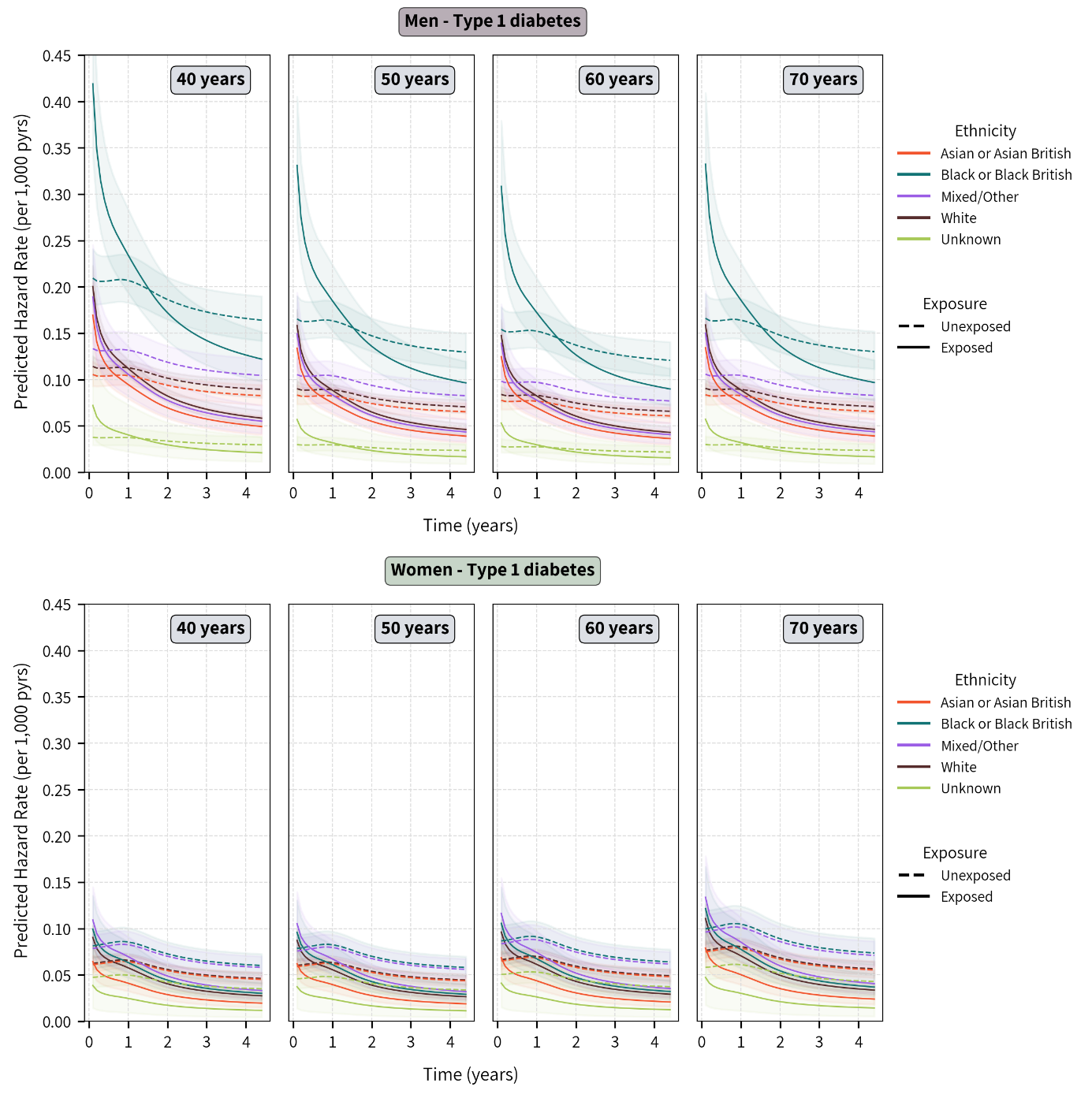


Sex-stratified predicted hazard rates obtained from flexible parametric survival models including natural cubic splines (4 degrees of freedom) of age and ethnicity, an interaction between ethnicity and exposure status, and a time-varying effect of the exposure. Time represents follow-up time from the index date (date of COVID-19 diagnosis for exposed individuals and the matched index date for unexposed individuals).

Solid lines represent exposed individuals, and dashed lines represent unexposed individuals. Shaded areas represent 95% confidence intervals. Pyrs: person-years.

# Figure S14: Hazard rates of type 1 diabetes over time by region, age, and exposure status, and stratified by sex


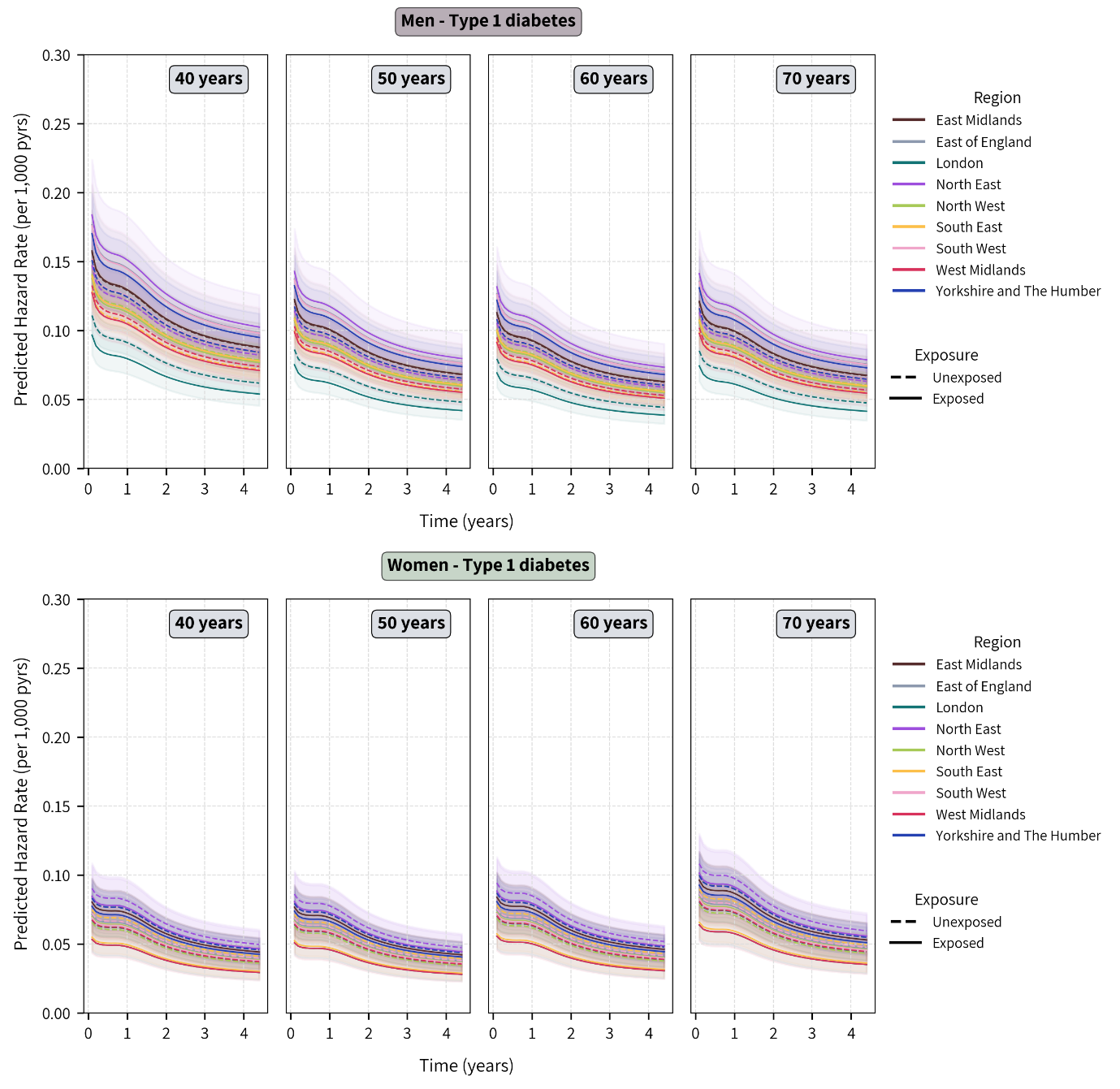


Sex-stratified predicted hazard rates obtained from flexible parametric survival models including natural cubic splines (4 degrees of freedom) of age and region and an interaction between region and exposure status. Time represents follow-up time from the index date (date of COVID-19 diagnosis for exposed individuals and the matched index date for unexposed individuals).

Solid lines represent exposed individuals, and dashed lines represent unexposed individuals. Shaded areas represent 95% confidence intervals. Pyrs: person-years.

# Figure S15: Hazard rates of type 1 diabetes over time by region, age, and exposure status, with time-varying effects of exposure and stratified by sex


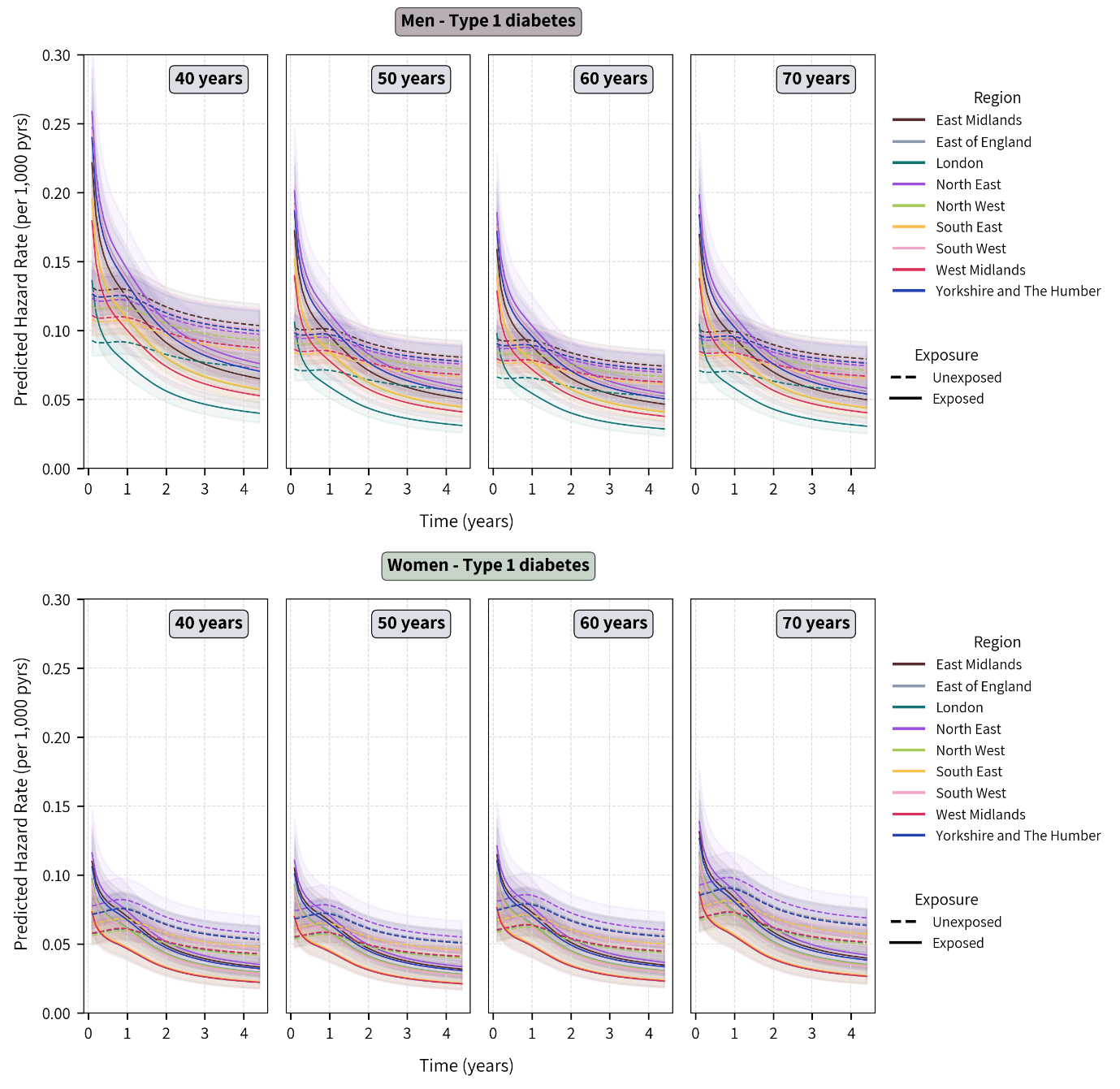


Sex-stratified predicted hazard rates obtained from flexible parametric survival models including natural cubic splines (4 degrees of freedom) of age and region, an interaction between region and exposure status, and a time-varying effect of the exposure. Time represents follow-up time from the index date (date of COVID-19 diagnosis for exposed individuals and the matched index date for unexposed individuals).

Solid lines represent exposed individuals, and dashed lines represent unexposed individuals. Shaded areas represent 95% confidence intervals. Pyrs: person-years.

# Figure S16: Study flow diagram for the cohort with outcomes defined by BHF DDSC diabetes phenotyping algorithm


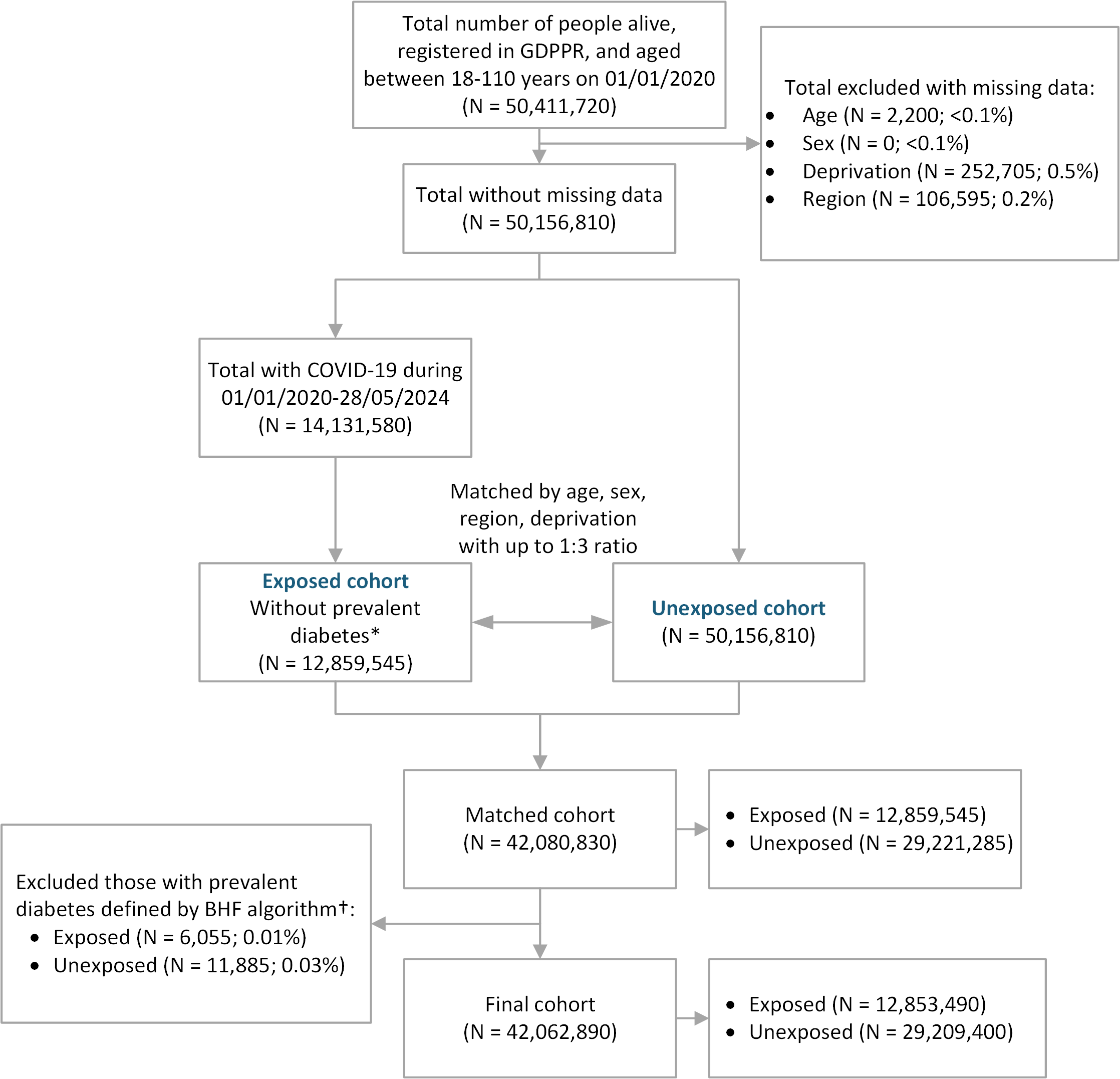


*Prevalent diabetes defined as having any record of diabetes in primary or secondary care before or at the earliest date of COVID-19 (for the exposed) or the matched index date (for the unexposed).

†From the matched cohort, individuals were further removed if they a prevalent diabetes defined by the BHF DDSC diabetes phenotype algorithm before or at the date of first COVID-19 (for the exposed) or at the index date (for the unexposed).

**Note:** Numbers are rounded to the nearest five as per NHS Data Access Environment (DAE) safe output guidelines.

GDPPR: General Practice Extraction Service (GPES) Data for Pandemic Planning and Research; BHF: British Heart Foundation; DDSC: Diabetes Data Science Catalyst.

# Figure S17: Hazard rates of type 2 diabetes (defined by BHF DDSC diabetes phenotyping algorithm) over time by body mass index, age, and exposure status, stratified by sex


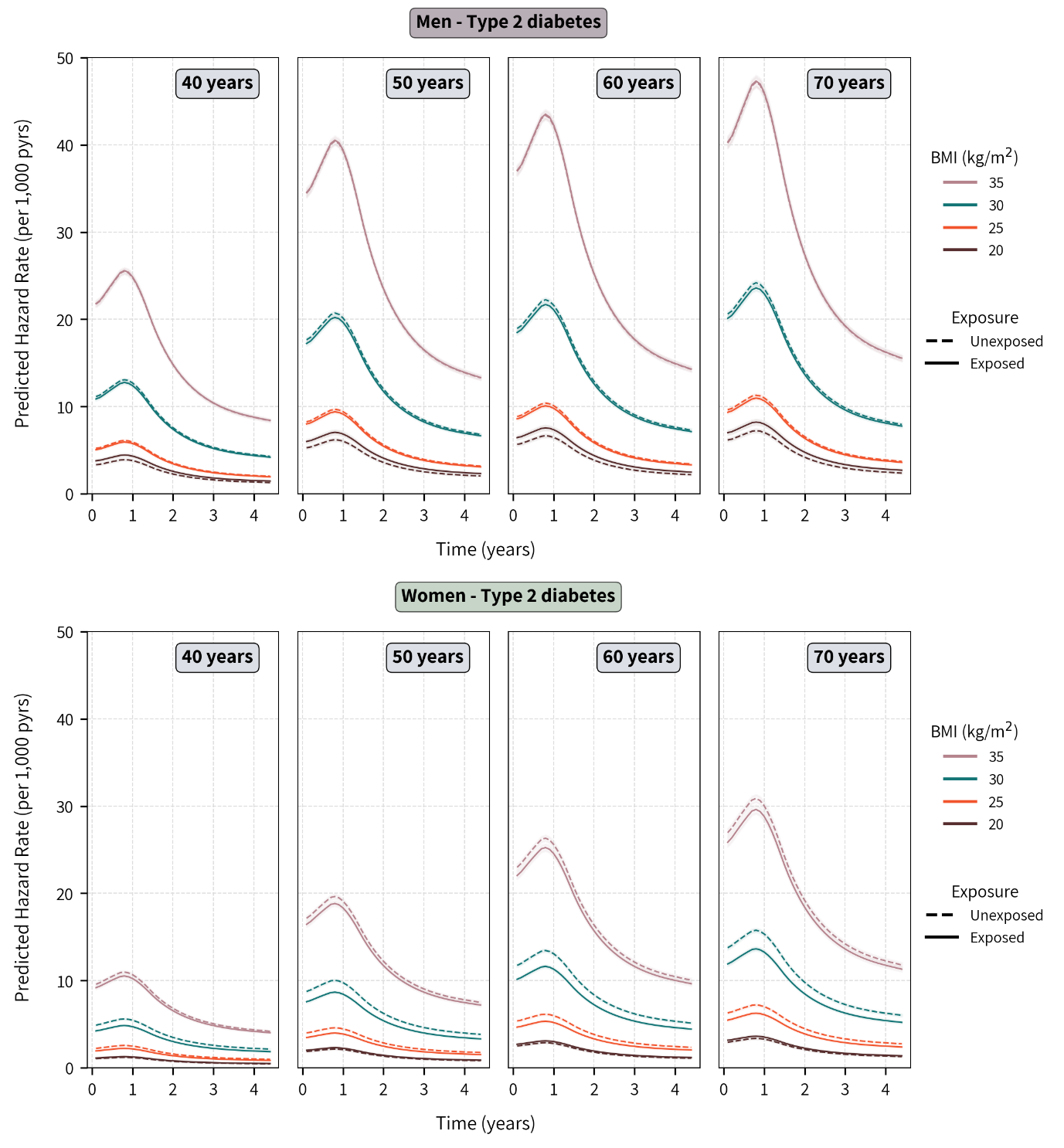


Sex-stratified predicted hazard rates obtained from flexible parametric survival models including natural cubic splines (4 degrees of freedom) of age and BMI, an interaction between BMI and exposure status. Time represents follow-up time from the index date (date of COVID-19 diagnosis for exposed individuals and the matched index date for unexposed individuals).

Solid lines represent exposed individuals, and dashed lines represent unexposed individuals. Shaded areas represent 95% confidence intervals.

BMI: Body mass index; Pyrs: person-years; BHF: British Heart Foundation; DDSC: Diabetes Data Science Catalyst.

# Figure S18: Hazard rates of type 2 diabetes (defined by BHF DDSC diabetes phenotyping algorithm) over time by body mass index, age, and exposure status, with time-varying effects of exposure and stratified by sex


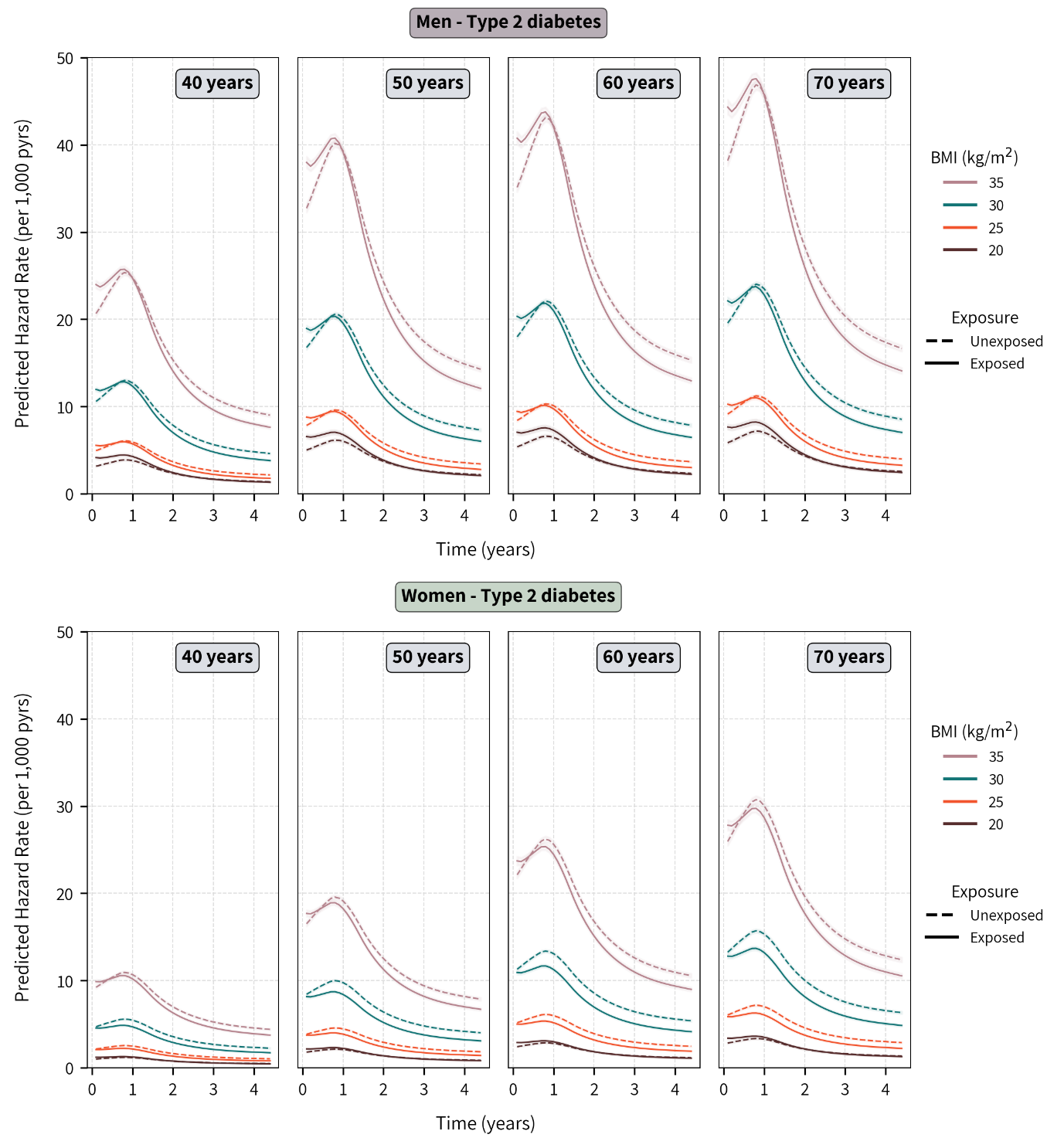


Sex-stratified predicted hazard rates obtained from flexible parametric survival models including natural cubic splines (4 degrees of freedom) of age and BMI, an interaction between BMI and exposure status, and a time-varying effect of the exposure. Time represents follow-up time from the index date (date of COVID-19 diagnosis for exposed individuals and the matched index date for unexposed individuals).

Solid lines represent exposed individuals, and dashed lines represent unexposed individuals. Shaded areas represent 95% confidence intervals.

BMI: Body mass index; Pyrs: person-years; BHF: British Heart Foundation; DDSC: Diabetes Data Science Catalyst.

# Figure S19: Hazard rates of type 2 diabetes (defined by BHF DDSC diabetes phenotyping algorithm) over time by deprivation, age, and exposure status, stratified by sex


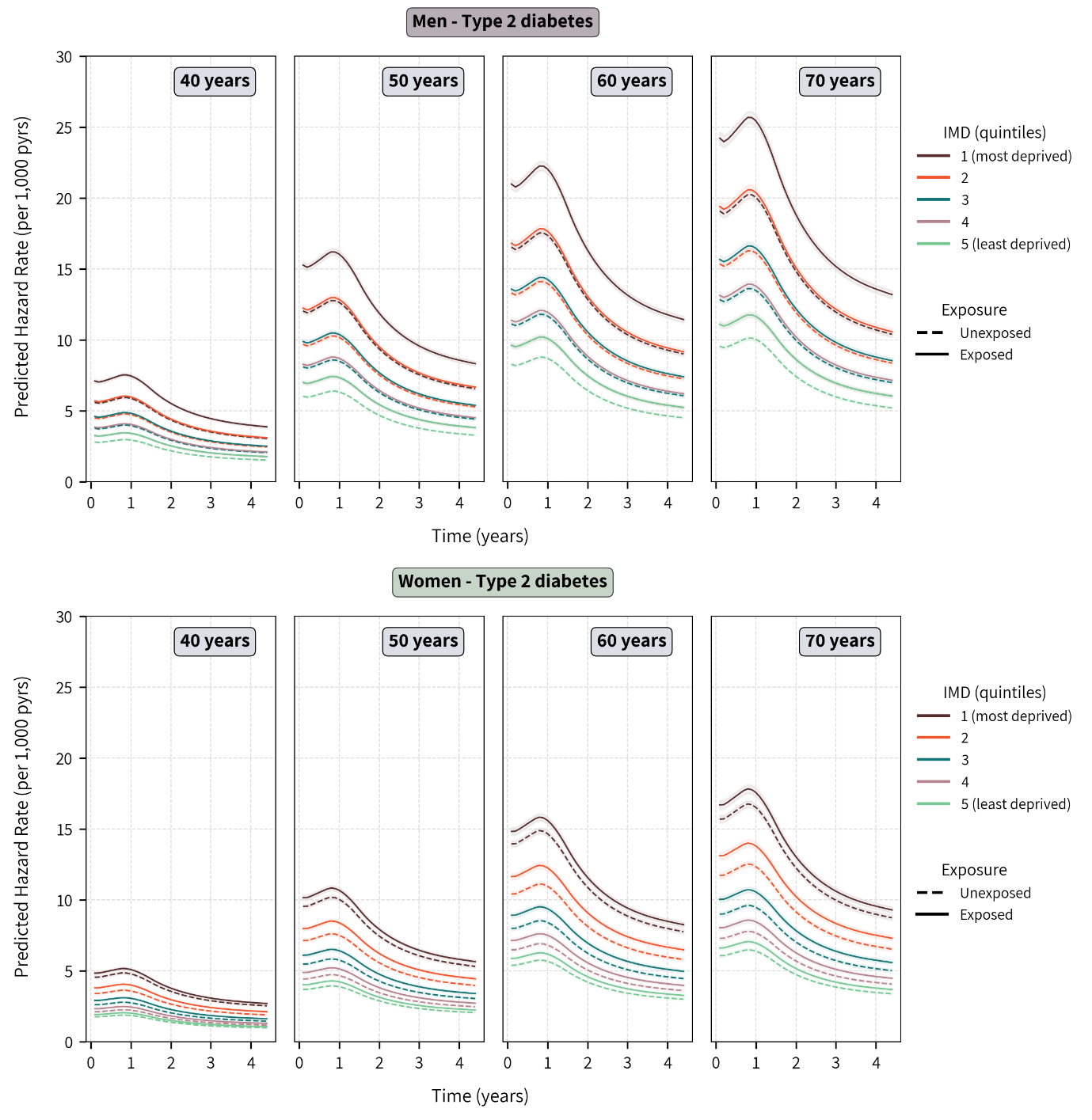


Sex-stratified predicted hazard rates obtained from flexible parametric survival models including natural cubic splines (4 degrees of freedom) of age and deprivation and an interaction between deprivation and exposure status. Time represents follow-up time from the index date (date of COVID-19 diagnosis for exposed individuals and the matched index date for unexposed individuals).

Solid lines represent exposed individuals, and dashed lines represent unexposed individuals. Shaded areas represent 95% confidence intervals.

Pyrs: person-years; BHF: British Heart Foundation; DDSC: Diabetes Data Science Catalyst.

# Figure S20: Hazard rates of type 2 diabetes (defined by BHF DDSC diabetes phenotyping algorithm) over time by deprivation, age, and exposure status, with time-varying effects of exposure and stratified by sex


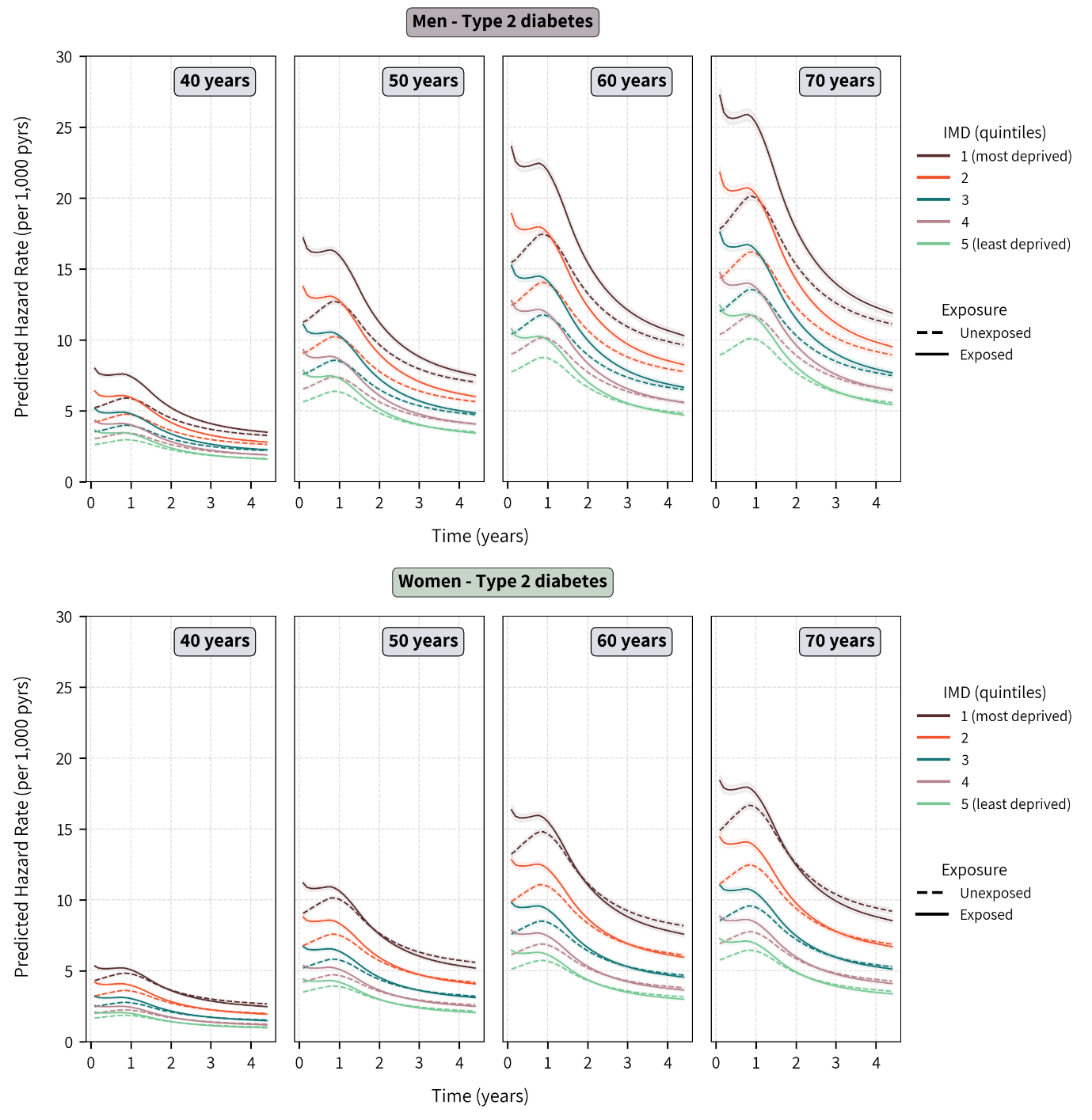


Sex-stratified predicted hazard rates obtained from flexible parametric survival models including natural cubic splines (4 degrees of freedom) of age and deprivation, an interaction between deprivation and exposure status, and a time-varying effect of the exposure. Time represents follow-up time from the index date (date of COVID-19 diagnosis for exposed individuals and the matched index date for unexposed individuals).

Solid lines represent exposed individuals, and dashed lines represent unexposed individuals. Shaded areas represent 95% confidence intervals.

Pyrs: person-years; BHF: British Heart Foundation; DDSC: Diabetes Data Science Catalyst.

# Figure S21: Hazard rates of type 2 diabetes (defined by BHF DDSC diabetes phenotyping algorithm) over time by ethnicity, age, and exposure status, and stratified by sex


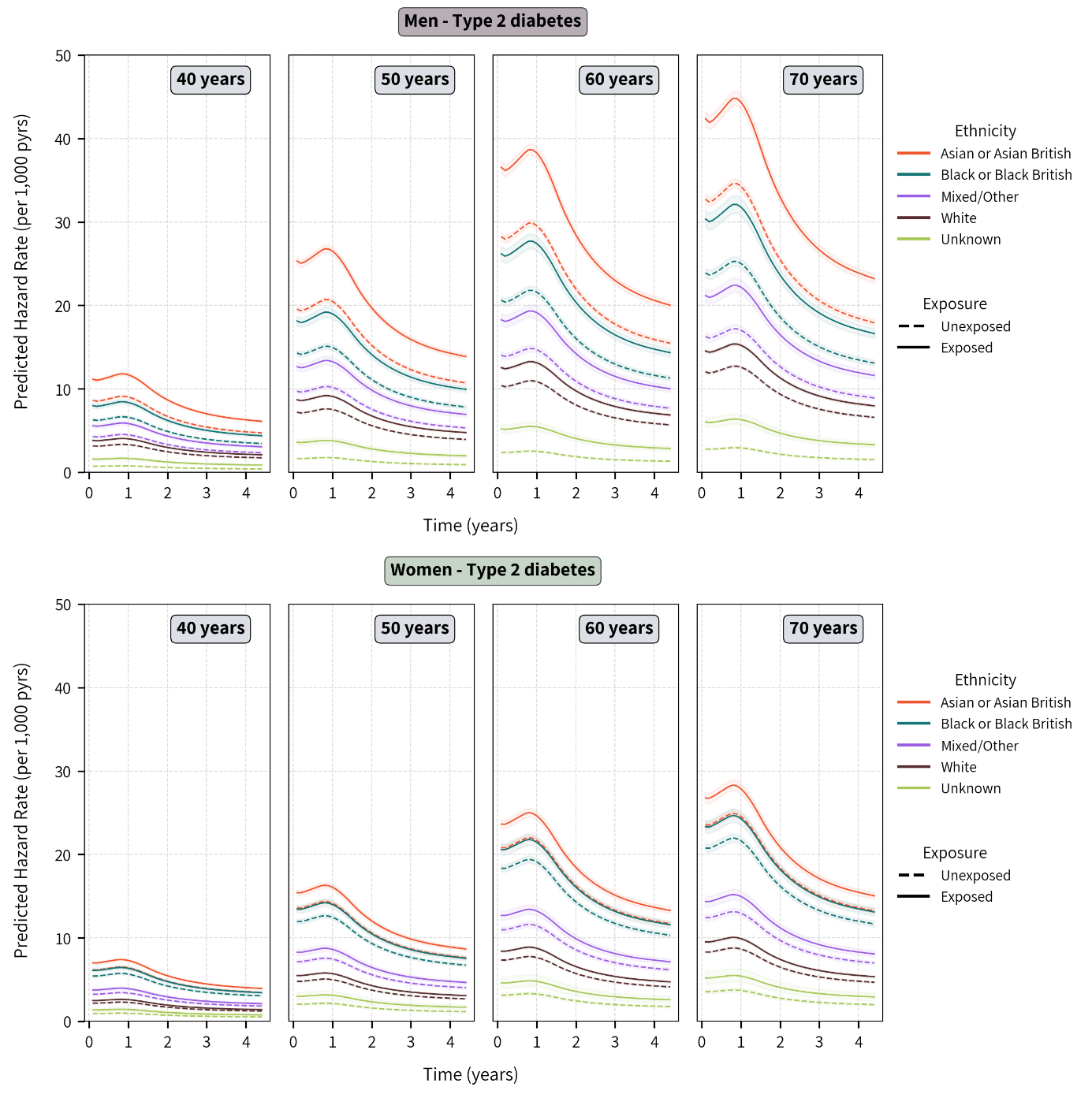


Sex-stratified predicted hazard rates obtained from flexible parametric survival models including natural cubic splines (4 degrees of freedom) of age and ethnicity and an interaction between ethnicity and exposure status. Time represents follow-up time from the index date (date of COVID-19 diagnosis for exposed individuals and the matched index date for unexposed individuals).

Solid lines represent exposed individuals, and dashed lines represent unexposed individuals. Shaded areas represent 95% confidence intervals.

Pyrs: person-years; BHF: British Heart Foundation; DDSC: Diabetes Data Science Catalyst.

# Figure S22: Hazard rates of type 2 diabetes (defined by BHF DDSC diabetes phenotyping algorithm) over time by ethnicity, age, and exposure status, with time-varying effects of exposure and stratified by sex


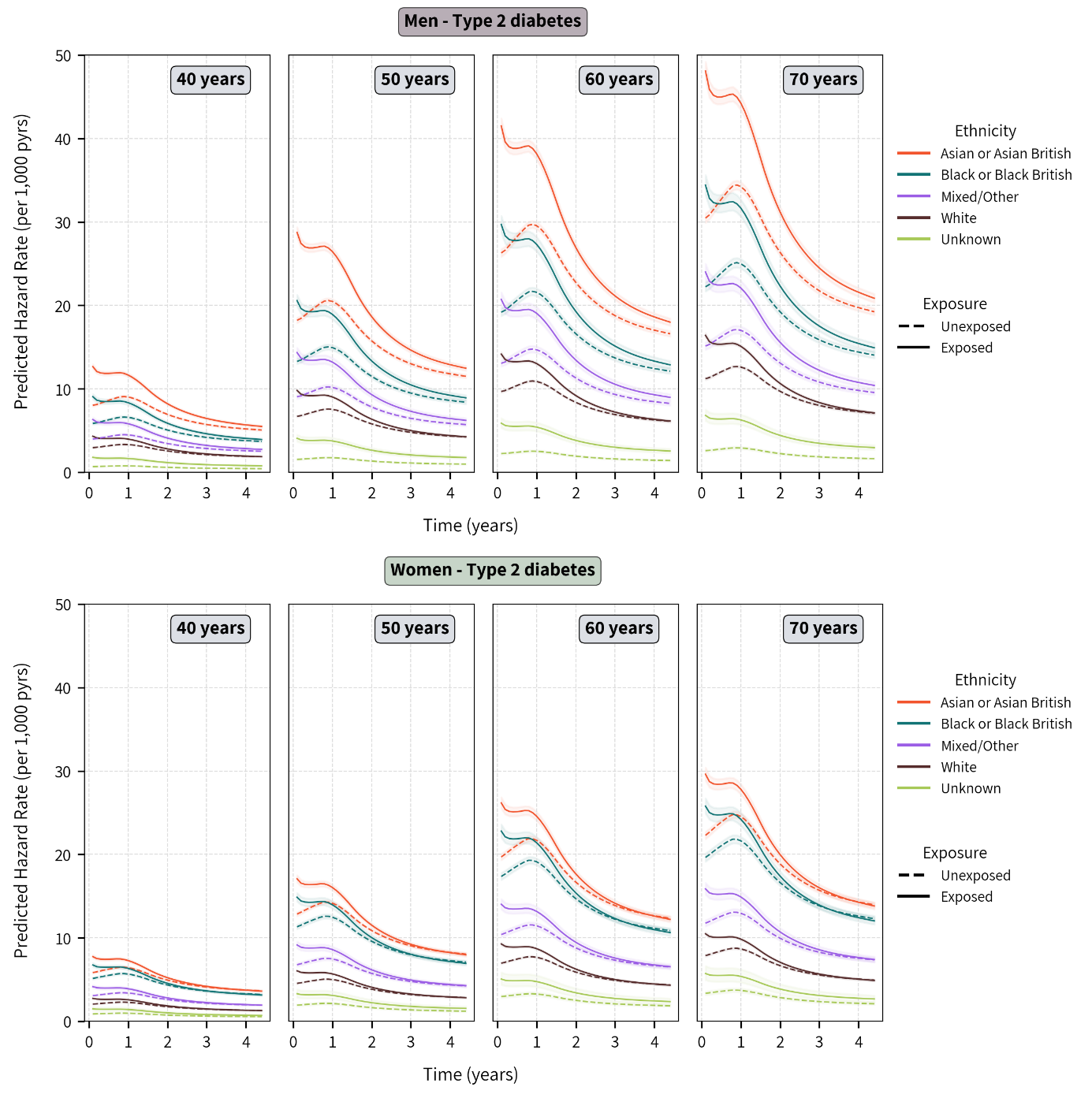


Sex-stratified predicted hazard rates obtained from flexible parametric survival models including natural cubic splines (4 degrees of freedom) of age and ethnicity, an interaction between ethnicity and exposure status, and a time-varying effect of the exposure. Time represents follow-up time from the index date (date of COVID-19 diagnosis for exposed individuals and the matched index date for unexposed individuals).

Solid lines represent exposed individuals, and dashed lines represent unexposed individuals. Shaded areas represent 95% confidence intervals.

Pyrs: person-years; BHF: British Heart Foundation; DDSC: Diabetes Data Science Catalyst.

# Figure S23: Hazard rates of type 2 diabetes (defined by BHF DDSC diabetes phenotyping algorithm) over time by region, age, and exposure status, and stratified by sex


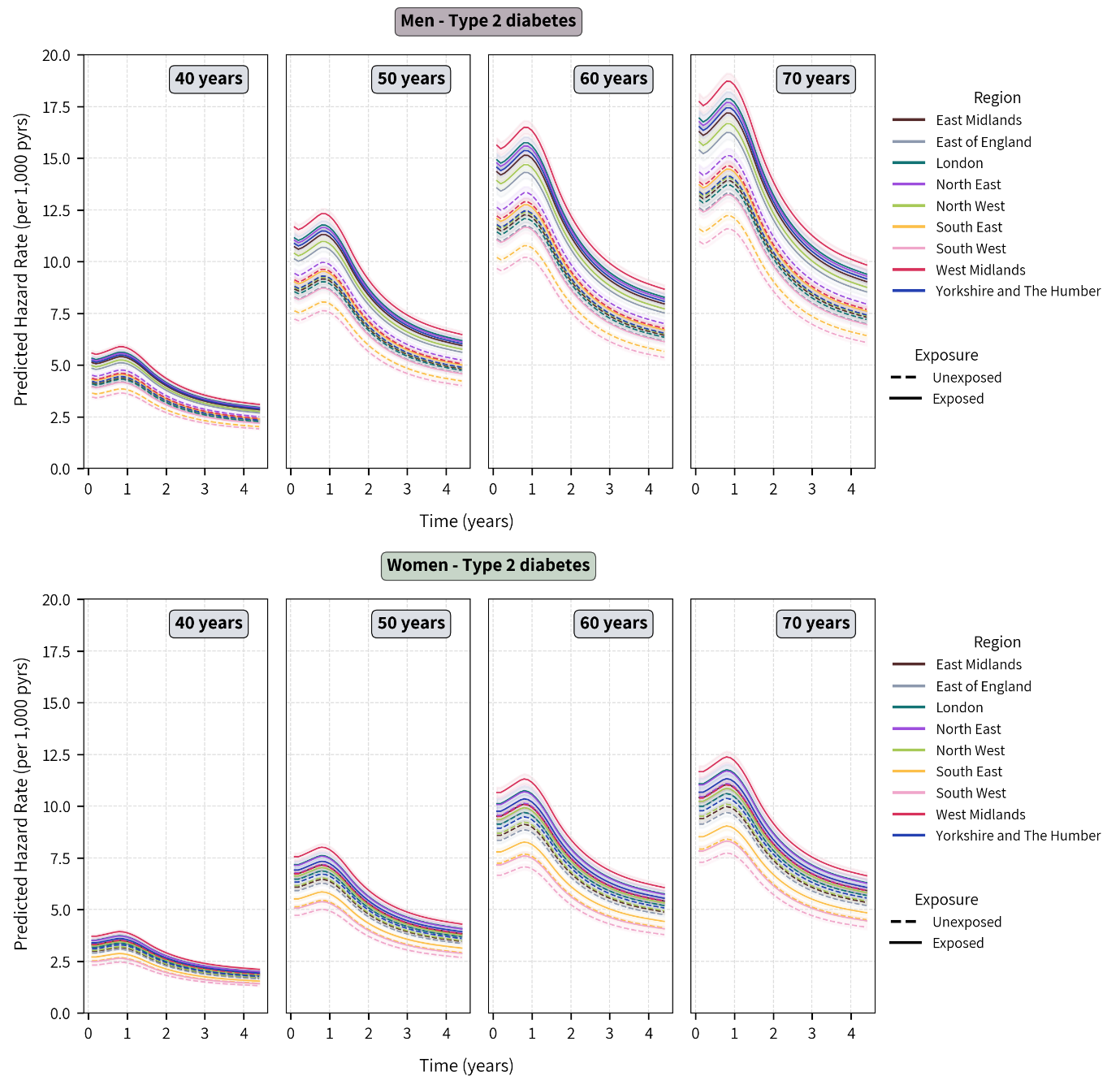


Sex-stratified predicted hazard rates obtained from flexible parametric survival models including natural cubic splines (4 degrees of freedom) of age and region and an interaction between region and exposure status. Time represents follow-up time from the index date (date of COVID-19 diagnosis for exposed individuals and the matched index date for unexposed individuals).

Solid lines represent exposed individuals, and dashed lines represent unexposed individuals. Shaded areas represent 95% confidence intervals.

Pyrs: person-years; BHF: British Heart Foundation; DDSC: Diabetes Data Science Catalyst.

# Figure S24: Hazard rates of type 2 diabetes (defined by BHF DDSC diabetes phenotyping algorithm) over time by region, age, and exposure status, with time-varying effects of exposure and stratified by sex


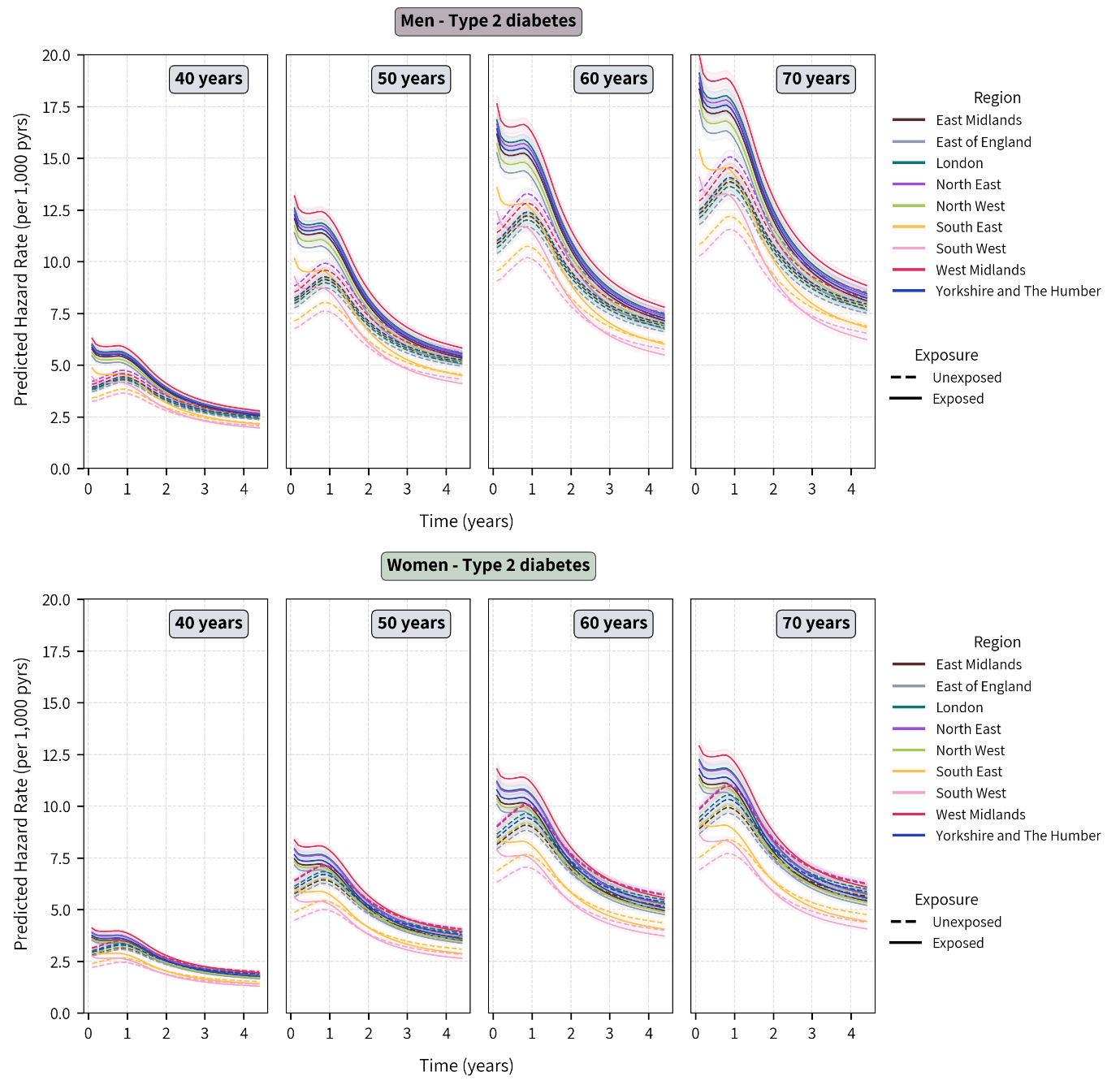


Sex-stratified predicted hazard rates obtained from flexible parametric survival models including natural cubic splines (4 degrees of freedom) of age and region, an interaction between region and exposure status, and a time-varying effect of the exposure. Time represents follow-up time from the index date (date of COVID-19 diagnosis for exposed individuals and the matched index date for unexposed individuals).

Solid lines represent exposed individuals, and dashed lines represent unexposed individuals. Shaded areas represent 95% confidence intervals.

Pyrs: person-years; BHF: British Heart Foundation; DDSC: Diabetes Data Science Catalyst.

# Figure S25: Hazard rates of type 1 diabetes (defined by BHF DDSC diabetes phenotyping algorithm) over time by body mass index, age, and exposure status, stratified by sex


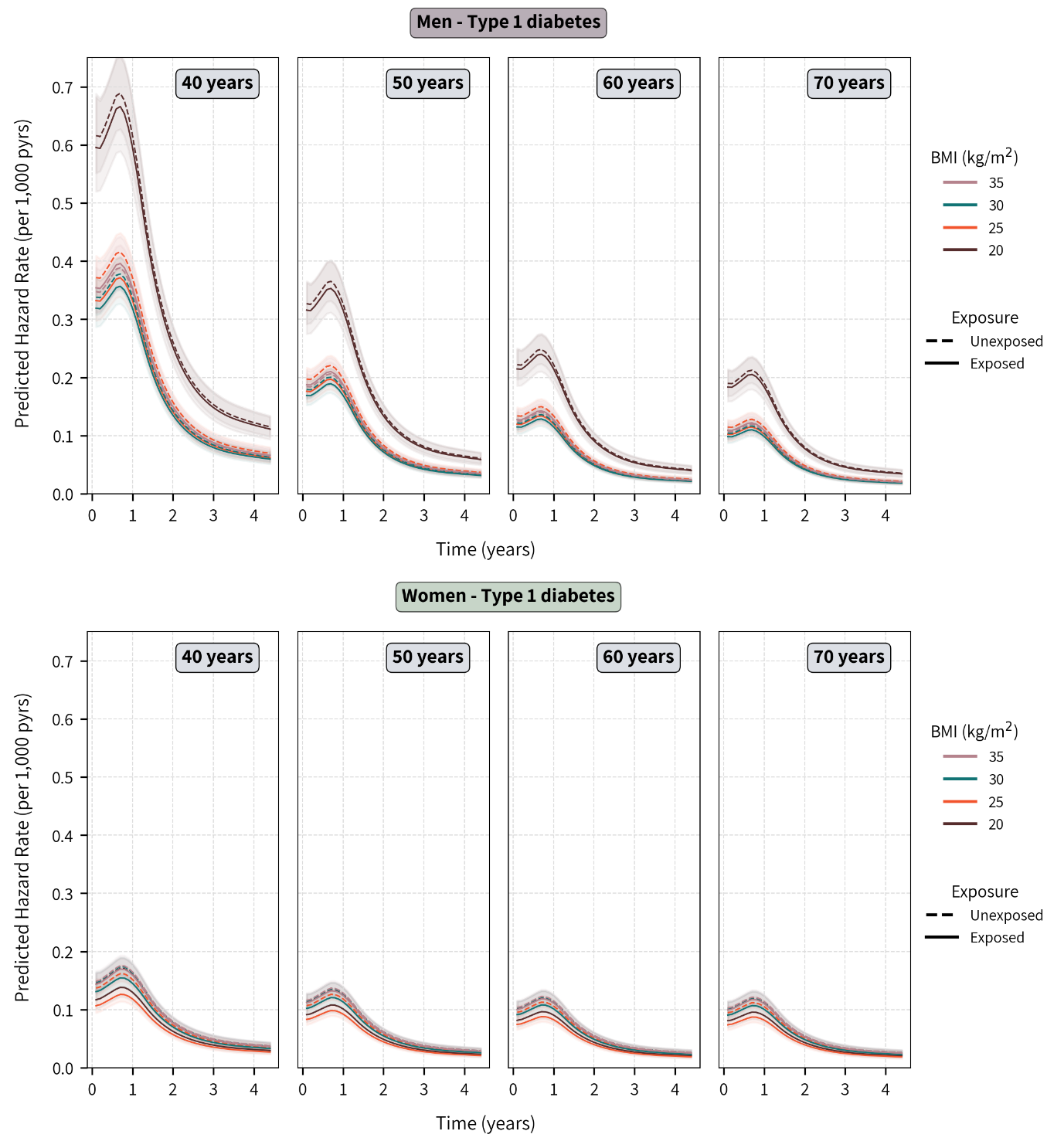


Sex-stratified predicted hazard rates obtained from flexible parametric survival models including natural cubic splines (4 degrees of freedom) of age and BMI, an interaction between BMI and exposure status. Time represents follow-up time from the index date (date of COVID-19 diagnosis for exposed individuals and the matched index date for unexposed individuals).

Solid lines represent exposed individuals, and dashed lines represent unexposed individuals. Shaded areas represent 95% confidence intervals.

BMI: Body mass index; Pyrs: person-years; BHF: British Heart Foundation; DDSC: Diabetes Data Science Catalyst.

# Figure S26: Hazard rates of type 1 diabetes (defined by BHF DDSC diabetes phenotyping algorithm) over time by body mass index, age, and exposure status, with time-varying effects of exposure and stratified by sex


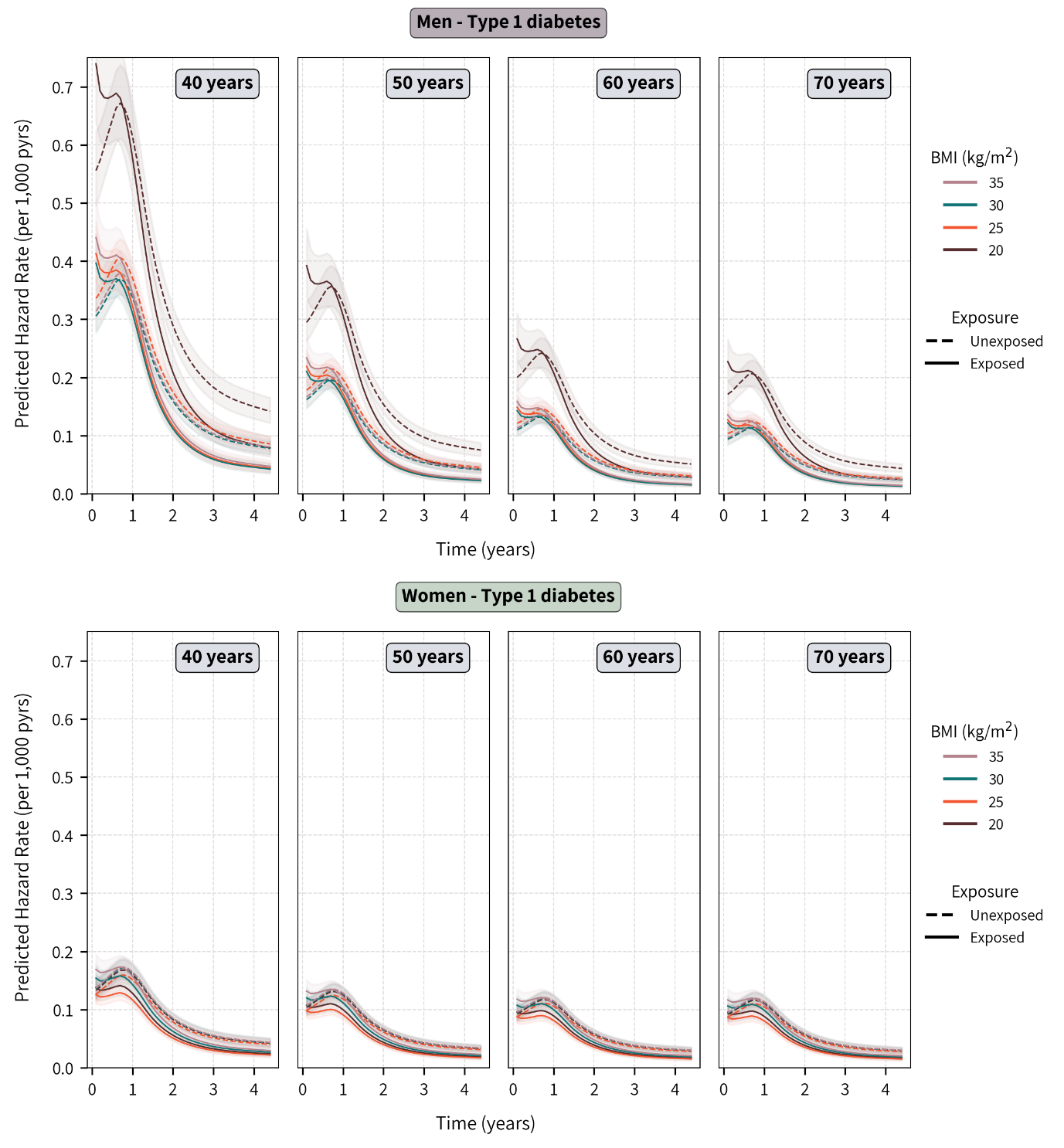


Sex-stratified predicted hazard rates obtained from flexible parametric survival models including natural cubic splines (4 degrees of freedom) of age and BMI, an interaction between BMI and exposure status, and a time-varying effect of the exposure. Time represents follow-up time from the index date (date of COVID-19 diagnosis for exposed individuals and the matched index date for unexposed individuals).

Solid lines represent exposed individuals, and dashed lines represent unexposed individuals. Shaded areas represent 95% confidence intervals.

BMI: Body mass index; Pyrs: person-years; BHF: British Heart Foundation; DDSC: Diabetes Data Science Catalyst.

# Figure S27: Hazard rates of type 1 diabetes (defined by BHF DDSC diabetes phenotyping algorithm) over time by deprivation, age, and exposure status, stratified by sex


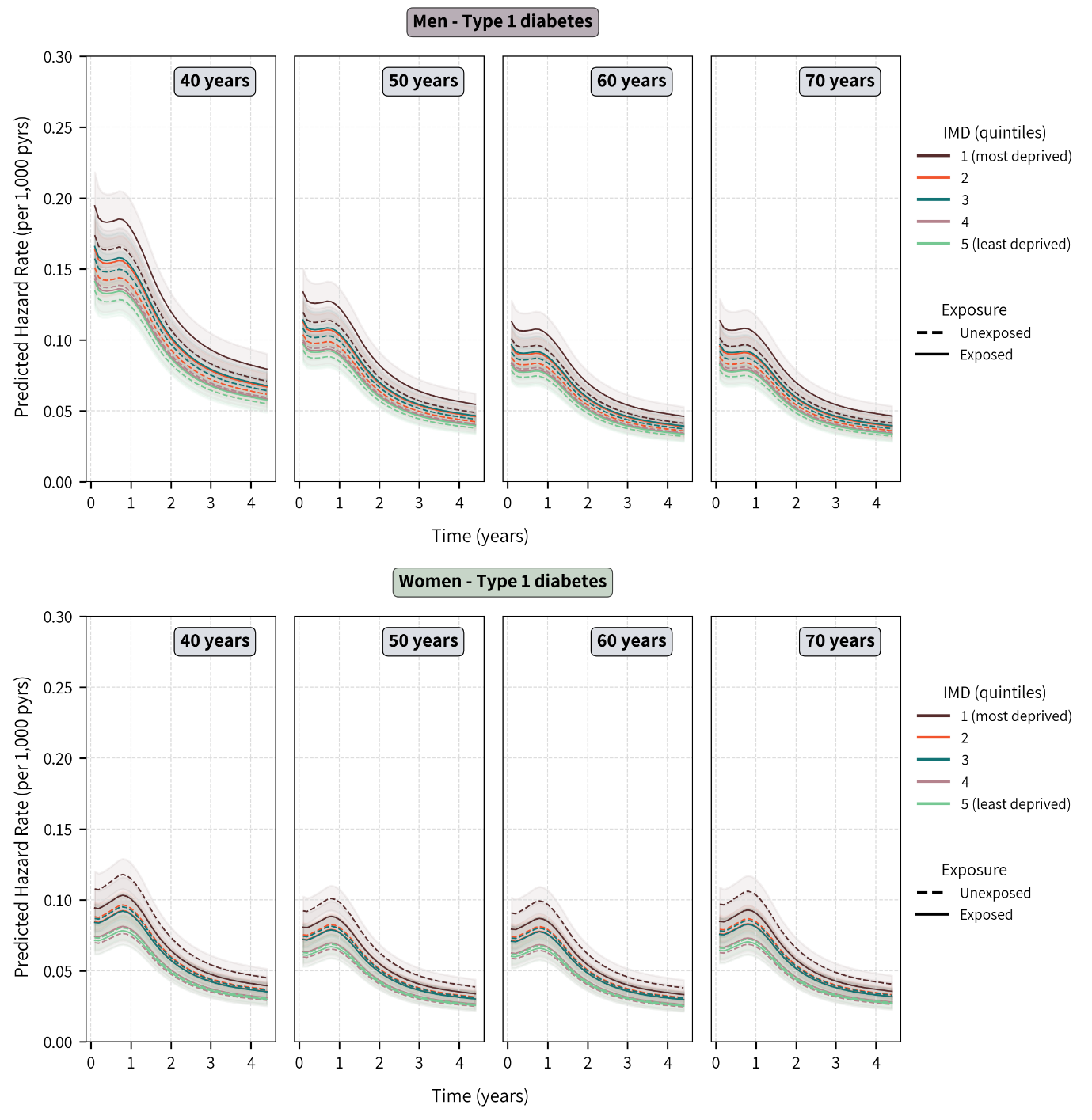


Sex-stratified predicted hazard rates obtained from flexible parametric survival models including natural cubic splines (4 degrees of freedom) of age and deprivation and an interaction between deprivation and exposure status. Time represents follow-up time from the index date (date of COVID-19 diagnosis for exposed individuals and the matched index date for unexposed individuals).

Solid lines represent exposed individuals, and dashed lines represent unexposed individuals. Shaded areas represent 95% confidence intervals.

Pyrs: person-years; BHF: British Heart Foundation; DDSC: Diabetes Data Science Catalyst.

# Figure S28: Hazard rates of type 1 diabetes (defined by BHF DDSC diabetes phenotyping algorithm) over time by deprivation, age, and exposure status, with time-varying effects of exposure and stratified by sex


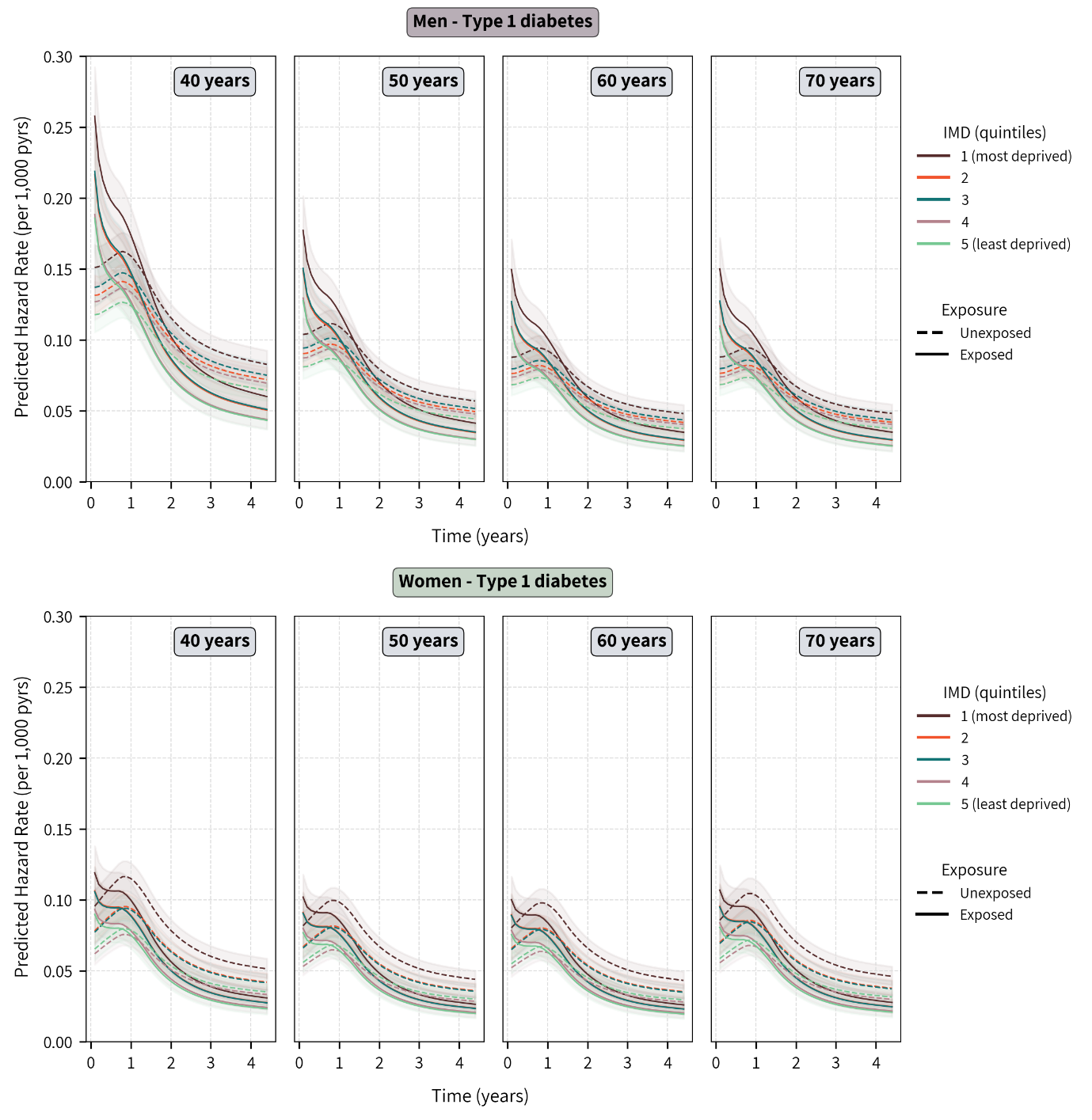


Sex-stratified predicted hazard rates obtained from flexible parametric survival models including natural cubic splines (4 degrees of freedom) of age and deprivation, an interaction between deprivation and exposure status, and a time-varying effect of the exposure. Time represents follow-up time from the index date (date of COVID-19 diagnosis for exposed individuals and the matched index date for unexposed individuals).

Solid lines represent exposed individuals, and dashed lines represent unexposed individuals. Shaded areas represent 95% confidence intervals.

Pyrs: person-years; BHF: British Heart Foundation; DDSC: Diabetes Data Science Catalyst.

# Figure S29: Hazard rates of type 1 diabetes (defined by BHF DDSC diabetes phenotyping algorithm) over time by ethnicity, age, and exposure status, and stratified by sex


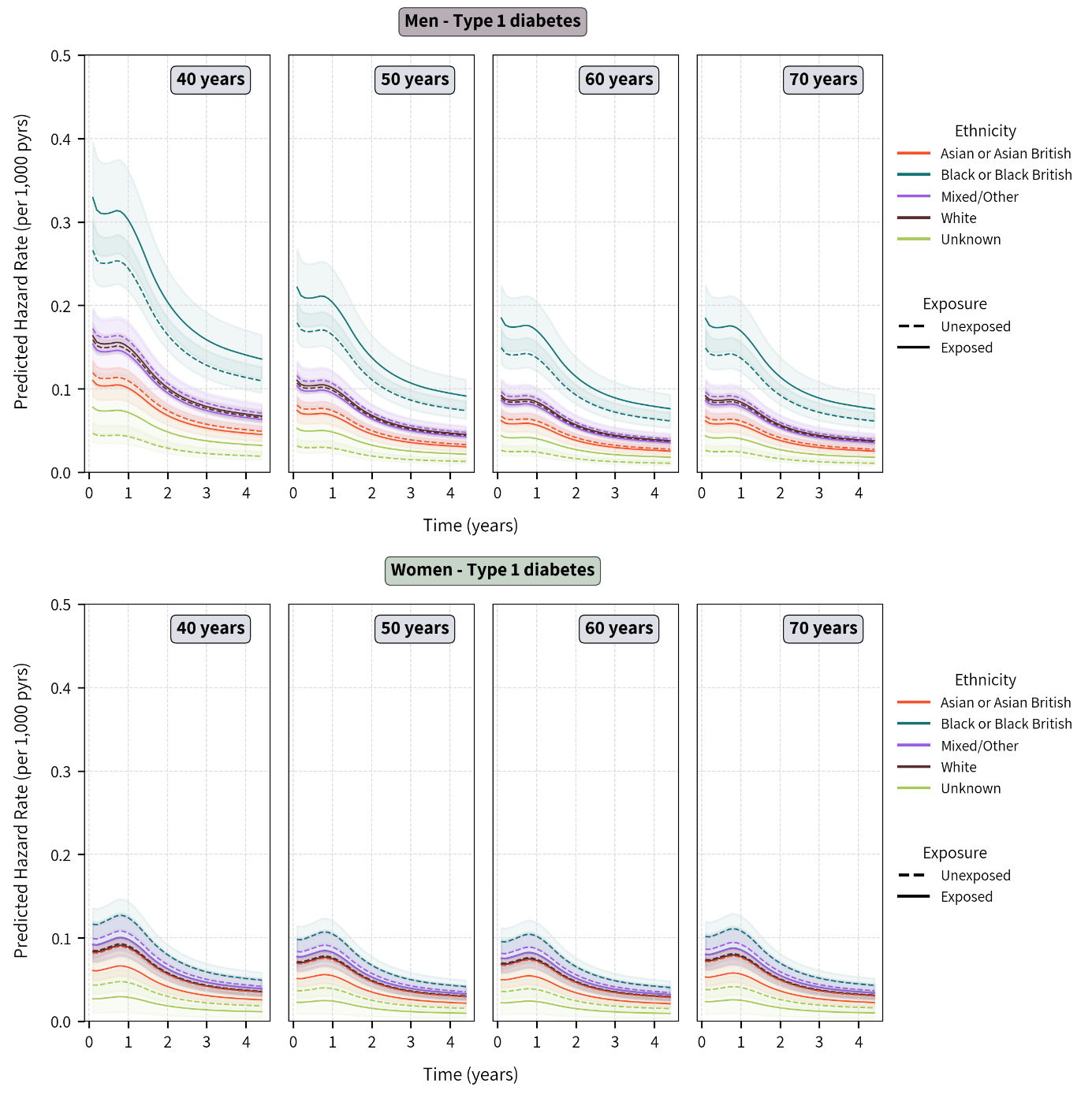


Sex-stratified predicted hazard rates obtained from flexible parametric survival models including natural cubic splines (4 degrees of freedom) of age and ethnicity and an interaction between ethnicity and exposure status. Time represents follow-up time from the index date (date of COVID-19 diagnosis for exposed individuals and the matched index date for unexposed individuals).

Solid lines represent exposed individuals, and dashed lines represent unexposed individuals. Shaded areas represent 95% confidence intervals.

Pyrs: person-years; BHF: British Heart Foundation; DDSC: Diabetes Data Science Catalyst.

# Figure S30: Hazard rates of type 1 diabetes (defined by BHF DDSC diabetes phenotyping algorithm) over time by ethnicity, age, and exposure status, with time-varying effects of exposure and stratified by sex


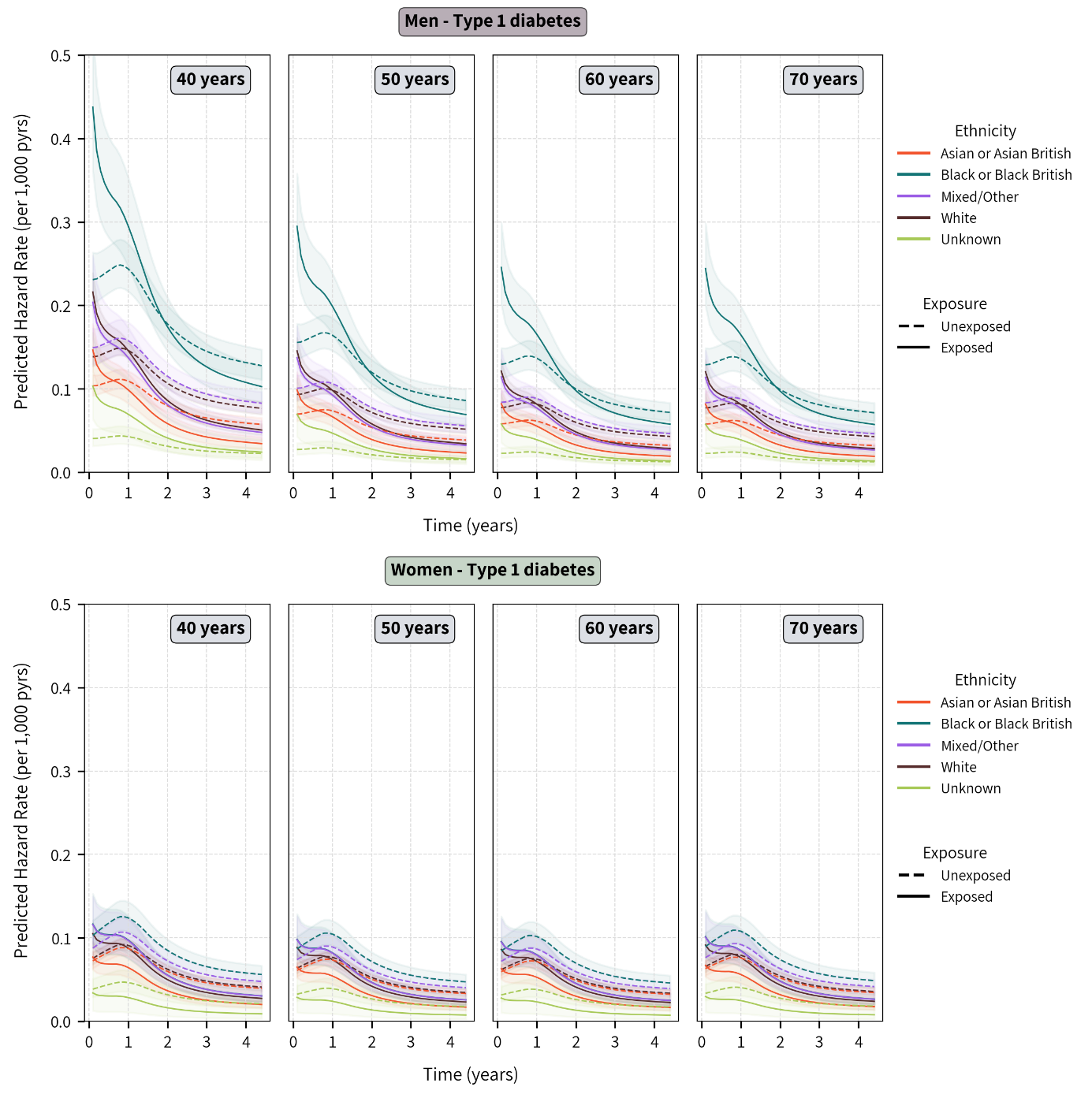


Sex-stratified predicted hazard rates obtained from flexible parametric survival models including natural cubic splines (4 degrees of freedom) of age and ethnicity, an interaction between ethnicity and exposure status, and a time-varying effect of the exposure. Time represents follow-up time from the index date (date of COVID-19 diagnosis for exposed individuals and the matched index date for unexposed individuals).

Solid lines represent exposed individuals, and dashed lines represent unexposed individuals. Shaded areas represent 95% confidence intervals.

Pyrs: person-years; BHF: British Heart Foundation; DDSC: Diabetes Data Science Catalyst.

# Figure S31: Hazard rates of type 1 diabetes (defined by BHF DDSC diabetes phenotyping algorithm) over time by region, age, and exposure status, and stratified by sex


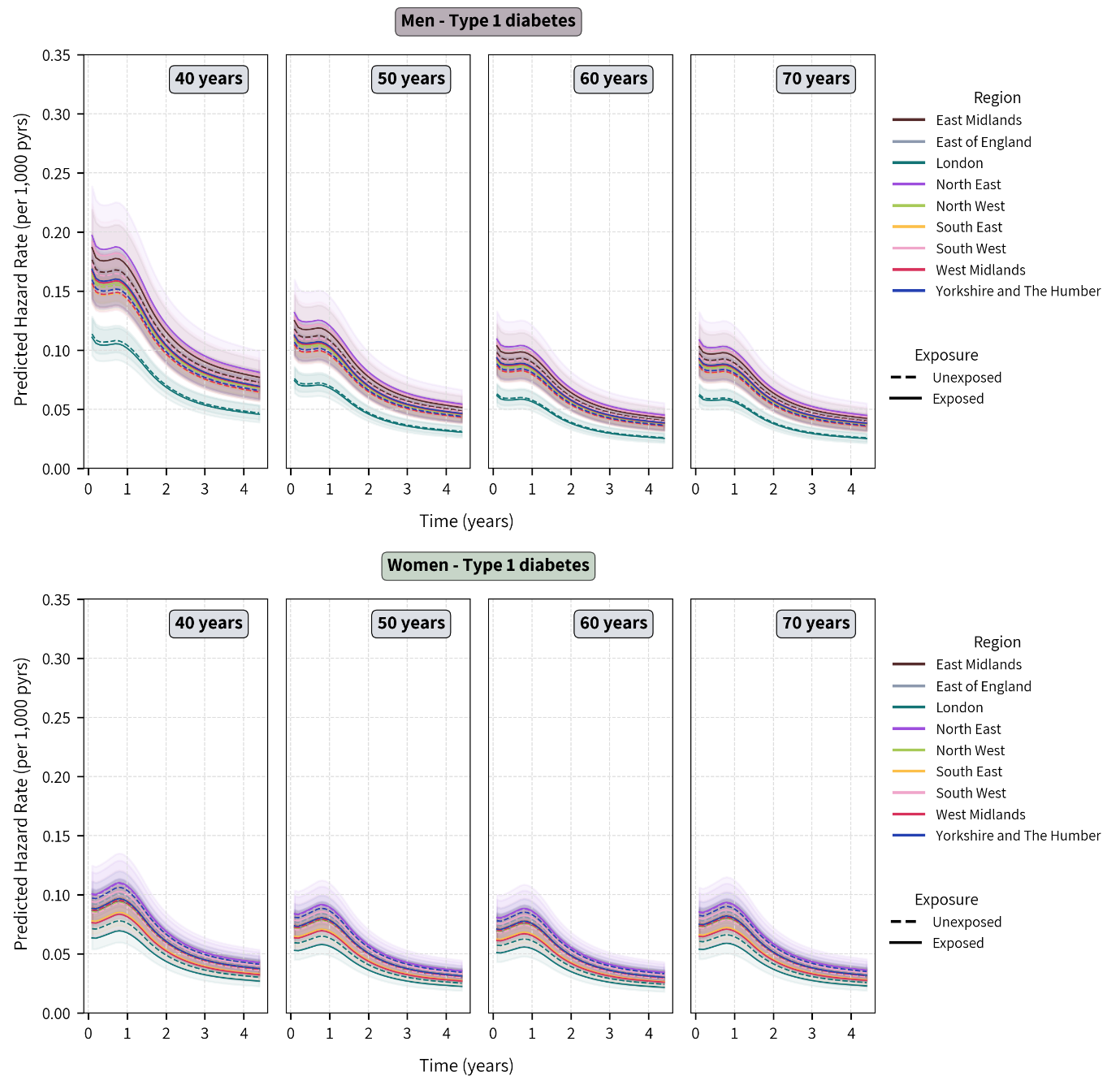


Sex-stratified predicted hazard rates obtained from flexible parametric survival models including natural cubic splines (4 degrees of freedom) of age and region and an interaction between region and exposure status. Time represents follow-up time from the index date (date of COVID-19 diagnosis for exposed individuals and the matched index date for unexposed individuals).

Solid lines represent exposed individuals, and dashed lines represent unexposed individuals. Shaded areas represent 95% confidence intervals.

Pyrs: person-years; BHF: British Heart Foundation; DDSC: Diabetes Data Science Catalyst.

# Figure S32: Hazard rates of type 1 diabetes (defined by BHF DDSC diabetes phenotyping algorithm) over time by region, age, and exposure status, with time-varying effects of exposure and stratified by sex


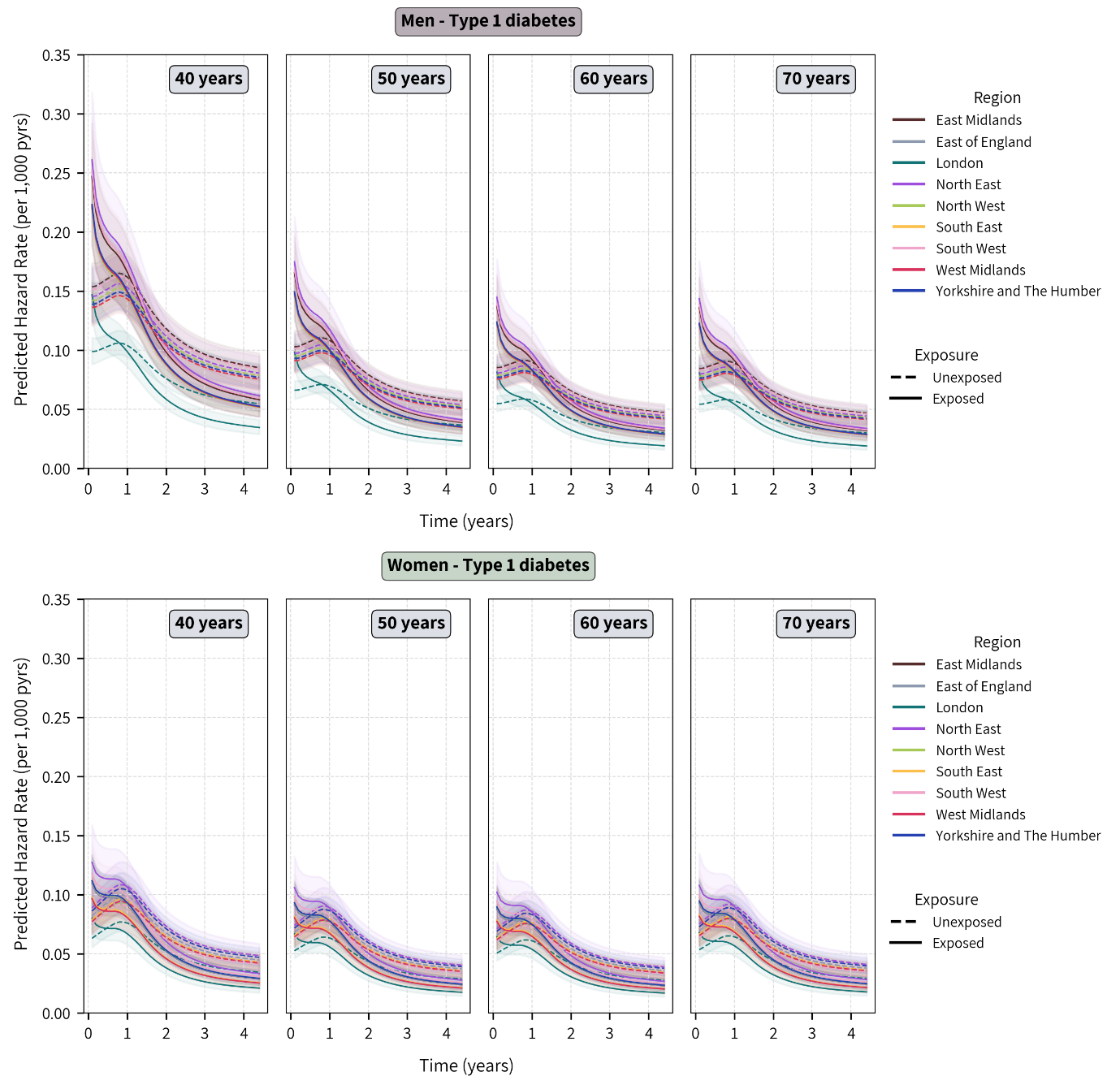


Sex-stratified predicted hazard rates obtained from flexible parametric survival models including natural cubic splines (4 degrees of freedom) of age and region, an interaction between region and exposure status, and a time-varying effect of the exposure. Time represents follow-up time from the index date (date of COVID-19 diagnosis for exposed individuals and the matched index date for unexposed individuals).

Solid lines represent exposed individuals, and dashed lines represent unexposed individuals. Shaded areas represent 95% confidence intervals.

Pyrs: person-years; BHF: British Heart Foundation; DDSC: Diabetes Data Science Catalyst.

# Figure S33: Hazard rates of type 2 diabetes over time among hospitalised COVID-19 patients and their matched controls by body mass index, age, and exposure status, and stratified by sex


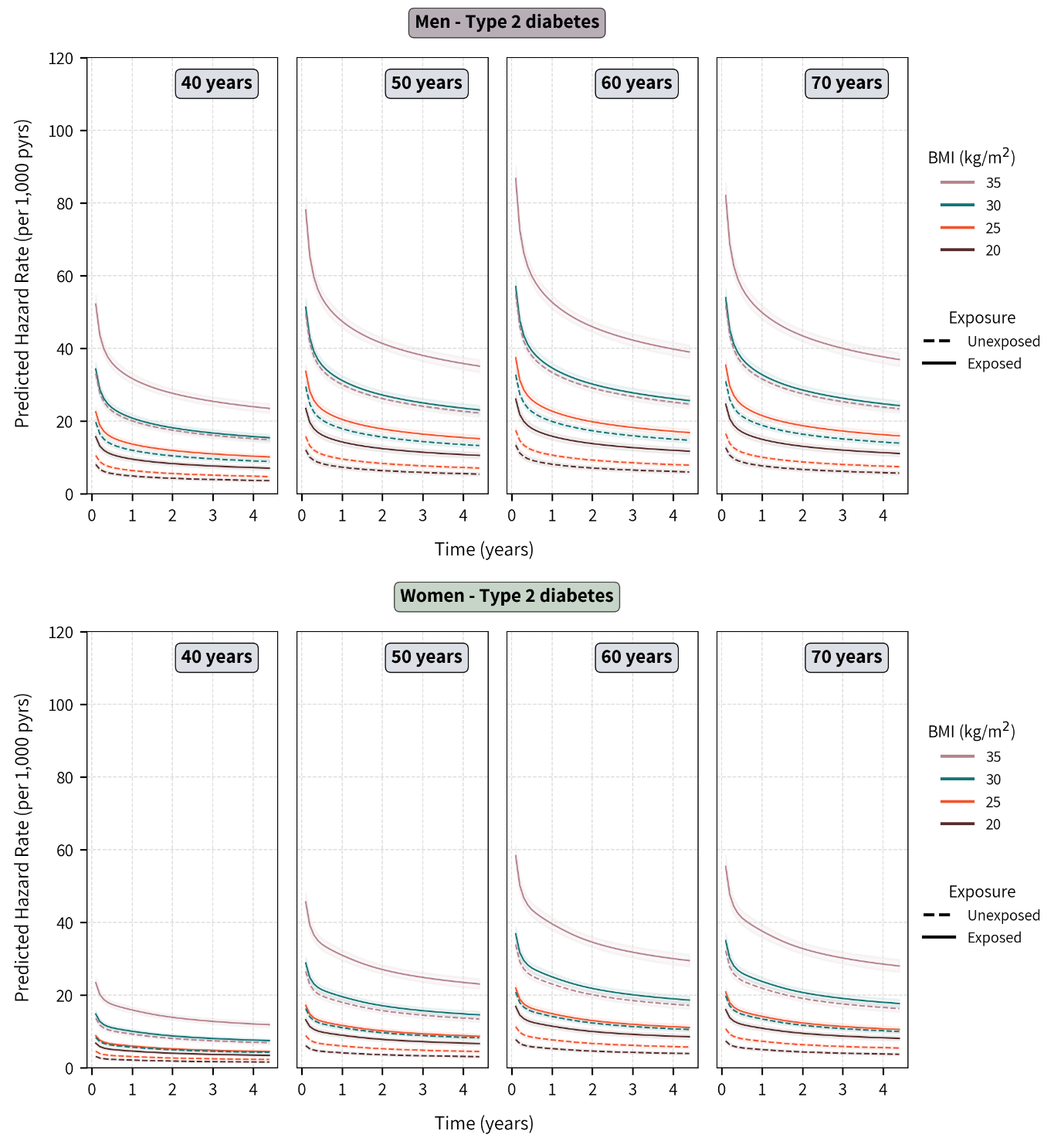


Sex-stratified predicted hazard rates obtained from flexible parametric survival models including natural cubic splines (4 degrees of freedom) of age and BMI, an interaction between BMI and exposure status. Time represents follow-up time from the index date (date of COVID-19 diagnosis in hospital records for exposed individuals and the matched index date for unexposed individuals).

Solid lines represent exposed individuals, and dashed lines represent unexposed individuals. Shaded areas represent 95% confidence intervals. BMI: Body mass index; Pyrs: person-years.

# Figure S34: Hazard rates of type 2 diabetes over time among hospitalised COVID-19 patients and their matched controls by deprivation, age, and exposure status, and stratified by sex


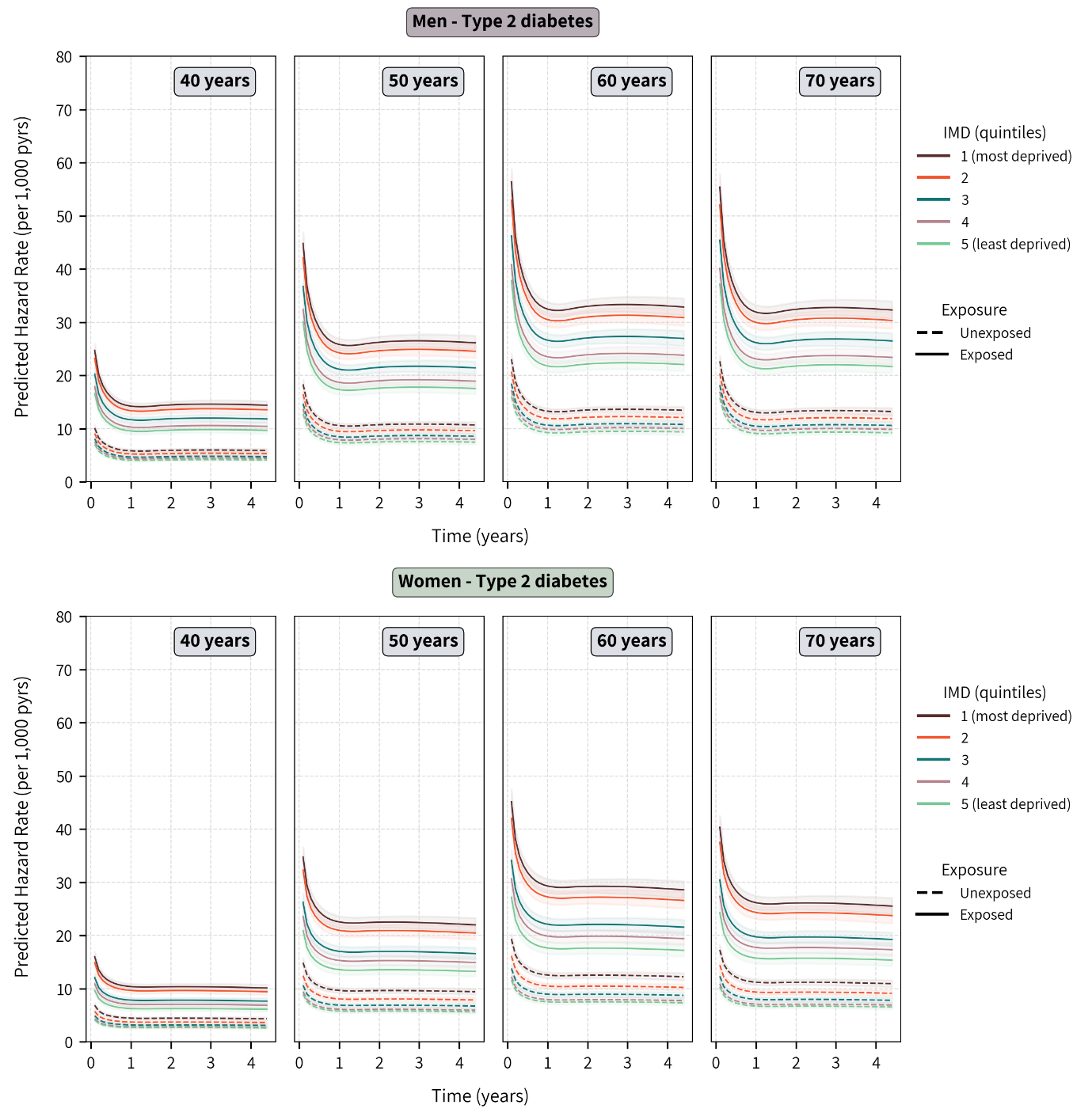


Sex-stratified predicted hazard rates obtained from flexible parametric survival models including natural cubic splines (4 degrees of freedom) of age and deprivation and an interaction between deprivation and exposure status. Time represents follow-up time from the index date (date of COVID-19 diagnosis in hospital records for exposed individuals and the matched index date for unexposed individuals).

Solid lines represent exposed individuals, and dashed lines represent unexposed individuals. Shaded areas represent 95% confidence intervals. Pyrs: person-years.

# Figure S35: Hazard rates of type 2 diabetes over time among hospitalised COVID-19 patients and their matched controls by ethnicity, age, and exposure status, and stratified by sex


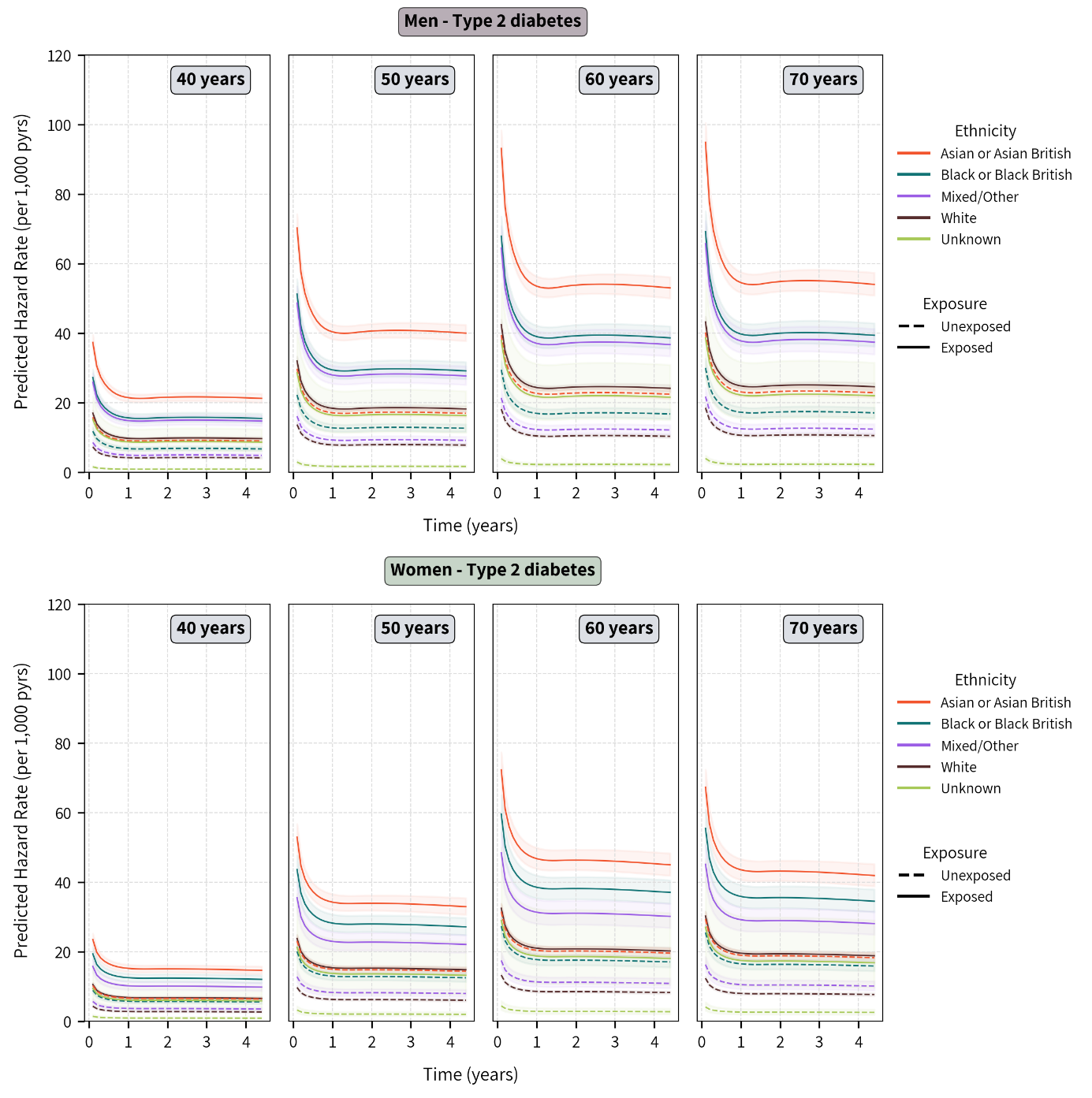


Sex-stratified predicted hazard rates obtained from flexible parametric survival models including natural cubic splines (4 degrees of freedom) of age and ethnicity and an interaction between ethnicity and exposure status. Time represents follow-up time from the index date (date of COVID-19 diagnosis in hospital records for exposed individuals and the matched index date for unexposed individuals).

Solid lines represent exposed individuals, and dashed lines represent unexposed individuals. Shaded areas represent 95% confidence intervals. Pyrs: person-years.

# Figure S36: Hazard rates of type 2 diabetes over time among hospitalised COVID-19 patients and their matched controls by region, age, and exposure status, and stratified by sex


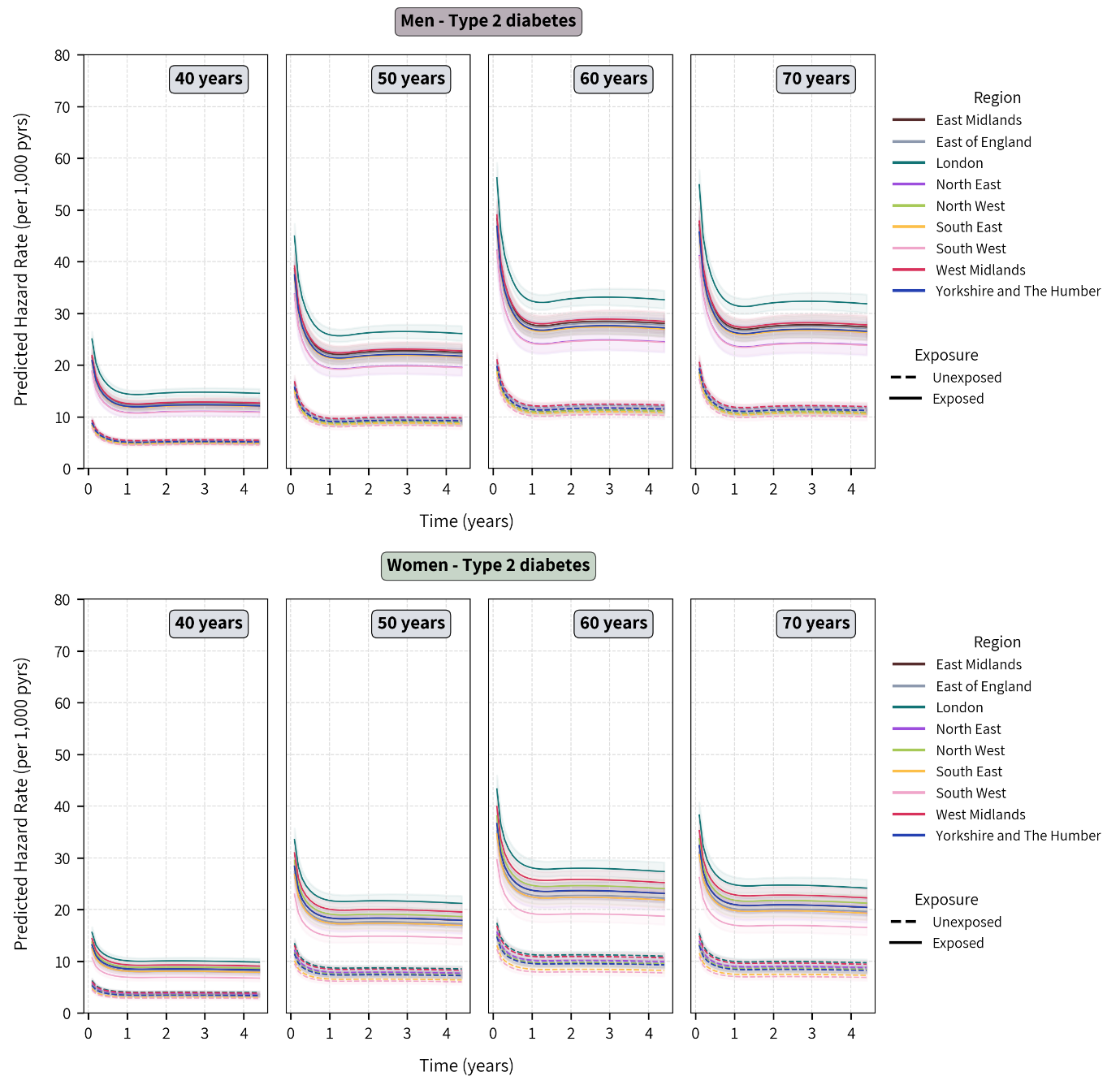


Sex-stratified predicted hazard rates obtained from flexible parametric survival models including natural cubic splines (4 degrees of freedom) of age and region and an interaction between region and exposure status. Time represents follow-up time from the index date (date of COVID-19 diagnosis in hospital records for exposed individuals and the matched index date for unexposed individuals).

Solid lines represent exposed individuals, and dashed lines represent unexposed individuals. Shaded areas represent 95% confidence intervals. Pyrs: person-years.

# Figure S37: Hazard rates of type 1 diabetes over time among hospitalised COVID-19 patients and their matched controls by body mass index, age, and exposure status, and stratified by sex


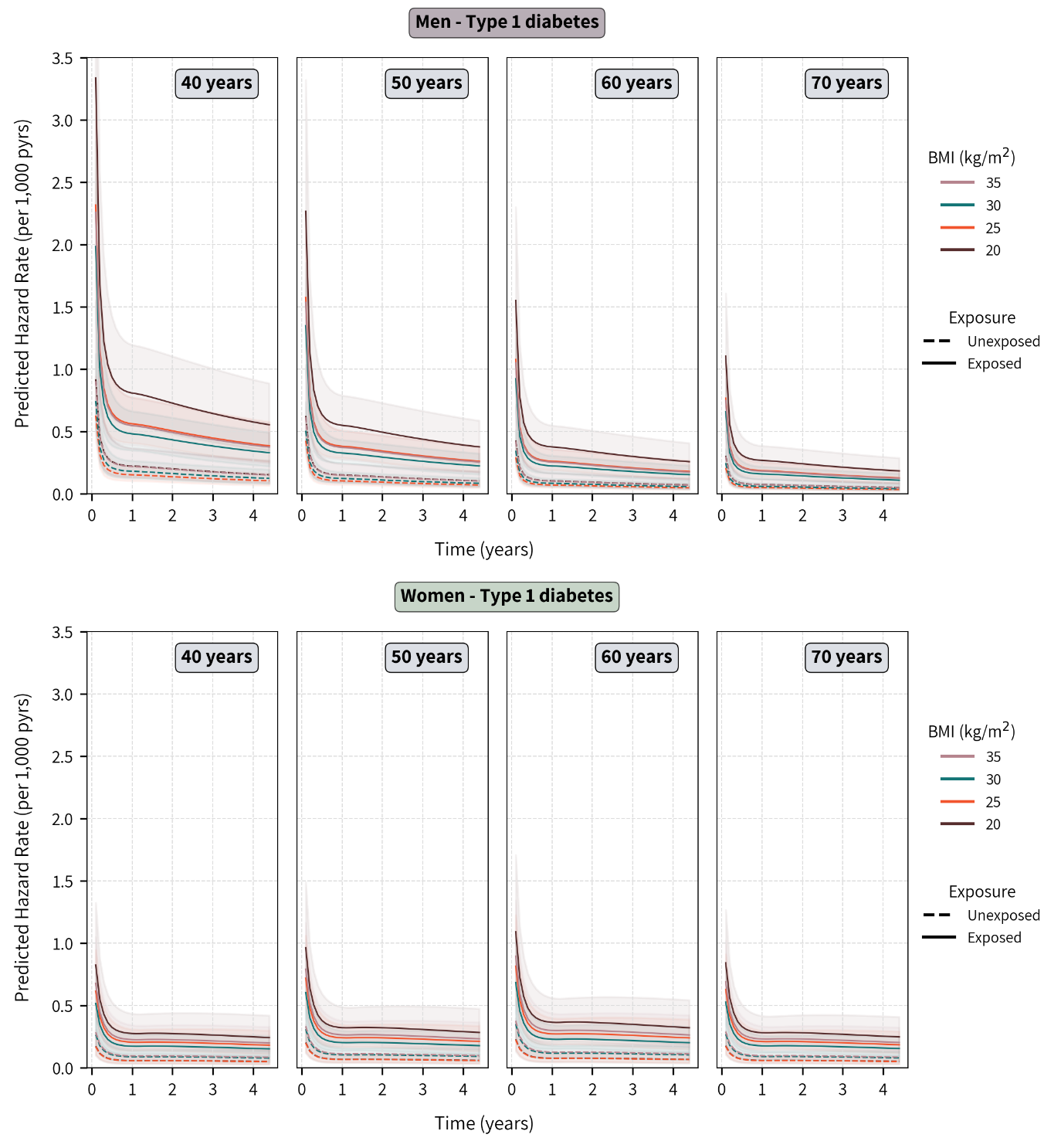


Sex-stratified predicted hazard rates obtained from flexible parametric survival models including natural cubic splines (4 degrees of freedom) of age and BMI, an interaction between BMI and exposure status. Time represents follow-up time from the index date (date of COVID-19 diagnosis in hospital records for exposed individuals and the matched index date for unexposed individuals).

Solid lines represent exposed individuals, and dashed lines represent unexposed individuals. Shaded areas represent 95% confidence intervals. BMI: Body mass index; Pyrs: person-years.

# Figure S38: Hazard rates of type 1 diabetes over time among hospitalised COVID-19 patients and their matched controls by deprivation, age, and exposure status, and stratified by sex


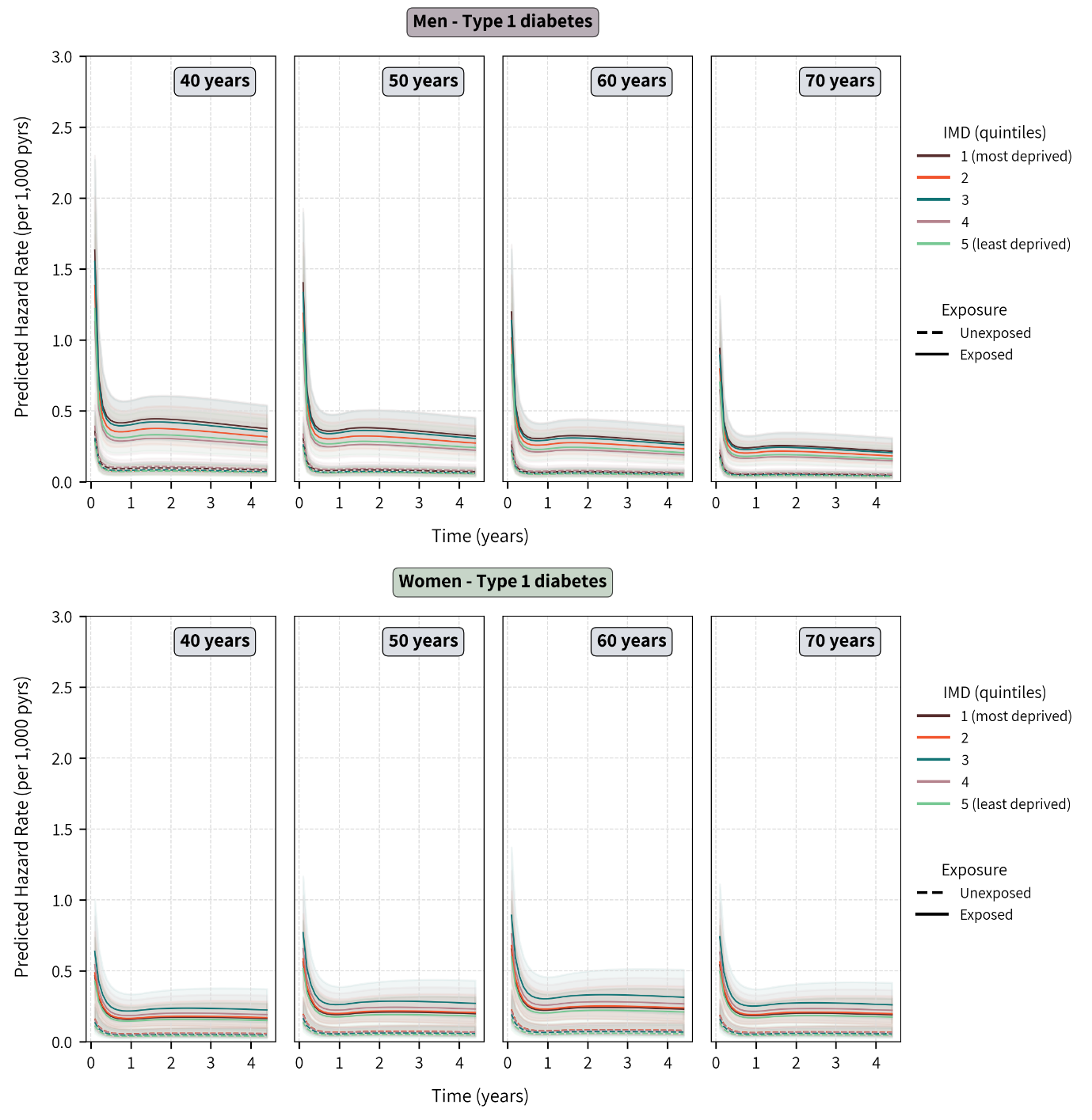


Sex-stratified predicted hazard rates obtained from flexible parametric survival models including natural cubic splines (4 degrees of freedom) of age and deprivation and an interaction between deprivation and exposure status. Time represents follow-up time from the index date (date of COVID-19 diagnosis in hospital records for exposed individuals and the matched index date for unexposed individuals).

Solid lines represent exposed individuals, and dashed lines represent unexposed individuals. Shaded areas represent 95% confidence intervals. Pyrs: person-years.

# Figure S39: Hazard rates of type 1 diabetes over time among hospitalised COVID-19 patients and their matched controls by ethnicity, age, and exposure status, and stratified by sex


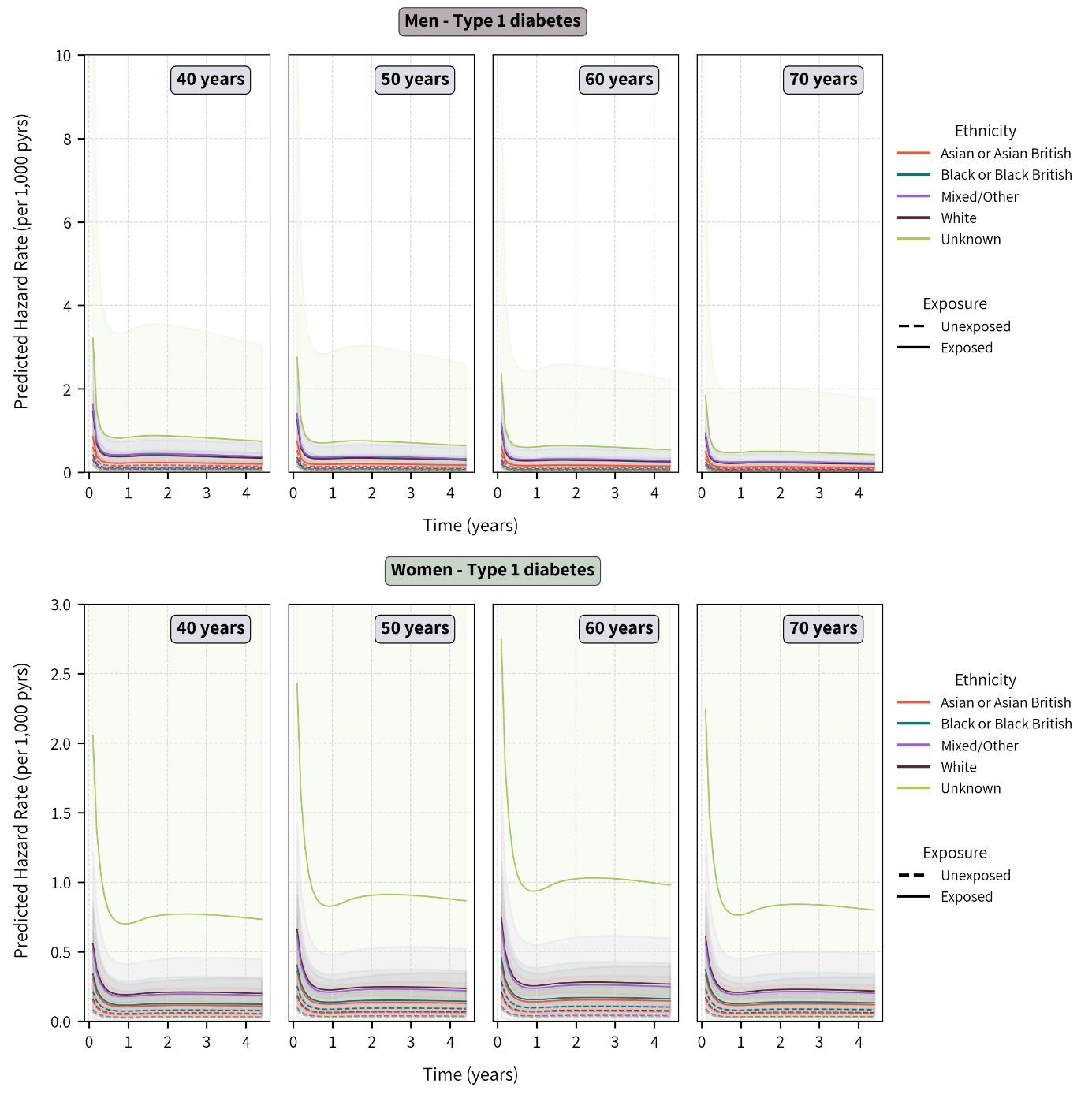


Sex-stratified predicted hazard rates obtained from flexible parametric survival models including natural cubic splines (4 degrees of freedom) of age and ethnicity and an interaction between ethnicity and exposure status. Time represents follow-up time from the index date (date of COVID-19 diagnosis in hospital records for exposed individuals and the matched index date for unexposed individuals).

Solid lines represent exposed individuals, and dashed lines represent unexposed individuals. Shaded areas represent 95% confidence intervals. Pyrs: person-years.

# Figure S40: Hazard rates of type 1 diabetes over time among hospitalised COVID-19 patients and their matched controls by region, age, and exposure status, and stratified by sex


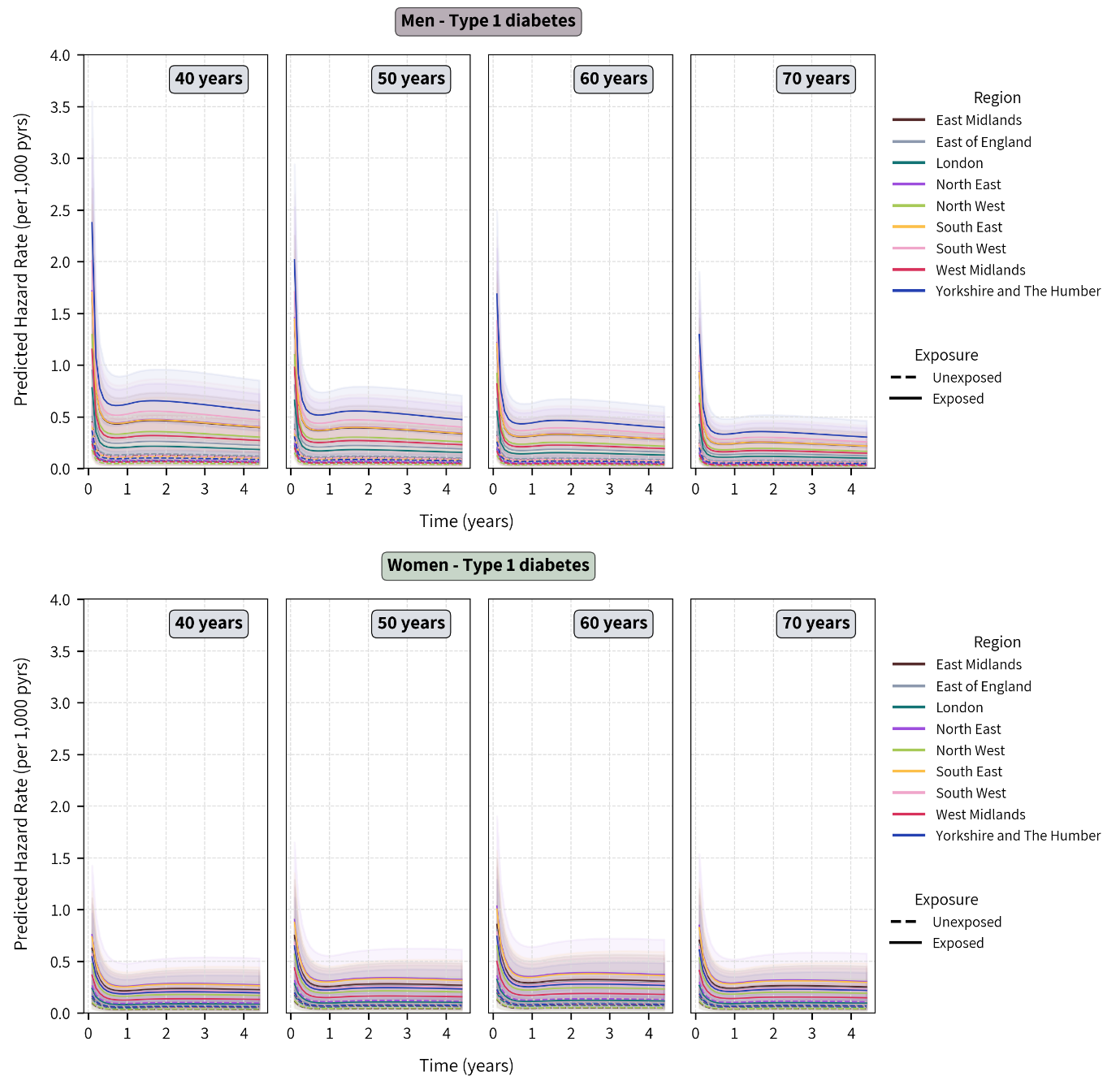


Sex-stratified predicted hazard rates obtained from flexible parametric survival models including natural cubic splines (4 degrees of freedom) of age and region and an interaction between region and exposure status. Time represents follow-up time from the index date (date of COVID-19 diagnosis in hospital records for exposed individuals and the matched index date for unexposed individuals).

Solid lines represent exposed individuals, and dashed lines represent unexposed individuals. Shaded areas represent 95% confidence intervals. Pyrs: person-years.

# The RECORD statement: Checklist of items, extended from the STROBE statement, that should be reported in observational studies using routinely collected health data.

|  | **Item No.** | **STROBE items** | **Location in manuscript where items are reported** | **RECORD items** | **Location in manuscript where items are reported** |
| --- | --- | --- | --- | --- | --- |
| **Title and abstract** | | | | | |
|  | 1 | (a) Indicate the study’s design with a commonly used term in the title or the abstract (b) Provide in the abstract an informative and balanced summary of what was done and what was found |  | RECORD 1.1: The type of data used should be specified in the title or abstract. When possible, the name of the databases used should be included.  RECORD 1.2: If applicable, the geographic region and timeframe within which the study took place should be reported in the title or abstract.  RECORD 1.3: If linkage between databases was conducted for the study, this should be clearly stated in the title or abstract. | Title Page and Page 2, Abstract  Page 2, Abstract  Page 2, Abstract |
| **Introduction** | | | | | |
| Background rationale | 2 | Explain the scientific background and rationale for the investigation being reported | Page 4, Introduction, 1st and 2nd paragraph |  |  |
| Objectives | 3 | State specific objectives, including any prespecified hypotheses | Page 4, Introduction, last paragraph |  |  |
| **Methods** | | | | | |
| Study Design | 4 | Present key elements of study design early in the paper | Pages 5-6, Methods |  |  |
| Setting | 5 | Describe the setting, locations, and relevant dates, including periods of recruitment, exposure, follow-up, and data collection | Pages 5-6, Methods |  |  |
| Participants | 6 | *(a) Cohort study* - Give the eligibility criteria, and the sources and methods of selection of participants. Describe methods of follow-up  *(b) Cohort study* - For matched studies, give matching criteria and number of exposed and unexposed |  | RECORD 6.1: The methods of study population selection (such as codes or algorithms used to identify subjects) should be listed in detail. If this is not possible, an explanation should be provided.  RECORD 6.2: Any validation studies of the codes or algorithms used to select the population should be referenced. If validation was conducted for this study and not published elsewhere, detailed methods and results should be provided.  RECORD 6.3: If the study involved linkage of databases, consider use of a flow diagram or other graphical display to demonstrate the data linkage process, including the number of individuals with linked data at each stage. | Pages 5-6, Methods and Supplementary Material  Pages 5-6, Methods  Figure S1 |
| Variables | 7 | Clearly define all outcomes, exposures, predictors, potential confounders, and effect modifiers. Give diagnostic criteria, if applicable. |  | RECORD 7.1: A complete list of codes and algorithms used to classify exposures, outcomes, confounders, and effect modifiers should be provided. If these cannot be reported, an explanation should be provided. | Pages 5-6, Methods |
| Data sources/ measurement | 8 | For each variable of interest, give sources of data and details of methods of assessment (measurement).  Describe comparability of assessment methods if there is more than one group | Pages 5-6, Methods |  |  |
| Bias | 9 | Describe any efforts to address potential sources of bias | Pages 5-6, Methods |  |  |
| Study size | 10 | Explain how the study size was arrived at | Pages 5-6, Methods  Figure S1 |  |  |
| Quantitative variables | 11 | Explain how quantitative variables were handled in the analyses. If applicable, describe which groupings were chosen, and why | Pages 5-6, Methods |  |  |
| Statistical methods | 12 | (a) Describe all statistical methods, including those used to control for confounding  (b) Describe any methods used to examine subgroups and interactions  (c) Explain how missing data were addressed  (d) *Cohort study* - If applicable, explain how loss to follow-up was addressed  (e) Describe any sensitivity analyses | Pages 5-7, Methods  Section, Statistical Analysis |  |  |
| Data access and cleaning methods |  | .. |  | RECORD 12.1: Authors should describe the extent to which the investigators had access to the database population used to create the study population.  RECORD 12.2: Authors should provide information on the data cleaning methods used in the study. | Page 15 Contribution statement  Page 5-7, Methods |
| Linkage |  | .. |  | RECORD 12.3: State whether the study included person-level, institutional-level, or other data linkage across two or more databases. The methods of linkage and methods of linkage quality evaluation should be provided. | Page 5-7, Methods |
| **Results** | | | | | |
| Participants | 13 | (a) Report the numbers of individuals at each stage of the study (*e.g.*, numbers potentially eligible, examined for eligibility, confirmed eligible, included in the study, completing follow-up, and analysed)  (b) Give reasons for non-participation at each stage.  (c) Consider use of a flow diagram |  | RECORD 13.1: Describe in detail the selection of the persons included in the study (*i.e.,* study population selection) including filtering based on data quality, data availability and linkage. The selection of included persons can be described in the text and/or by means of the study flow diagram. | Page 8, Results  Figure S1 |
| Descriptive data | 14 | (a) Give characteristics of study participants (*e.g.*, demographic, clinical, social) and information on exposures and potential confounders  (b) Indicate the number of participants with missing data for each variable of interest  (c) *Cohort study* - summarise follow-up time (*e.g.*, average and total amount) | Page 8, Results  Figure S1, Table 1  Table 2 |  |  |
| Outcome data | 15 | *Cohort study* - Report numbers of outcome events or summary measures over time | Page 8, Results  Table 2 |  |  |
| Main results | 16 | (a) Give unadjusted estimates and, if applicable, confounder-adjusted estimates and their precision (e.g., 95% confidence interval). Make clear which confounders were adjusted for and why they were included  (b) Report category boundaries when continuous variables were categorized  (c) If relevant, consider translating estimates of relative risk into absolute risk for a meaningful time period | Pages 8-10, Results |  |  |
| Other analyses | 17 | Report other analyses done—e.g., analyses of subgroups and interactions, and sensitivity analyses | Page 10, Results |  |  |
| **Discussion** | | | | | |
| Key results | 18 | Summarise key results with reference to study objectives | Page 11, Discussion |  |  |
| Limitations | 19 | Discuss limitations of the study, taking into account sources of potential bias or imprecision. Discuss both direction and magnitude of any potential bias |  | RECORD 19.1: Discuss the implications of using data that were not created or collected to answer the specific research question(s). Include discussion of misclassification bias, unmeasured confounding, missing data, and changing eligibility over time, as they pertain to the study being reported. | Pages 11-14, Discussion |
| Interpretation | 20 | Give a cautious overall interpretation of results considering objectives, limitations, multiplicity of analyses, results from similar studies, and other relevant evidence | Pages 11-14, Discussion |  |  |
| Generalisability | 21 | Discuss the generalisability (external validity) of the study results | Pages 11-14 Discussion,  Strengths and Limitations |  |  |
| **Other Information** | | | | | |
| Funding | 22 | Give the source of funding and the role of the funders for the present study and, if applicable, for the original study on which the present article is based | Page 15 Funding |  |  |
| Accessibility of protocol, raw data, and programming code |  | .. |  | RECORD 22.1: Authors should provide information on how to access any supplemental information such as the study protocol, raw data, or programming code. | Page 15 Data availability |

Benchimol EI, Smeeth L, Guttmann A, Harron K, Moher D, Petersen I, Sørensen HT, von Elm E, Langan SM; RECORD Working Committee. The REporting of studies Conducted using Observational Routinely-collected health Data (RECORD) statement. PLoS Med. 2015 Oct 6;12(10):e1001885. doi: 10.1371/journal.pmed.1001885. PMID: 26440803; PMCID: PMC4595218.

Checklist is protected under Creative Commons Attribution ([CC BY](http://creativecommons.org/licenses/by/4.0/)) license.

Number of pages refers to the original, Word document submission.
